# Pyridinylidenaminophosphines: Facile Access to Highly Electron‐Rich Phosphines

**DOI:** 10.1002/chem.201904621

**Published:** 2019-12-10

**Authors:** Philipp Rotering, Lukas F. B. Wilm, Janina A. Werra, Fabian Dielmann

**Affiliations:** ^1^ Institut für Anorganische und Analytische Chemie Westfälische Wilhelms-Universität Münster Corrensstrasse 30 48149 Münster Germany

**Keywords:** ligand design, phosphanes, phosphine ligands, strong donors, superbases

## Abstract

Electron‐rich tertiary phosphines are valuable species in chemical synthesis. However, their broad application as ligands in catalysis and reagents in stoichiometric reactions is often limited by their costly synthesis. Herein, we report the synthesis and properties of a series of phosphines with 1‐alkylpyridin‐4‐ylidenamino and 1‐alkylpyridin‐2‐ylidenamino substituents that are accessible in a very short and scalable route starting from commercially available aminopyridines and chlorophosphines. The determination of the Tolman electronic parameter (TEP) value reveals that the electron donor ability can be tuned by the substituent pattern at the aminopyridine backbone and it can exceed that of common alkylphosphines and N‐heterocyclic carbenes. The potential of the new phosphines as strong nucleophiles in phosphine‐mediated transformations is demonstrated by the formation of Lewis base adducts with CO_2_ and CS_2_. In addition, the coordination chemistry of the new phosphines towards Cu^I^, Au^I^, and Pd^II^ metal centers has been explored, and a convenient procedure to introduce the most basic phosphine into metal complexes starting from air‐stable phosphonium salt is described.

## Graphical abstract

**Highly electron‐rich phosphines** decorated with 1‐alkylpyridinylidenamino substituents (PyAPs) are accessible in one or two steps starting from inexpensive commercially available aminopyridines and chlorophosphines. The most basic PyAPs were used for the reversible CO_2_ fixation and the formation of electron‐rich metal complexes relevant to catalysis.

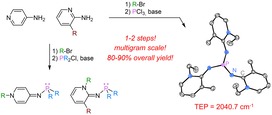

Tertiary phosphines are unique and diverse Lewis bases that are widely used in many areas of synthetic chemistry. Applications range from their utilization as ubiquitous ligands^1^ in coordination or organometallic chemistry to phosphine‐catalyzed^2^ and stoichiometric phosphine‐mediated^3^, ^4^ transformations. One of the main reasons for their broad range of applications is the intriguing ease with which the steric and electronic properties of phosphines can be rationally tuned using various substituents.^5^, ^6^ Although a huge variety of phosphines with diverse stereoelectronic properties have been synthesized over the last decades,^7^ the limit of electron‐donating character accessible to phosphines has been defined by alkylphosphines for more than half a century and modest advancement in this respect has been achieved using electropositive plumbyl,^8^ carboranyl,^9^ N‐heterocyclic boryl,^10^ anionic boratabenzene,^11^ or adamantyl^12^ substituents.

Recently, we discovered that the electron‐donating ability of phosphines can be significantly increased, beyond the range of N‐heterocyclic carbenes,^13^ by attaching up to three strong π‐donating imidazoline‐2‐ylidenamino substituents to the phosphorus atom.^14^ The resulting phosphines are promising ligands in catalysis and can activate and transform chemically rather inert species such as CO_2_ or SF_6_.^15^, ^16^, ^17^, ^18^, ^19^, ^20^

More recently, the family of highly electron‐rich phosphines with π‐donor substituents has been extended by the groups of Gessner and Sundermayer using phosphoniumylidyl (R_3_P=CR−) and phosphazenyl (R_3_P=N−) groups, respectively.^21^, ^22^ Although strongly donating phosphines have great potential as ligands in coordination chemistry and catalysis,^12^, ^23^ their broad application as ligands, but more importantly in stoichiometric reactions, is often hampered by their rather difficult synthesis. In this respect, readily available, cheap phosphines like PPh_3_ or P(*n*Bu)_3_ are typically used in phosphine‐mediated transformations such as Wittig,^24^ Mitzunobu,^25^ Appel,^26^ or Staudinger^27^ reactions.

Given these considerations, we envisaged that pyridinylidenaminophosphines (PyAPs) might be a potentially very useful family of electron‐rich phosphines owing to the following beneficial factors: 1) Aminopyridines are commercially available, cheap compounds which should enable a very short synthetic route to aminopyridin‐substituted phosphines; 2) the pyridinylidenamino groups can be regarded as remote carbene analogues of imidazoline‐2‐ylidenamino groups and should therefore similarly enhance the electron density at the phosphorus atom; 3) The selection of the R group at the pyridine N atom and the position relative to the exocyclic N should provide an easy means for stereoelectronic finetuning of the resulting phosphines (Figure 1 a).

**Figure 1:**
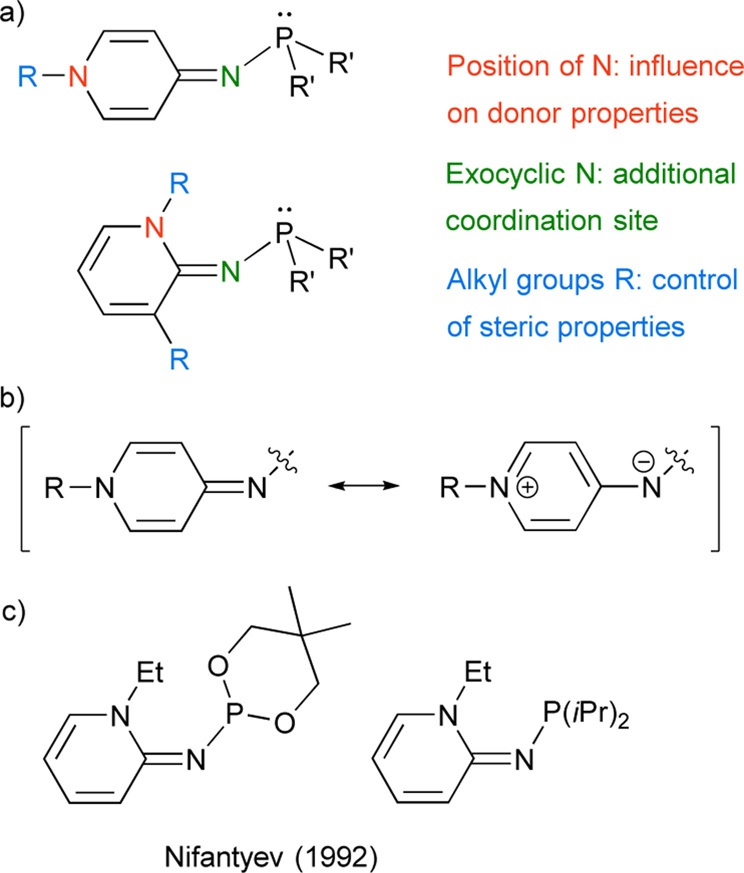
a) Structural features of pyridin‐4‐ylidenamino and pyridin‐2‐ylidenaminophosphines (PyAPs). b) Important resonance structures of the π‐donor substituents exemplified with the pyridin‐4‐ylidenamino group. c) Examples of isolated PyAPs.

With respect to the straightforward access, it is surprising that very little is known about the synthesis of PyAPs and their properties are unexplored: Nifantyev and co‐workers prepared two PyAPs from the reaction of 1‐ethylpyridin‐2‐imine with dialkylchlorophosphines when they studied the prototropic equilibrium of phosphorylated aminopyridines (Figure 1 c).^28^ Herein we report an easy synthetic access to a series of PyAPs and demonstrate their potential as strong nucleophiles for the activation of small molecules and as ligands in coordination chemistry.

We recently showed that tris(1‐butylpyridin‐4‐ylidenamino)phosphine can be generated in situ from the reaction of 1‐butylpyridin‐4‐imine with PCl_3_ and subsequent treatment with potassium bis(trimethylsilyl)amide (KHMDS).^18^ However, our attempts to isolate the phosphine were unsuccessful due to the formation of stable coordination compounds between the exocyclic N atoms of the pyridin‐4‐imine moieties and KCl formed in the reaction. Given the difficulties associated with the presence of several coordinatively accessible nitrogen donor atoms, we embarked our investigations with the synthesis of PyAPs carrying only one pyridinylidenamino substituent. Phosphines **2 a**–**d** were readily prepared by the reaction of pyridinium salts **1 a,b** with dialkylchlorophosphines using KHMDS as a base (Figure 2). The potassium salts can be separated by extraction with *n*‐hexane to afford **2 a**–**d** in very good yield. Furthermore, the synthesis of **2 a**–**d** can be carried out in a one‐pot procedure starting from 4‐aminopyridine, because the alkylation gives pyridinium salts **1 a,b** in quantitative yield.

**Figure 2:**
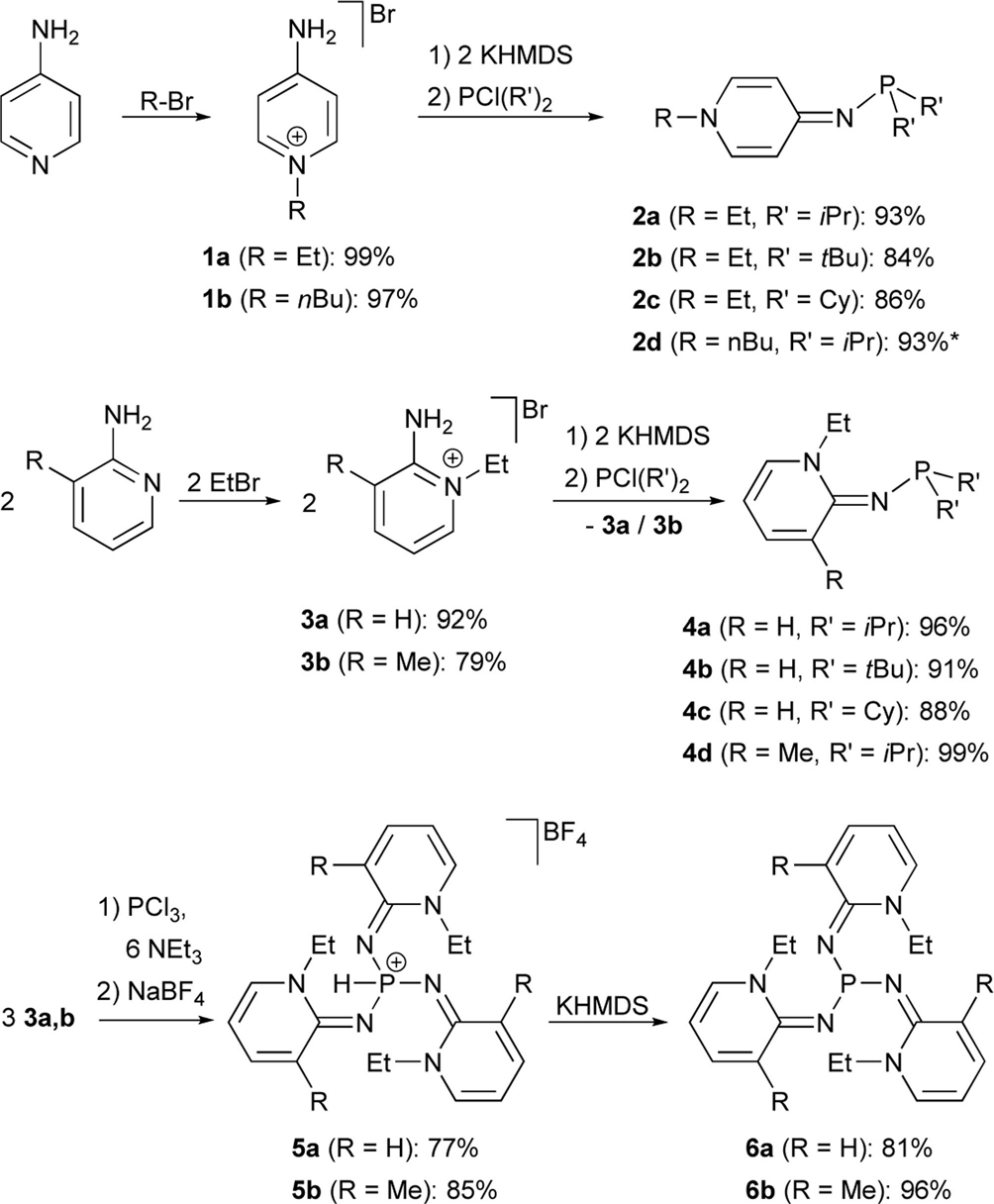
Synthesis of PyAPs starting from aminopyridines. *synthesis using excess imine as base.

Phosphines **4 a**–**d** had to be prepared in a slightly modified procedure because the sterically more encumbered 1‐alkylpyridin‐2‐imines, unlike 1‐alkylpyridin‐4‐imines, do not react with dialkyl(bis(trimethylsilyl)amino)phosphines. The latter are generated in situ from the reaction of KHMDS with dialkylchlorophosphines. For this reason, two equivalents of 1‐alkylpyridin‐2‐imines were reacted with dialkylchlorophosphines, which gave phosphines **4 a**–**d** in excellent yields. The second equivalent of the imine acts as a base and forms pyridinium salts **3**, which were recovered by extraction with dichloromethane and used for further reactions.

We next targeted the synthesis of PyAPs **6 a,b** containing three pyridin‐2‐ylidenamino substituents and hoped that the increased steric bulk around the exocyclic N atoms would destabilize coordination compounds with alkali‐metal salts. Treatment of PCl_3_ and three equivalents pyridinium salt **3 a,b** with triethylamine cleanly gave the protonated phosphines **5 a,b** in addition to triethylammonium halides (Figure 2). A precipitation step with NaBF_4_ from MeOH (**5 a**) or aqueous (**5 b**) solution leads to air‐stable and indefinitely storable phosphonium salts **5 a,b**. Gratifyingly, deprotonation with KHMDS and extraction with toluene afforded the salt‐free phosphines **6 a,b** in very good yield.

The phosphines carrying two alkyl groups and one pyridinylidenamino group were isolated as yellow solids (**2 a**–**d**, **4 b,c**) and yellow oils (**4 a**, **4 d**) and show good solubility in *n*‐hexane, toluene, Et_2_O, THF, and MeCN. In contrast, phosphines **6 a** and **6 b** were obtained as red wax‐like and crystalline solids, respectively. Note that the intense red color of **6 a** and **6 b** seems to be an intrinsic property of the free phosphines because it is not influenced by recrystallization steps and the color is quenched by protonation or carboxylation of the phosphorus atom (see below). The phosphine **6 a** and **6 b** are soluble in benzene, toluene, or THF but decompose rapidly in CH_2_Cl_2_, CHCl_3_ or MeCN owing to the basicity of the phosphorus atom. The ^31^P NMR resonances of **2 a**–**d** and **4 a**–**d** appear in the range of *δ*=51.5–71.5 ppm (Table 1).

**Table 1:** TEP values and ^31^P NMR resonances of PyAPs **2 a**–**c**, **4 a**–**d**, and **6 a**,**b** and selected phosphines for comparison.

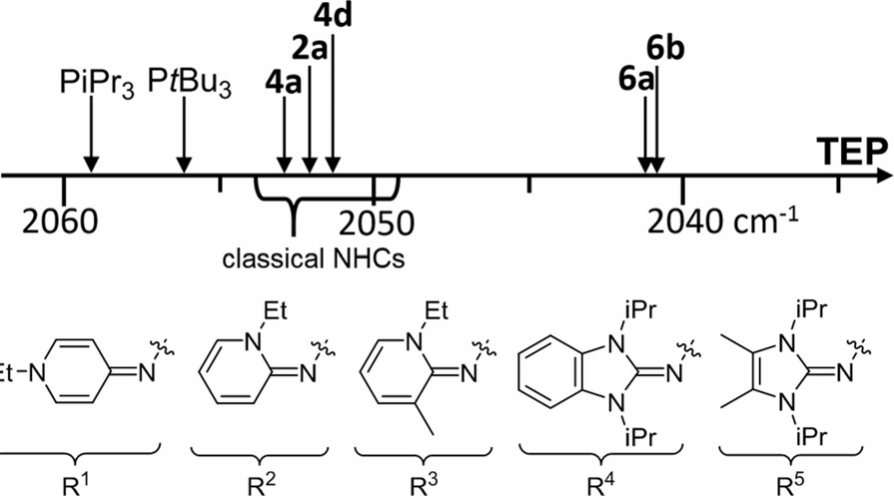		
Phosphine **L**	^31^P NMR resonance [ppm]	TEP [Ni(CO)_3_L]^[a]^
P(R^1^)*i*Pr_2_ (**2 a**)	56.5	2052.1/2049.2^[c]^
P(R^1^)*t*Bu_2_ (**2 b**)	68.9	2049.2
P(R^1^)Cy_2_ (**2 c**)	49.6	2050.8
P(R^2^)*i*Pr_2_ (**4 a**)	57.9	2052.9/2051.6^[c]^
P(R^2^)*t*Bu_2_ (**4 b**)	71.5	2051.3
P(R^2^)Cy_2_ (**4 c**)	51.5	2052.2
P(R^3^)*i*Pr_2_ (**4 d**)	63.9	2051.3/2049.6^[c]^
P(R^2^)_3_ (**6 a**)	86.4	2041.2
P(R^3^)_3_ (**6 b**)	83.5	2040.7
P(R^4^)*i*Pr_2_^[b]^	60.1	2053.6
P(R^5^)*i*Pr_2_^[b]^	63.2	2047.5

[a] Values in cm^−1^ recorded in CH_2_Cl_2_ solution. [b] Literature values.^14^, ^19^ [c] Recorded using solid samples.

Their chemical shift seems to be influenced primarily by the type of alkyl group and less by the pyridinylidenamino groups. The ^31^P NMR signals of phosphines **6 a** (83.5) and **6 b** (86.4 ppm) appear at higher frequencies. As a general trend for π‐donor substituents in PyAPs, the ^1^H NMR resonances of the pyridine protons are shifted to lower frequencies, in the range of olefinic protons, with increasing importance of the neutral imine‐type resonance structure (see Figure 1 b). For example, the chemical shift of the pyridine proton of phosphine **6 b** in 5‐position is shifted by Δ*δ*=1.12 ppm to higher frequencies upon protonation of the phosphorus atom.

The solid‐state structures of phosphines **2 d** and **6 b** were established by single‐crystal X‐ray diffraction (XRD) studies (Figure 3). The C1−N1 bond lengths in **2 d** (1.316) and **6 b** (1.284 Å) are in the range of elongated C−N double bonds (C=N: 1.29, C−N: 1.47 Å)^29^ as expected for the imine‐type resonance structure (see Figure 1 b). This observation agrees with the alternating C−C bond lengths in the pyridine rings (e.g. **2 b**: C1−C2 1.446, C2−C3 1.348 Å). As a result of the increased steric bulk around the exocyclic nitrogen atoms in **6 b** the P‐N1‐C1 bond angles (131.2°) are significantly larger than that in **2 b** (121.03°).

**Figure 3:**
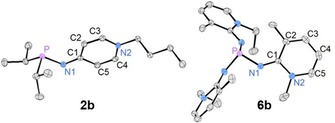
Molecular structures of **2 b** and **6 b**. The phosphorus atom of **6 b** is located on a threefold rotational axis. Hydrogen atoms are omitted for clarity; thermal ellipsoids are set at 50 % probability. Selected bond lengths [Å] and angles [°]: **2 b**: P−N1 1.7172(12), N1−C1 1.317(2), C1−C2 1.446(2), C2−C3 1.348(2), C3−N2 1.369(2), C4−N2 1.362(2), C4−C5 1.357(2), C1−C5 1.447(2), C1‐N1‐P 121.03(9). **6 b**: P−N1 1.7015(12), N1−C1 1.284(2), C1−C2 1.468(2), C2−C3 1.360(2), C3−C4 1.409(2), C4−C5 1.352(2), C5−N2 1.367(2), C5−N2 1.367(2), P‐N1‐C1 131.23(10).

To evaluate the electronic properties of the new PyAPs, we determined their Tolman electronic parameter^6^ by IR‐spectroscopic analysis of the corresponding complexes [Ni(CO)_3_(PyAP)] in dichloromethane (Table 1). The comparison of phosphines with two isopropyl groups and one N‐heterocyclic imine substituent reveals that the π‐donor ability of pyridinylidenamino groups (R^1^, R^2^, R^3^) is higher than that of the benzimidazolin‐2‐ylidenamino group R^4^ and lower than that of the 4,5‐dimethylimidazolin‐2‐ylidenamino group R^5^ according to the following trend: R^4^<R^2^<R^1^<R^3^≪R^5^. Owing to the negative inductive effect of the pyridine‐N atom, the TEP values of PyAPs with pyridin‐4‐ylidenamino groups are lower than those with pyridin‐2‐ylidenamino groups. Moreover, the lower TEP value of **4 d** and **6 a** compared with **4 a** and **6 b** can be attributed to the positive inductive effect of the additional methyl group. We recently showed that the π‐donor ability of substituents R^5^ strongly responds to the interaction of the exocyclic nitrogen atom with protons.^16^ To exclude that the TEP values might be influenced by such interactions with CH protons in dichloromethane, we recorded the IR spectra of the nickel complexes of phosphines **2 a**, **4 a**, and **4 d** in the solid state. In fact, the decrease in TEP values observed for the solid compounds (**4 a**: ΔTEP=1.3 cm, **4 d**: ΔTEP=1.7, **2 a**: ΔTEP=2.9 cm^−1^) suggests that the TEP values in CH_2_Cl_2_ are affected by the steric accessibility of the exocyclic nitrogen atom. Overall, the electron‐donating ability of PyAPs with two alkyl and one pyridinylidenamino group is in the range of classical N‐heterocyclic carbenes and of tri(1‐adamantyl)phosphine (TEP=2052.1 cm^−1^),^12^, ^30^, ^1^ whereas PyAPs with three donor groups (**6 a,b**) are significantly stronger donor ligands.

To get an insight into the nucleophilicity of PyAPs towards carbon‐based electrophiles, we explored the reactivity of phosphines **2 d** and **6 a** with CO_2_ (Figure 4). The recently identified correlation between the TEP value of phosphines and their CO_2_ binding energy shows that phosphine–CO_2_ adducts can be isolated if the phosphine has a lower TEP value than 2044 cm^−1^.^19^ In line with this result, pressurizing a THF solution of phosphine **6 a** with 2 bar CO_2_ resulted in the immediate precipitation of the CO_2_ adduct **7** as a pale yellow crystalline solid. The ^31^P NMR resonance of **7** (*δ*=−3.7 ppm) is significantly highfield shifted compared with that of **6 a** (*δ*=86.4 ppm) and the characteristic CO stretching band appears at 1620 cm^−1^ in the IR spectrum. The CO_2_ adduct **7** is stable at room temperature for days but decarboxylates rapidly when heated at 60 °C under vacuum, as indicated by the color change from pale yellow to red. The clean regeneration of **6 a** was verified by NMR spectroscopy.

**Figure 4:**
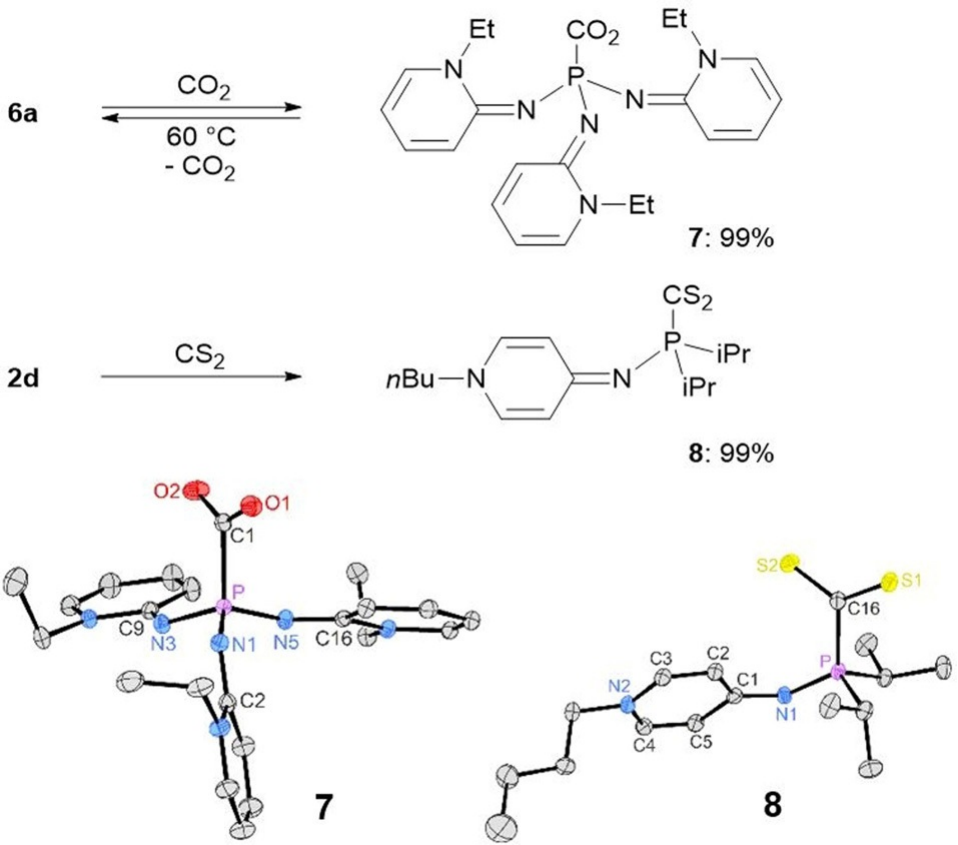
Synthesis of CO_2_ and CS_2_ adducts **7** and **8** (top) and their molecular structures in the solid state (bottom). Hydrogen atoms are omitted for clarity; thermal ellipsoids are set at 50 % probability. Selected bond lengths [Å] and angles [°]: **7**: P‐N1 1.6291(10), P−N3 1.6198(10), P−N5 1.6333(10), N1−C2 1.3175(13), N3−C9 1.314(2), N5−C16 1.3164(13). **8**: P−N1 1.6094(12), N1−C1 1.333(2), C1−C2 1.428(2), C2−C3 1.363(2), N2−C3 1.357(2), N2−C4 1.360(2), C4−C5 1.357(2), C1−C5 1.429(2), P‐N1‐C1 131.30(10).

In agreement with the lower basicity of **2 d**, the ^1^H and ^31^P NMR spectra of a THF solution of **2 d** pressurized with 2 bar CO_2_ were identical to those recorded without CO_2_ atmosphere, which indicates that **2 d** does not form persistent CO_2_ adducts. However, when **2 d** was treated with the more electrophilic CS_2_, a new ^31^P NMR resonance appeared at 28.7 ppm which is significantly highfield shifted compared with the free phosphine **2 d** (*δ*=56.4 ppm). The phosphine–CS_2_ adduct **8** was isolated as colorless solid in quantitative yield and shows a characteristic doublet (^1^ *J*_PC_=26.0 Hz) for the CS_2_ carbon atom in the ^13^C{^1^H} NMR spectrum.

XRD studies of **7** and **8** confirmed that CO_2_ and CS_2_ are bound to the phosphorus atoms with P−C bonds of 1.874 and 1.843 Å, respectively (Figure 4). The O‐C‐O angle of **7** (129.2°) is similar to that of O_2_C‐P(R^5^)_2_ *i*Pr (129.6°)^19^ and nitrogen base–CO_2_ adducts (128.6°–132.2°).^31^, ^32^ Compared to the free phosphines, the complexation of the Lewis acids CO_2_ and CS_2_ induces significant shortening of the P−N bonds (**6 b**: 1.702, **7**: av. 1.627; **2 d**: 1.717, **8**: 1.609 Å), suggesting a more pronounced N to P hyperconjugation.

To explore the coordination behavior of PyAPs, we prepared the Au^I^ complex [AuCl{P(R^3^)_3_}] (**9**), the Cu^I^ complex [Cu{P(R^3^)_3_}_2_] (**11**) and the Pd^II^ complex [Pd(allyl)Cl{P(R^3^)_3_}] (**10**) from the reaction of phosphine **6 b** with suitable precursors complexes (Figure 5). An alternative method to introduce **6 b** into metal complexes is based on the bench‐stable phosphonium salts **5 b**, thus avoiding the need for isolation of the highly air‐sensitive free phosphine **6 b**. After removing the volatiles under reduced pressure, complexes **9**–**11** were obtained as brown (**9**) and yellow (**10**, **11**) solids in quantitative yields. The compounds **9** and **10** are soluble in toluene, THF, CH_2_Cl_2_, or MeCN and can be stored in solution or in the solid state. The two‐coordinate Cu^I^ complex **11** however is highly reactive and decomposes rapidly in CH_2_Cl_2_, or MeCN. The ^31^P NMR signals of complexes **9** (26.6), **10** (34.8), and **11** (40.9 ppm) appear at lower frequencies than that of the free phosphine **6 b** (83.5 ppm), clearly indicating the coordination of the phosphorus atom. This connectivity was further confirmed by XRD analyses of **9** and **10**. Similar to the complexation of the Lewis acid CO_2_, the P−N bonds in **9** (1.641) and **10** (1.640 Å) are shorter than in the free phosphine **6 b** (1.702 Å) whereas the exocyclic C−N bonds (**9**: 1.307, **10**: 1.303 Å) are elongated (**6 b**: 1.284 Å). The steric demand of **6 b** was examined by calculation of the percent buried volume (%*V*_bur_)^33^, ^2^ giving larger values (**9**: 36.2 %, **10**: 35.3 %) than that of PPh_3_ (29.9 %) and smaller values than that of P*t*Bu_3_ (38.1 %) in complexes of the type [AuCl**L**].^34^

**Figure 5:**
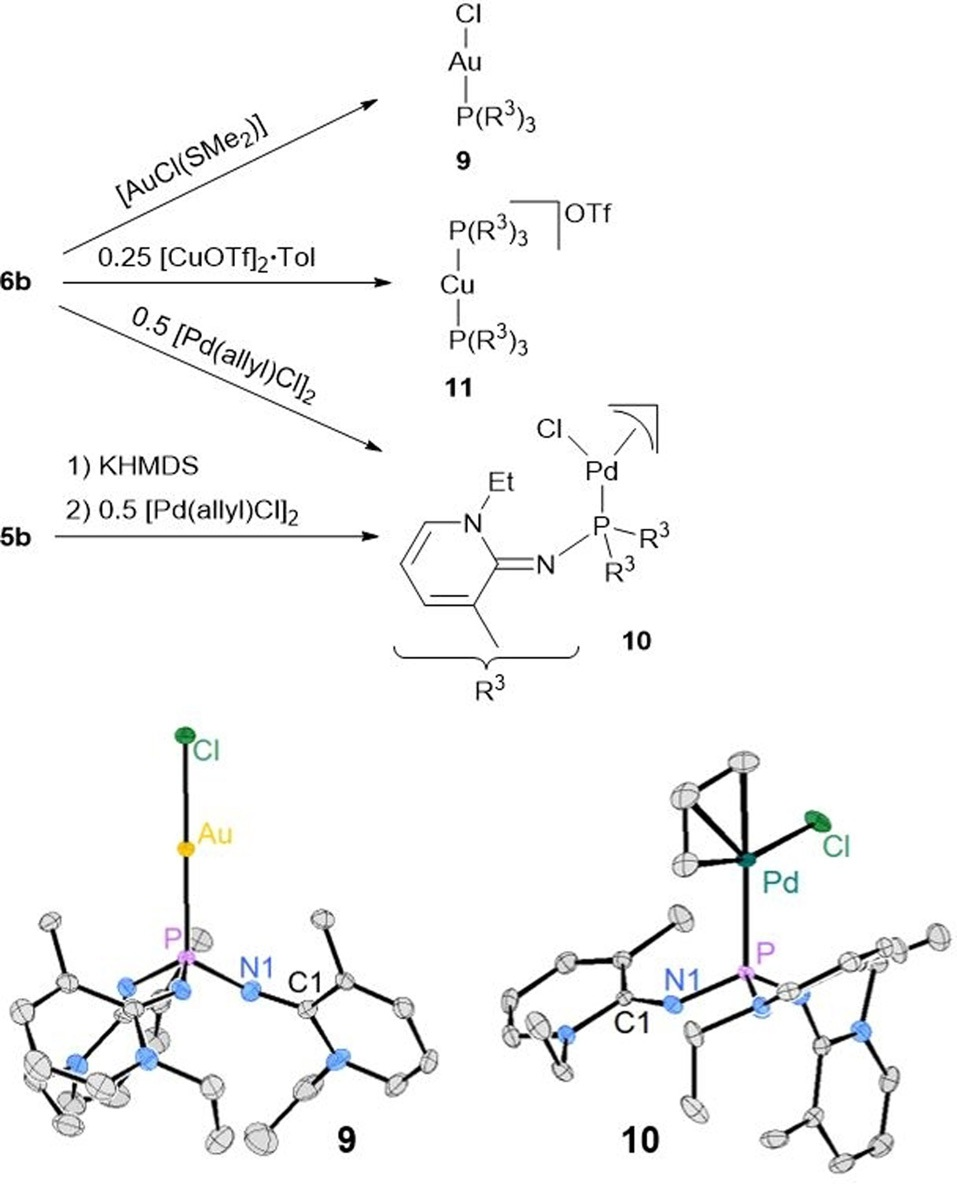
Synthesis of complexes **9**, **10** and **11** (top) and molecular structures of **9** and **10** (bottom). For **10** only one of the two independent molecules is depicted. Hydrogen atoms are omitted for clarity; thermal ellipsoids are set at 50 % probability. Selected bond lengths [Å] and angles [°]: **9**: P−Au 2.2478(9), P−N1 1.641(3), N1−C1 1.307(5), C1‐N1‐P 137.1(3). **10**: P−Pd 2.2885(12), P−N1 1.640(4), N1−C1 1.303(5), P‐N1‐C1 134.0(3).

In conclusion, an easy and scalable synthesis of highly electron rich phosphines carrying one (**2 a**–**d**, **4 a**–**d**) or three (**6 a,b**) 1‐alkylpyridinylidenamino substituents has been developed. The new phosphines are accessible in one or two steps starting from inexpensive commercially available aminopyridines and chlorophosphines. Spectroscopic data reveal that phosphines **2 a**–**d** and **4 a**–**d** are more electron donating than P(*t*Bu)_3_, whereas the ligand donor ability of **6 a,b** even exceeds that of N‐heterocyclic carbenes. Moreover, the stereoelectronic properties of PyAPs can be adjusted by the choice of the substituent pattern at the pyridine ring. The most basic PyAPs in this series were used for the reversible formation of CO_2_ adduct **7** and for the synthesis of representative transition‐metal complexes **9**, **10**, and **11**. The preparation of **11** from the air‐stable phosphonium salt **5 b** illustrates a convenient alternative to introduce the most basic phosphines into metal complexes. Given the combination of exceptional easy synthesis and highly basic character, PyAPs are not only appealing ligands in coordination chemistry and catalysis, but also provide new opportunities for stoichiometric phosphine‐mediated transformations.

## Experimental Section

Selected representative syntheses are presented below. For further information please see the Supporting Information.

**Pyridinium salt 1 a**: 4‐Aminopyridine (5.00 g, 53.1 mmol, 1 equiv.) and bromoethane (7.93 mL, 106 mmol, 2 equiv.) were dissolved in acetone (100 mL) and stirred for 16 h. The colorless solid was filtered off, washed with acetone (3×20 mL) and dried in vacuo at 120 °C for 16 h. Yield: 10.7 g (52.6 mmol, 99 %). ^1^H NMR ([D_6_]DMSO, 400 MHz): *δ*=8.26 (d, ^2^ *J*_HH_=6.9 Hz, 2 H, 2‐CH), 8.15 (s, 2 H, NH_2_), 6.88 (d, ^2^ *J*_HH_=6.9 Hz, 2 H, 3‐CH), 4.16 (q, ^2^ *J*_HH_=7.2 Hz, 2 H, CH_2_), 1.35 ppm (t, ^2^ *J*_HH_=7.2 Hz, 2 H, CH_3_). ^13^C{^1^H} NMR ([D_6_]DMSO, 100 MHz): *δ*=158.4 (s, 4‐C), 142.5 (s, 2‐C), 109.2 (s, 3‐C), 52.2 (s, CH_2_), 15.9 ppm (s, CH_3_). HRMS (ESI): *m*/*z* calculated for [C_7_H_11_N_2_]^+^ [M−Br]^+^ 123.09167, found 123.09159.

**General procedure a) for phosphine 4 a**–**d using excess imine as base**: The pyridinium salt (2 equiv.) and KHMDS (2 equiv.) were suspended in THF (10 mL mmol^−1^) and the mixture was stirred for 16 h. PClR_2_ (1 equiv.) was added dropwise at room temperature. After 3 h, all volatile compounds were removed in vacuo and the residue was extracted with *n*‐hexane (3×20 mL) to give the corresponding phosphine.

**General procedure b) for phosphine 2 a**–**c using KHMDS as base**: The pyridinium salt (1 equiv.), KHMDS (2 equiv.) and PClR_2_ (1 equiv.) were suspended in THF (10 mL mmol^−1^). After stirring the mixture for 3 h, all volatile compounds were removed in vacuo and the residue was extracted with *n*‐hexane (3×20 mL) to give the corresponding phosphine.

**Phosphine 2 a**: was prepared according to the general procedure b) using **1 a** (262 mg, 1.29 mmol), KHMDS (515 mg, 2.58 mmol) and PCl(*i*Pr)_2_ (2 mL, 0.645 m in toluene, 1.29 mmol). Yield: 285 mg (1.20 mmol, 93 %). ^1^H NMR ([D_6_]benzene, 400 MHz): *δ*=6.72 (dd, ^3^ *J*_HH_=7.9 Hz, ^4^ *J*_HH_=2.5 Hz, 2 H, 2‐H), 5.83 (d, ^3^ *J*_HH_=7.6 Hz, 2 H, 3‐H), 2.37 (q, ^3^ *J*_HH_=7.3 Hz, 2 H, CH_2_), 2.09 (hept, ^3^ *J*_HH_=7.1 Hz, 2 H, C*H*(CH_3_)_2_), 1.64–1.08 (m, 12 H, CH(C*H*_3_)_2_), 0.44 ppm (t, ^3^ *J*_HH_=7.3 Hz, 3 H, CH_3_). ^13^C{^1^H} NMR ([D_6_]benzene, 100 MHz): *δ*=166.6 (d, ^2^ *J*_PC_=17.6 Hz, 4‐C), 135.1 (d, ^4^ *J*_PC_=3.1 Hz, 2‐C), 116.3 (d, ^3^ *J*_PC_=23.1 Hz, 3‐C), 49.6 (s, CH_2_), 27.92 (d, *J=*10.9 Hz), 19.67 (d, *J=*19.3 Hz), 18.24 (d, *J=*8.8 Hz), 15.3 ppm (s, CH_3_). ^31^P NMR ([D_6_]benzene, 160 MHz): *δ*=56.5 ppm (s). CHN‐Analysis: found (calculated) C 65.27 (65.52) H 9.77 (9.73) N 11.88 (11.76). HRMS (ESI): *m*/*z* calculated for [C_13_H_24_N_2_P]^+^ [M+H]^+^ 239.16716, found 239.16664.

**Phosphine 4 a**: was prepared according to the general procedure a) using **3 a** (524 mg, 2.58 mmol), KHMDS (515 mg, 2.58 mmol) and PCl(*i*Pr)_2_ (2 mL, 0.645 m in toluene, 1.29 mmol). Yield: 296 mg (1.24 mmol, 96 %). ^1^H NMR ([D_6_]benzene, 400 MHz): *δ*=7.46 (m, 1 H, Ar‐H), 6.46 (ddd, ^3^ *J*_HH_=9.5 Hz, ^3^ *J*_HH_=6.3 Hz, ^4^ *J*_HH_=2.0 Hz, 1 H, Ar‐H), 6.25 (dd, ^3^ *J*_HH_=6.9 Hz, ^4^ *J*_HH_=1.9 Hz, 1 H, Ar‐H), 5.28 (ddd, ^3^ *J*_HH_=6.6 Hz, ^3^ *J*_HH_=6.6 Hz, ^4^ *J*_HH_=1.4 Hz, 1 H, Ar‐H), 3.56 (q, ^3^ *J*_HH_=7.1 Hz, 2 H, CH_2_), 1.91 (hept, ^3^ *J*_HH_=7.1 Hz, 2 H, C*H*(CH_3_)_2_), 1.23 (m, 12 H, CH(C*H*_3_)_2_), 0.99 ppm (t, ^3^ *J*_HH_=7.1 Hz, 3 H, CH_3_). ^13^C{^1^H} NMR ([D_6_]benzene, 100 MHz): *δ*=158.5 (d, ^2^ *J*_PC_=25.2 Hz, 2‐C), 137.3 (s, 6‐C), 134.1 (d, ^4^ *J*_PC_=4.0 Hz, 4‐C), 118.5 (d, ^3^ *J*_PC_=30.9 Hz, 3‐C), 102.4 (d, ^5^ *J*_PC_=1.6 Hz, 5‐C), 45.3 (s, CH_2_), 27.6 (d, *J=*11.2 Hz), 19.3 (d, *J=*20.1 Hz), 17.7 (d, *J=*8.3 Hz), 13.8 ppm (s, CH_3_). ^31^P NMR ([D_6_]benzene, 160 MHz): *δ*=57.9 ppm (s). HRMS (ESI): *m*/*z* calculated for [C_13_H_24_N_2_P]^+^ [M+H]^+^ 239.16716, found 239.16674.

**Phosphonium salt 5 b**: Pyridinium salt **3 b** (7.45 g, 34.30 mmol, 3 equiv.) and NEt_3_ (12.7 mL, 91.46 mmol, 8 equiv.) were dissolved in MeCN (30 mL). PCl_3_ (1.0 mL, 11.4 mmol, 1 equiv.) was added dropwise to the stirred solution at −35 °C and the reaction mixture was allowed to warm to room temperature. All volatile components were removed in vacuo and the residue was dissolved in H_2_O (50 mL). NaBF_4_ (1.88 g, 17.1 mmol, 1.5 equiv.) was added to the solution resulting in the precipitation of **5 b**. The precipitate was filtered off, washed with H_2_O and dried at 50 °C for 16 h in vacuo to afford **5 b** as a pale‐yellow solid. Yield: 5.10 g (9.72 mmol, 85 %). ^1^H NMR ([D_3_]MeCN, 400 MHz): *δ*=8.97 (d, ^1^ *J*_PH_=571.3 Hz, 1 H, PH), 7.61 (ddd, ^3^ *J*_HH_=6.8 Hz, ^4^ *J*_HH_=2.2 Hz, ^4^ *J*_HH_=2.2 Hz, 3 H, CH), 7.46 (ddd, ^3^ *J*_HH_=7.1 Hz, ^4^ *J*_HH_=1.4 Hz, ^4^ *J*_HH_=1.4 Hz, 3 H, CH), 6.49 (dd, ^3^ *J*_HH_=6.8 Hz, ^3^ *J*_HH_=6.8 Hz, 3 H, 5‐CH), 4.12 (q, ^3^ *J*_HH_=7.1 Hz, 6 H, CH_2_), 2.41 (s, 9 H, Ar‐CH_3_), 1.19 (t, ^3^ *J*_HH_=7.1 Hz, 9 H, CH_2_C*H*_3_) ppm. ^1^H{^31^P} NMR (MeCN‐*d*_3_, 400 MHz): *δ*=8.97 (s, PH), 7.62 (d, ^3^ *J*_HH_=6.6 Hz, CH), 7.45 (d, ^3^ *J*_HH_=7.1 Hz, CH), 6.49 (dd, ^3^ *J*_HH_=6.8 Hz, ^3^ *J*_HH_=6.8 Hz, 5‐CH), 4.12 (q, ^3^ *J*_HH_=7.1 Hz, CH_2_), 2.41 (s, Ar‐CH_3_), 1.19 ppm (t, ^3^ *J*_HH_=7.1 Hz, CH_2_C*H*_3_). ^11^B{^1^H} NMR ([D_3_]MeCN, 128 MHz): *δ*=−1.2 ppm (s). ^13^C{^1^H} NMR ([D_3_]MeCN, 100 MHz): *δ*=154.7 (d, ^2^ *J*_PC_=11.4 Hz, 2‐C), 140.1 (s, Ar‐C), 138.2 (d, *J*_PC_=1.1 Hz, Ar‐C), 131.8 (d, ^3^ *J*_PC_=3.4 Hz, 3‐C), 111.0 (s, 5‐C), 49.5 (s, CH_2_), 21.4 (d, ^4^ *J*_PC_=1.6 Hz, Ar‐CH_3_), 14.8 ppm (s, CH_2_ *C*H_3_). ^19^F NMR ([D_3_]MeCN, 376 MHz): *δ*=−151.8 (s, ^10^BF_4_), −151.8 ppm (s, ^11^BF_4_). ^31^P NMR ([D_3_]MeCN, 160 MHz): *δ*=−29.6 ppm (d, ^1^ *J*_PH_=571.6 Hz). ^31^P{^1^H} NMR ([D_3_]MeCN, 160 MHz): *δ*=−29.6 ppm (s). CHN‐Analysis: found (calculated) C 54.35 (54.97) H 6.45 (6.54) N 15.97 (16.03). HRMS (ESI): *m*/*z* calculated for [C_24_H_34_N_6_P]^+^ [M−BF_4_]^+^ 437.25881, found 437.25753.

**Phosphine 6 b**: Phosphonium salt **5 b** (858 mg, 1.64 mmol, 1 equiv.) and KHMDS (326 mg, 1.64 mmol, 1 equiv.) were suspended in toluene (10 mL) and stirred for 16 h. All volatile compounds were removed in vacuo and the residue was extracted with toluene (2×20 mL) to afford **6 b** as a dark red solid. Yield: 687 mg (1.57 mmol, 96 %). ^1^H NMR ([D_6_]benzene, 400 MHz): *δ*=6.53 (ddd, ^3^ *J*_HH_=6.3 Hz, ^4^ *J*_HH_=1.8 Hz, ^4^ *J*_HH_=1.8 Hz, 3 H, CH), 6.43 (dd, ^3^ *J*_HH_=6.7 Hz, ^4^ *J*_HH_=2.0 Hz, 3 H, CH), 5.37 (dd, ^3^ *J*_HH_=6.6 Hz, ^3^ *J*_HH_=6.6 Hz, 3 H, 5‐CH), 3.79 (q, ^3^ *J*_HH_=7.1 Hz, 6 H, CH_2_), 3.71 (m, 9 H, Ar‐CH_3_), 1.12 ppm (t, ^3^ *J*_HH_=7.0 Hz, 9 H, CH_2_C*H*_3_. ^13^C{^1^H} NMR ([D_6_]benzene, 100 MHz): *δ*=148.7 (d, ^2^ *J*_PC_=14.0 Hz, 2‐C), 135.6 (s, Ar‐C), 133.7 (s, Ar‐C), 130.9 (s, 3‐C), 101.5 (s, 5‐C), 46.6 (s, CH_2_), 24.1 (d, ^4^ *J*_PC_=28.1 Hz, Ar‐CH_3_), 14.7 ppm (d, ^5^ *J*_PC_=1.8 Hz, CH_2_ *C*H_3_. ^31^P NMR ([D_6_]benzene, 160 MHz): *δ*=83.5 ppm (s. CHN‐Analysis: found (calculated) C 65.04 (66.03) H 7.23 (7.62) N 18.89 (19.25). HRMS (ESI): *m*/*z* calculated for [C_24_H_34_N_6_P]^+^ [*M*+H]^+^ 437.25771, found 437.25772.

**Phosphine‐CO_2_ adduct 7**: Phosphine **6 a** (30 mg, 0.063 mmol) was dissolved in THF (5 mL). The degassed solution was pressurized with CO_2_ (2 bar) resulting in the precipitation of **7** as yellow solid, which was isolated after filtration and drying in vacuo in quantitative yield. ^1^H NMR ([D_3_]MeCN, 400 MHz): *δ*=7.55 (ddd, ^3^ *J*_HH_=6.8 Hz, ^4^ *J*_HH_=2.1 Hz, ^4^ *J*_PH_=2.1 Hz, 3 H, 3‐CH), 7.49 (d, ^3^ *J*_HH_=9.4 Hz, 3 H, 4‐CH), 7.32 (ddd, ^3^ *J*_HH_=8.9 Hz, ^3^ *J*_HH_=6.7 Hz, ^4^ *J*_HH_=1.9 Hz, 3 H, 6‐CH), 6.33 (ddd, ^3^ *J*_HH_=6.7 Hz, ^3^ *J*_HH_=6.7 Hz, ^4^ *J*_HH_=1.5 Hz, 3 H, 5‐CH), 4.12 (q, ^3^ *J*_HH_=7.1 Hz, 6 H, CH_2_), 1.31 ppm (t, ^3^ *J*_HH_=7.1 Hz, 9 H, CH_3_). ^13^C{^1^H} NMR ([D_3_]MeCN, 100 MHz): *δ*=156.7 (d, ^2^ *J*_PC_=5.8 Hz, 2‐C), 138.6 (d, *J*_PC_=1.8 Hz, Ar‐C), 137.4 (s, Ar‐C), 121.7 (d, *J*_PC_=7.8 Hz, Ar‐C), 108.2 (s, 5‐C), 46.9 (s, CH_2_), 13.9 ppm (s, CH_3_. ^31^P NMR ([D_3_]MeCN, 160 MHz): *δ*=−3.7 ppm (s). HRMS (ESI): *m*/*z* calculated for [C_21_H_28_N_6_P]^+^ [M−CO_2_+H]^+^ 395.21076, found 395.21250. IR (neat): $\tilde{\nu }$ =2976 (w), 2932 (w), 1632 (s), 1566 (w), 1541 (m), 1505 (vs.), 1456 (s), 1389 (s), 1349 (m), 1290 (m), 1273 (m), 1164 (m), 1144 (w), 1032 cm^−1^ (m).

**Gold(I) complex 9**: A solution of **6 b** (50 mg, 0.115 mmol, 1 equiv.) in toluene (2 mL) was added to a stirred suspension of [AuCl(SMe_2_)] (34 mg, 0.115 mmol, 1 equiv.) in toluene (2 mL) at −40 °C. The solution was allowed to warm to room temperature and stirred for 16 h. All volatile compounds were removed in vacuo to afford **9** as brown solid in quantitative yield. ^1^H NMR (CD_2_Cl_2_, 400 MHz): *δ*=7.23 (dddd, ^3^ *J*_HH_=6.9 Hz, *J=*2.4 Hz, *J=*1.2 Hz, *J=*1.2 Hz, 3 H, CH), 7.08 (m, 3 H, CH), 6.06 (dd, ^3^ *J*_HH_=6.7 Hz, ^3^ *J*_HH_=6.7 Hz, 3 H, 5‐CH), 4.01 (q, ^3^ *J*_HH_=7.1 Hz, 6 H, CH_2_), 2.71 (d, *J=*1.1 Hz, 9 H, Ar‐CH_3_), 1.17 ppm (t, ^3^ *J*_HH_=7.1 Hz, 9 H, CH_2_C*H*_3_). ^13^C{^1^H} NMR (CD_2_Cl_2_, 100 MHz): *δ*=151.5 (d, ^2^ *J*_PC_=5.3 Hz, 2‐C), 137.4 (s, Ar‐C), 136.1 (s, Ar‐C), 131.7 (d, ^3^ *J*_PC_=3.9 Hz, 3‐C), 106.6 (s, 5‐C), 47.9 (s, CH_2_), 23.7 (d, ^4^ *J*_PC_=3.6 Hz, Ar‐CH_3_), 14.8 ppm (s, CH_2_ *C*H_3_). ^31^P NMR (CD_2_Cl_2_, 160 MHz): *δ*=26.6 ppm (s). HRMS (ESI): *m*/*z* calculated for [C_24_H_34_N_6_PAuCl]^+^ [M+H]^+^ 669.19311, found 669.19640.

## Conflict of interest

The authors declare no conflict of interest.

## Supporting information

As a service to our authors and readers, this journal provides supporting information supplied by the authors. Such materials are peer reviewed and may be re‐organized for online delivery, but are not copy‐edited or typeset. Technical support issues arising from supporting information (other than missing files) should be addressed to the authors.

Supplementary

## Footnotes

1 TEP of PAd_3_ was calculated using [Rh(acac)(CO)(L)]

2
*r*=3.5 Å, *d*=2.28 Å, bond radii are scaled to 1.17
