# Supplementary material for: Pyridinylidenaminophosphines: Facile Access to Highly Electron‐Rich Phosphines
Source: Chemistry. 2019 Dec 10;26(2):406–11. doi: 10.1002/chem.201904621 (PMC6972615; doi:10.1002/chem.201904621)
Supplement: Supplementary file 1 — Supplementary [file CHEM-26-406-s001.pdf]

CHEMISTRY
A European Journal

Supporting Information

Pyridinylidenaminophosphines: Facile Access to Highly Electron-Rich Phosphines

Philipp Rotering, Lukas F. B. Wilm, Janina A. Werra, and Fabian Dielmann*[a]

chem_201904621_sm_miscellaneous_information.pdf

CONTENTS:

**Synthetic Details**	**2**
**Synthesis of pyridinium salts 1a-b and 3a-b**	**3**
**Synthesis of phosphines 2a-d and 4a-d**	**9**
**Synthesis of 5a-b and 6a-b**	**25**
**Synthesis of 7 and 8**	**38**
**Synthesis of metal complexes 9-11**	**43**
**Preparation of [Ni(CO)$_3$(PR$_3$)]**	**49**
**X-ray Diffraction Studies**	**56**

Synthetic Details

General remarks: All manipulations were performed under an inert atmosphere of dry argon, using standard Schlenk and drybox techniques. Dry and oxygen-free solvents were employed. All glassware was oven-dried at 160 °C prior to use. ^{1}H, ^{13}C and ^{31}P NMR spectra were recorded at 300 K on Agilent DD2 600, Bruker AVANCE I 400, Bruker AVANCE III 400 or Bruker AVANCE II 200 spectrometers. Chemical shifts are given in parts per million (ppm) relative to $SiMe_4$ (^{1}H, ^{13}C), 85% H_3PO_4 (^{31}P) and they were referenced to the residual solvent signals (C_6D_6: ^{1}H δ_H = 7.16, ^{13}C δ_C = 128.06; CD_3CN: δ_H = 1.94, ^{13}C δ_C = 118.26; THF-d_8: ^{1}H δ_H = 1.73, ^{13}C δ_C = 67.57) or internally by the instrument after locking and shimming to the deuterated solvent (^{31}P). Chemical shifts (δ) are reported in ppm. NMR multiplicities are abbreviated as follows: s = singlet, d = doublet, t = triplet, p = pentet, sept = septet, m = multiplet, br = broad signal. Mass spectrometry was recorded using an Orbitrap LTQ XL (Thermo Scientific) spectrometer. IR spectra were obtained on a Bruker Alpha Spectrometer.

Reagents and Handling: All compounds were purchased from commercial sources (Sigma Aldrich, Alfa Aesar, abcr GmbH) and used as received.

Synthesis of pyridinium salts **1a-b** and **3a-b**

Compound 1a

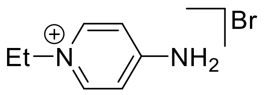

4-Aminopyridine (5.00 g, 53.1 mmol, 1 eq.) and bromoethane (7.93 mL, 106 mmol, 2 eq.) were dissolved in acetone (100 mL) and stirred for 16 h. The colourless solid was filtered off, washed with acetone (3 x 20 mL) and dried *in vacuo* at 120 °C for 16 h. Yield: 10.7 g (52.6 mmol, 99%).

^{1}H NMR (DMSO-d_6, 400 MHz) δ (ppm) = 8.26 (d, $^2J_{HH}$ = 6.9 Hz, 2H, 2-CH), 8.15 (s, 2H, NH_2), 6.88 (d, $^2J_{HH}$ = 6.9 Hz, 2H, 3-CH), 4.16 (q, $^2J_{HH}$ = 7.2 Hz, 2H, CH_2), 1.35 (t, $^2J_{HH}$ = 7.2 Hz, 2H, CH_3),

^{13}C{^{1}H} NMR (DMSO-d_6, 100 MHz) δ (ppm) = 158.4 (s, 4-C), 142.5 (s, 2-C), 109.2 (s, 3-C), 52.2 (s, CH_2), 15.9 (s, CH_3).

HRMS (ESI): m/z calculated for $[C_7H_{11}N_2]^+$ $(M\text{-}Br)^+$ 123.09167, found 123.09159.

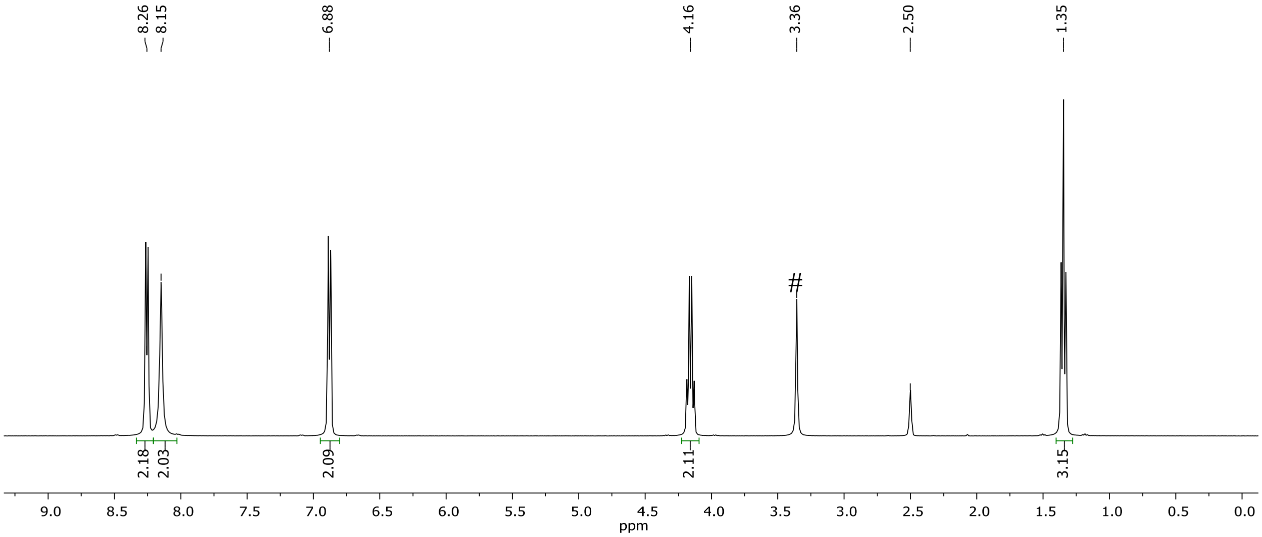

Figure S1: ^{1}H NMR spectrum (DMSO-d_6, 300 K, 400 MHz) of **1a**. # H_2O

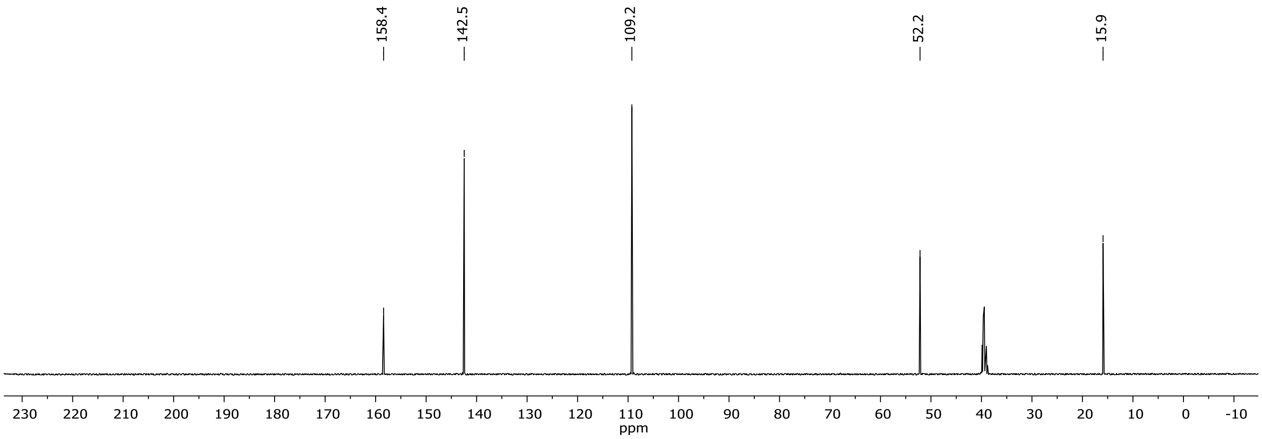

Figure S2: ^{13}C{^{1}H} NMR spectrum (DMSO-d_6, 300 K, 100 MHz) of **1a**.

Compound 1b

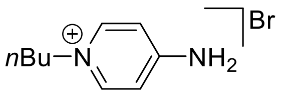

4-Aminopyridine (5.00 g, 53.1 mmol, 1 eq.) and 1-bromobutane (8.5 mL, 79.7 mmol, 1.5 eq.) were dissolved in MeCN (50 mL) and stirred for 16 h at 80 °C. The colourless solid was filtered off, washed with acetone (3 x 20 mL) and dried *in vacuo* at 120 °C for 16 h. Yield: 11.9 g (51.6 mmol, 97%).

^{1}H NMR (DMSO-d_6, 400 MHz) δ (ppm) = 8.26 (m, 2H, Ar-H), 8.18 (s, 2H, NH_2), 6.89 (m, 2H, Ar-H), 4.13 (t, $^3J_{HH}$ = 7.3 Hz, 2H, N-CH_2), 1.71 (tt, $^3J_{HH}$ = 7.3 Hz, $^3J_{HH}$ = 7.3 Hz, 2H, N-CH_2-CH_2), 1.22 (tq, $^3J_{HH}$ = 7.3 Hz, $^3J_{HH}$ = 7.3 Hz, 2H, N-$(CH_2)_2$-CH_2), 0.86 (t, $^3J_{HH}$ = 7.3 Hz, 3H, N-$(CH_2)_3$-CH_3).

^{13}C{^{1}H} NMR (DMSO-d_6, 100 MHz) δ (ppm) = 158.4 (s, 4-C), 142.7 (s, 2-C), 109.1 (s, 3-C), 56.5 (s, N-*C*H_2), 32.1 (s, N-CH_2-*C*H_2), 18.5 (s, N-$(CH_2)_2$-*C*H_2), 13.2 (s, N-$(CH_2)_3$-*C*H_3).

HRMS (ESI): m/z calculated for $[C_9H_{15}N_2]^+$ $(M\text{-}Br)^+$ 151.12298, found: 151.12326.

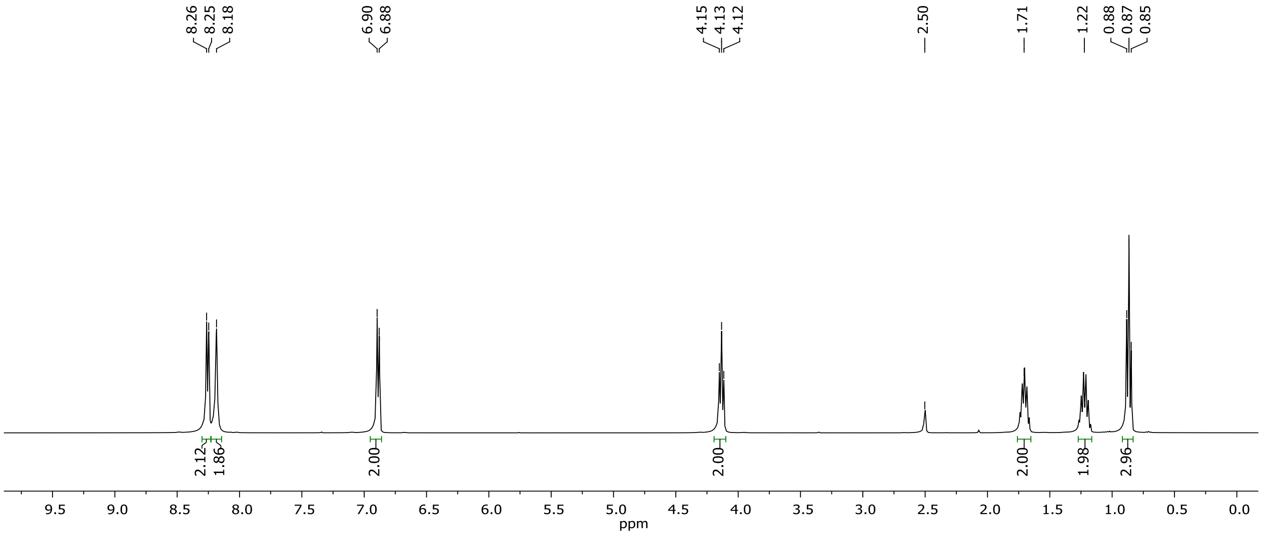

Figure S3: ^{1}H NMR spectrum (DMSO-d_6, 300 K, 400 MHz) of **1b**.

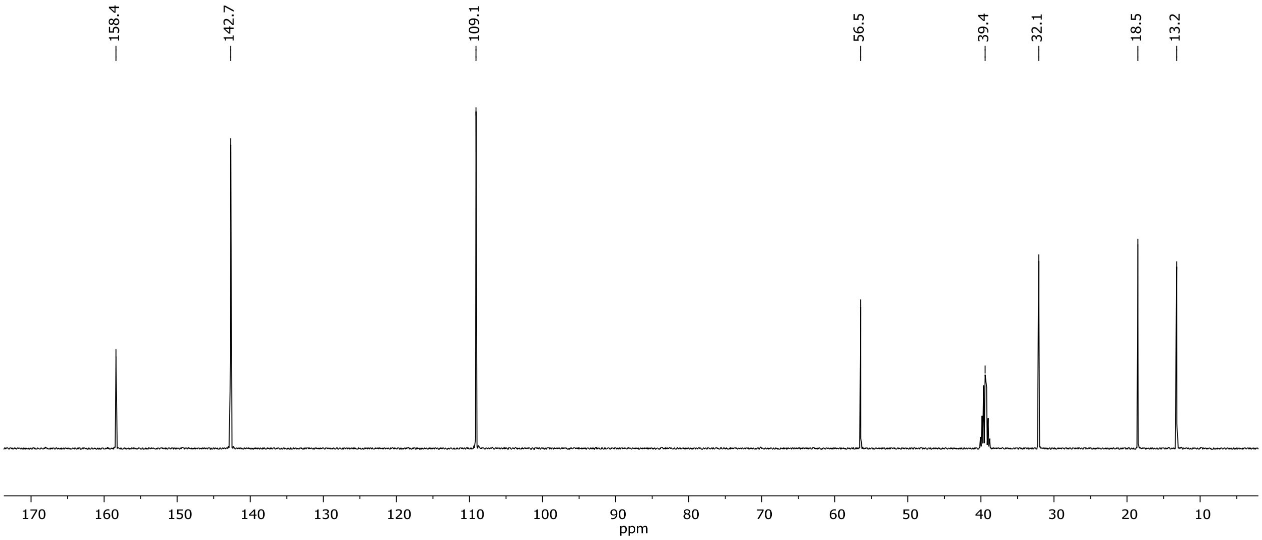

Figure S4: ^{13}C{^{1}H} NMR spectrum (DMSO-d_6, 300 K, 100 MHz) of **1b**.

Compound 3a

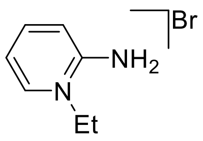

2-Aminopyridine (5.00 g, 53.1 mmol, 1 eq.) and bromoethane (5.9 mL, 79.7 mmol, 1.5 eq.) were dissolved in MeCN (50 mL) and heated at 80 °C for 48 h. The salt was precipitated out of the solution with Et_2O (100 mL), filtered off and washed with Et_2O. The colourless powder was dried *in vacuo* at 120 °C for 16 h. Yield: 9.93 g (48.9 mmol, 92%).

^{1}H NMR (DMSO-d_6, 400 MHz) δ (ppm) = 8.56 (br, 2H, NH_2), 8.16 (dd, $^3J_{HH}$ = 6.7 Hz, $^4J_{HH}$ = 1.7 Hz, 1H, 6-CH), 7.87 (ddd, $^3J_{HH}$ = 8.7 Hz, $^3J_{HH}$ = 7.0 Hz, $^4J_{HH}$ = 1.6 Hz, 1H, 4-CH), 7.16 (d, $^3J_{HH}$ = 8.9 Hz, 1H, 3-CH), 6.91 (ddd, $^3J_{HH}$ = 6.9 Hz, $^3J_{HH}$ = 6.9 Hz, $^4J_{HH}$ = 1.4 Hz, 1H, 5-CH), 4.24 (q, $^3J_{HH}$ = 7.2 Hz, 2H, CH_2), 1.29 (t, $^3J_{HH}$ = 7.2 Hz, 3H, CH_3).

^{13}C{^{1}H} NMR (DMSO-d_6, 100 MHz) δ (ppm) = 153.2 (s, 2-C), 142.0 (s, Ar-C), 139.4 (s, Ar-C), 114.6 (s, Ar-C), 113.0 (s, Ar-C), 48.3 (s, CH_2), 13.2 (s, CH_3).

HRMS (ESI): m/z calculated for $[C_7H_{11}N_2]^+$ $(M\text{-}Br)^+$ 123.09167, found 123.09224.

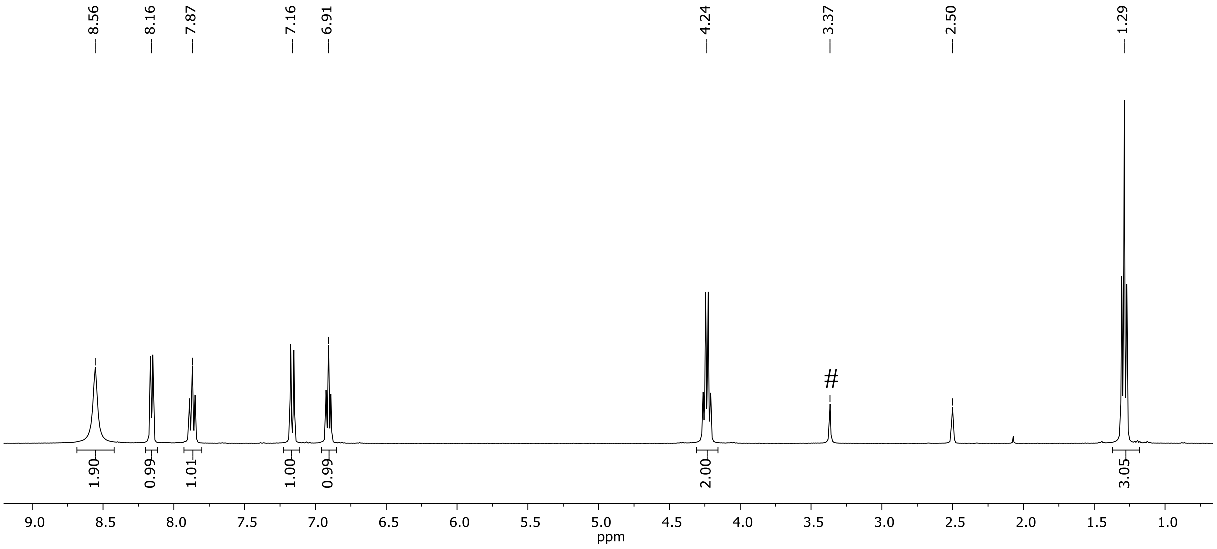

Figure S5: ^{1}H NMR spectrum (DMSO-d_6, 300 K, 400 MHz) of **3a**. # H_2O

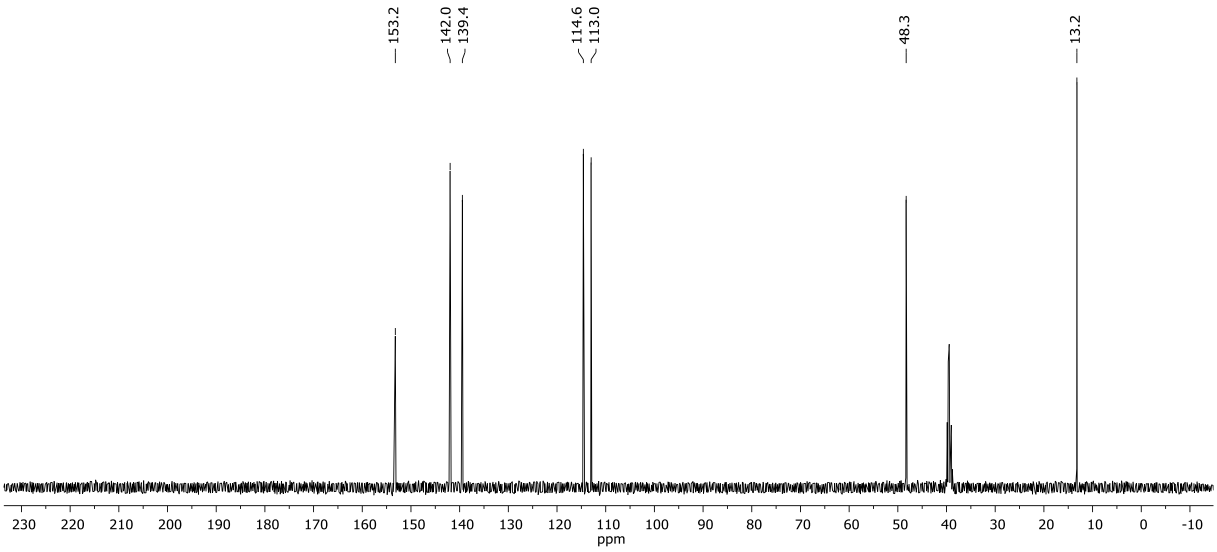

Figure S6: $^{13}C\{^1H\}$ NMR spectrum (DMSO-d_6, 300 K, 100 MHz) of **3a**.

Compound 3b

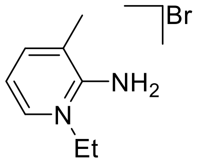

2-Amino-3-methylpyridine (35.0 mL, 347 mmol, 1 eq.) and bromoethane (28.3 mL, 382 mmol, 1.1 eq.) were dissolved in MeCN (100 mL) and heated at 80 °C for 48 h. All volatile compounds were removed *in vacuo* and the residue was recrystallized from CH_2Cl_2 to afford **3b** as white solid which was dried at 120 °C for 16 h *in vacuo*. Yield: 59.8 g (275 mmol, 79%).

^{1}H NMR (MeCN-d_3, 400 MHz) δ (ppm) = 7.78 (d, 1H, $^3J_{HH}$ = 6.7 Hz, 6-CH), 7.72 – 7.52 (m, 3H, 4-CH, NH_2), 6.82 (dd, $^3J_{HH}$ = 6.9 Hz, $^3J_{HH}$ = 6.9 Hz, 1H, 5-CH), 4.46 (q, $^3J_{HH}$ = 7.2 Hz, 2H, CH_2), 2.30 (s, 3H, Ar-CH_3), 1.40 (t, $^3J_{HH}$ = 7.2 Hz, 3H, CH_2CH_3).

^{13}C{^{1}H} NMR (DMSO-d_6, 100 MHz) δ (ppm) = 152.3 (s, 2-C), 141.0 (s, Ar-C), 137.4 (s, Ar-C), 123.2 (s, 3-C), 112.7 (s, Ar-C), 48.9 (s, CH_2), 17.4 (s, Ar-CH_3), 13.3 (s, CH_2CH_3).

HRMS (ESI): m/z calculated for $[C_8H_{12}N_2]^+$ $(M\text{-}Br)^+$ 137.1073, found 137.1075.

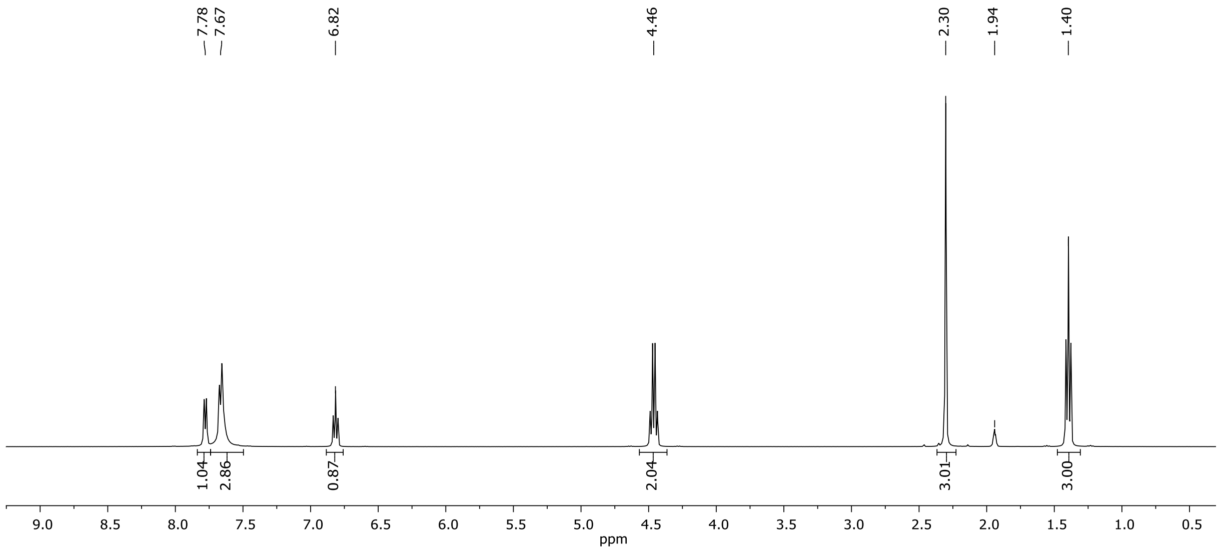

Figure S7: ^{1}H NMR spectrum (MeCN-d_3, 300 K, 400 MHz) of **3b**.

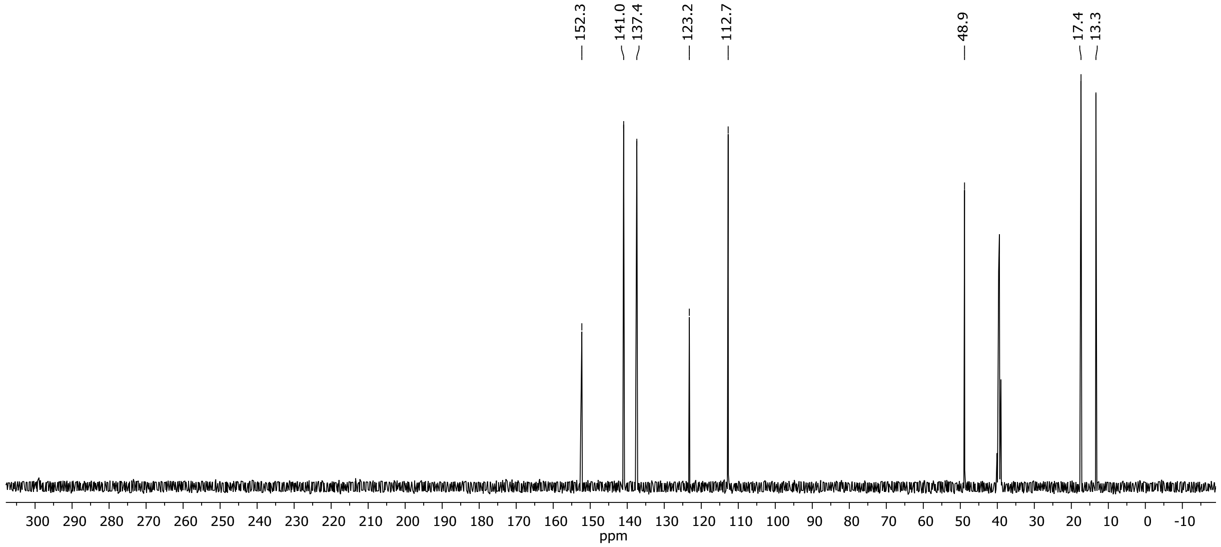

Figure S8: ^{13}C{^{1}H} NMR spectrum (DMSO-d_6, 300 K, 100 MHz) of **3b**.

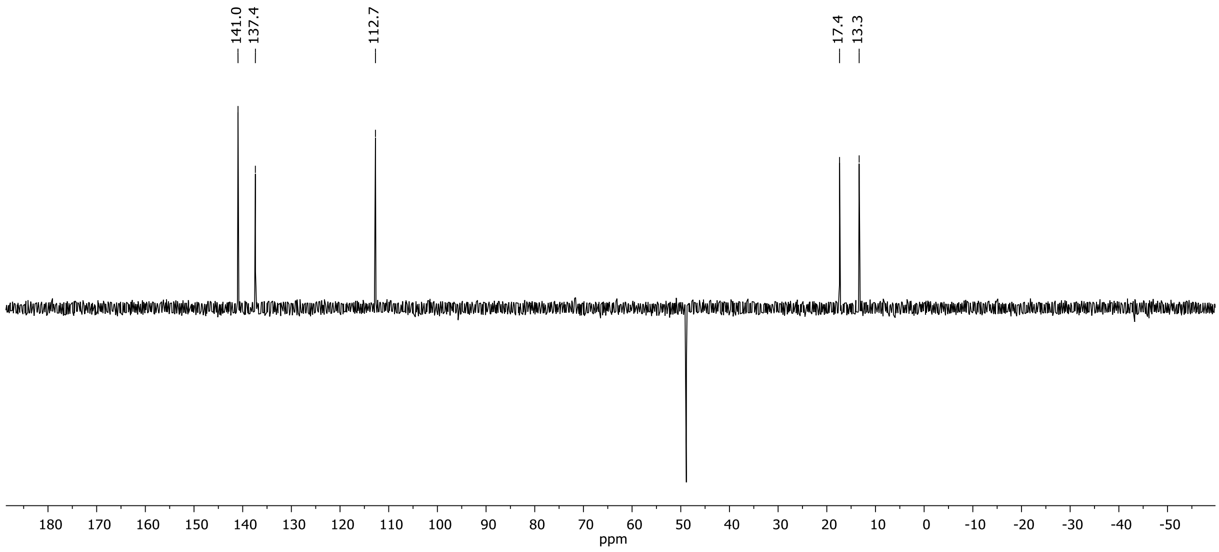

Figure S9: ^{13}C{^{1}H}Dept135 NMR spectrum (DMSO-d_6, 300 K, 100 MHz) of **3b**.

Synthesis of phosphines **2a-d** and **4a-d**

General Procedure a) using excess imine as base: The pyridinium salt (2 eq.) and KHMDS (2 eq.) were suspended in THF (10 mL*$mmol^{-1}$) and stirred for 16 h. $PClR_2$ (1 eq.) was added dropwise at room temperature. After 3 h all volatile compounds were removed *in vacuo* and the residue was extracted with *n*-hexane (3 x 20 mL) to give the corresponding phosphine.

General Procedure b) using KHMDS as base: Pyridinium salt **1a** (1 eq.), KHMDS (2 eq.) and $PClR_2$ (1 eq.) were suspended in THF (10 mL*$mmol^{-1}$). After 3 h all volatile compounds were removed *in vacuo* and the residue was extracted with *n*-hexane (3 x 20 mL) to give the corresponding phosphine.

Compound 2a

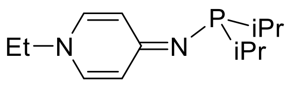

Compound **2a** was prepared according to the general procedure a) using the following: **1a** (524 mg, 2.58 mmol), KHMDS (515 mg, 2.58 mmol) and $PCl(iPr)_2$ (2 mL, 0.645 M in toluene, 1.29 mmol). Yield: 270 mg (1.13 mmol, 88%).

According to the general procedure b) the following was used: **1a** (262 mg, 1.29 mmol), KHMDS (515 mg, 2.58 mmol) and $PCl(iPr)_2$ (2 mL, 0.645 M in toluene, 1.29 mmol). Yield: 285 mg (1.20 mmol, 93%).

^{1}H NMR (benzene-d_6, 400 MHz) δ (ppm) = 6.72 (dd, $^3J_{HH}$ = 7.9 Hz, $^4J_{HH}$ = 2.5 Hz, 2H, 2-H), 5.83 (d, $^3J_{HH}$ = 7.6 Hz, 2H, 3-H), 2.37 (q, $^3J_{HH}$ = 7.3 Hz, 2H, CH_2), 2.09 (hept, $^3J_{HH}$ = 7.1 Hz, 2H, C*H*(CH_3)$_2$), 1.64 – 1.08 (m, 12H, CH(C*H*$_3$)$_2$), 0.44 (t, $^3J_{HH}$ = 7.3 Hz, 3H, CH_3).

^{13}C{^{1}H} NMR (benzene-d_6, 100 MHz) δ (ppm) = 166.6 (d, $^2J_{PC}$ = 17.6 Hz, 4-C), 135.1 (d, $^4J_{PC}$ = 3.1 Hz, 2-C), 116.3 (d, $^3J_{PC}$ = 23.1 Hz, 3-C), 49.6 (s, CH_2), 27.92 (d, *J* = 10.9 Hz), 19.67 (d, *J* = 19.3 Hz), 18.24 (d, *J* = 8.8 Hz), 15.3 (s, CH_3).

^{31}P NMR (benzene-d_6, 160 MHz) δ (ppm) = 56.5 (s).

CHN-Analysis: found (calculated) C 65.27 (65.52) H 9.77 (9.73) N 11.88 (11.76).

HRMS (ESI): m/z calculated for $[C_{13}H_{24}N_2P]^+$ $(M+H)^+$ 239.16716, found 239.16664.

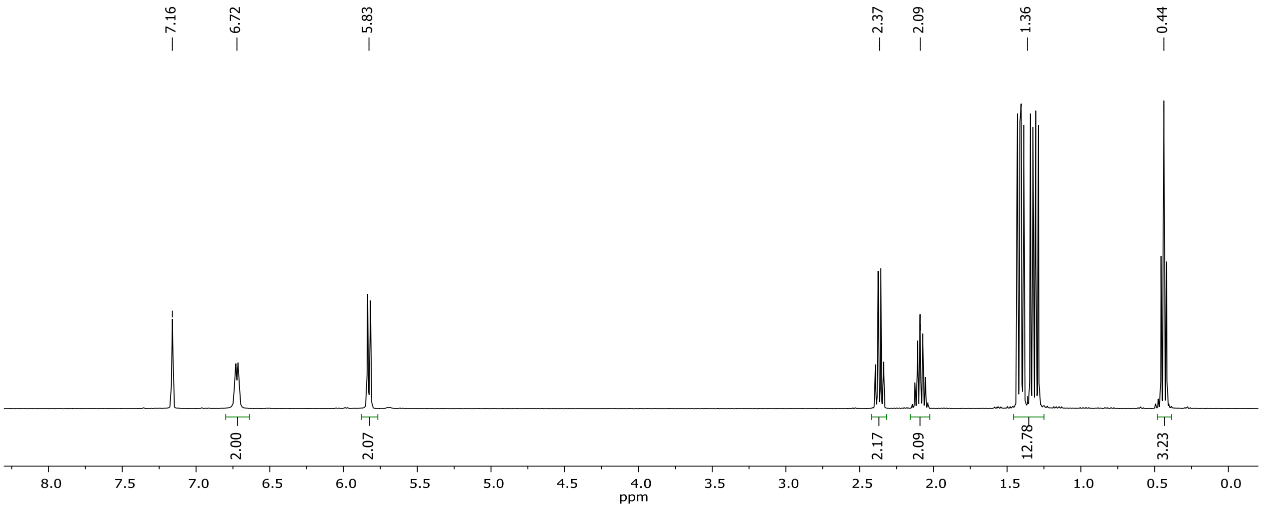

Figure S10: ^{1}H NMR spectrum (benzene-d_6, 300 K, 400 MHz) of **2a**.

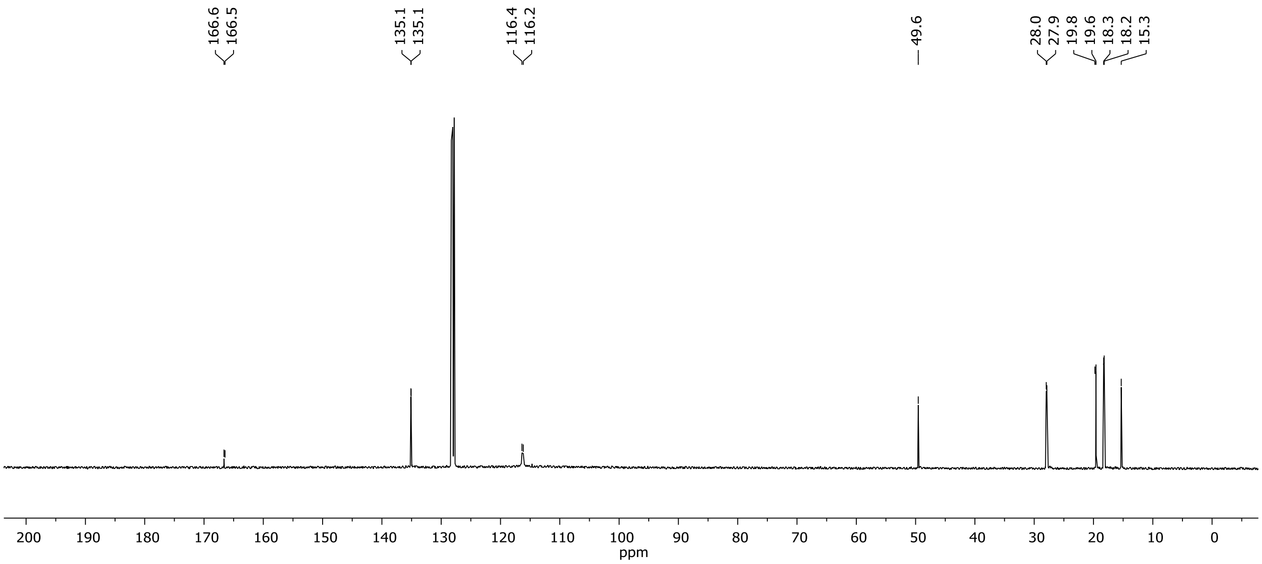

Figure S11: $^{13}C\{^{1}H\}$ NMR spectrum (benzene-d_6, 300 K, 100 MHz) of **2a**.

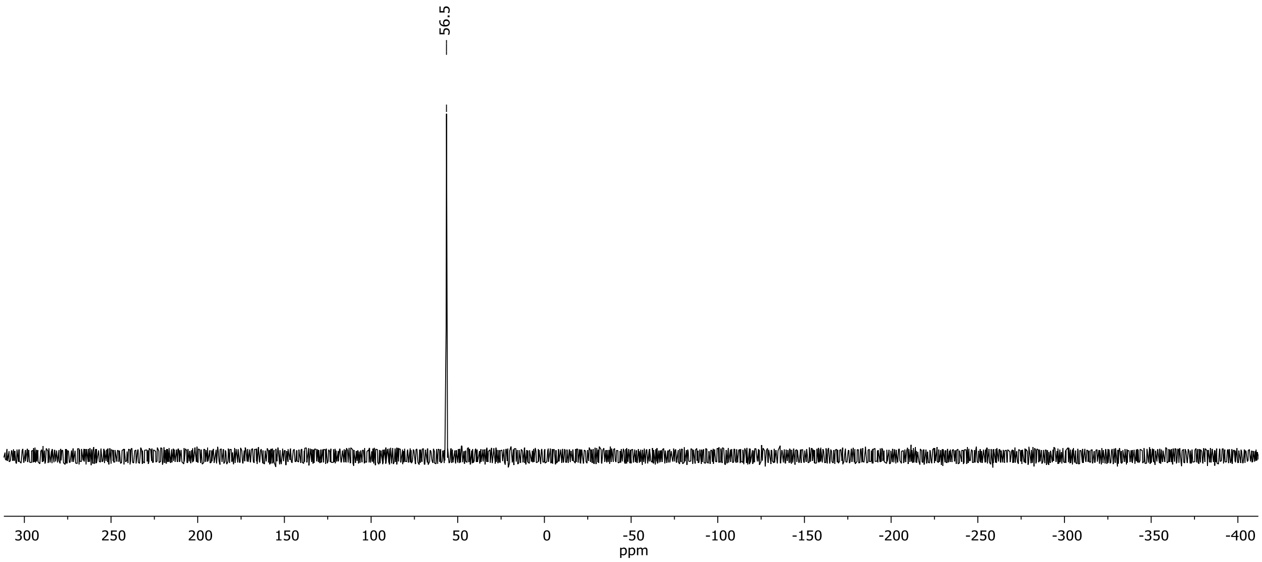

Figure S12: ^{31}P NMR spectrum (benzene-d_6, 300 K, 160 MHz) of **2a**.

Compound 2b

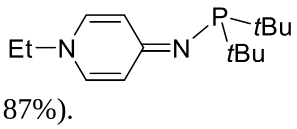

Compound **2b** was prepared according to the general procedure a) using the following: **1a** (1026 mg, 5.05 mmol), KHMDS (1007 mg, 5.05 mmol) and $PCl(tBu)_2$ (5 mL, 0.505 M in toluene, 2.53 mmol). Yield: 583 mg (2.19 mmol, 87%).

According the general procedure b) the following was used: **1a** (103 mg, 0.51 mmol), KHMDS (201 mg, 1.01 mmol) and $PCl(tBu)_2$ (1 mL, 0.505 M in toluene, 0.505 mmol). Yield: 113 mg (0.42 mmol, 84%).

^{1}H NMR (benzene-d_6, 400 MHz) δ (ppm) = 6.77 (m, 2H, 2-H), 5.84 (m, 2H, 3-H), 2.37 (q, $^{3}J_{HH}$ = 7.2 Hz, CH_2), 1.41 (d, $^{3}J_{PH}$ = 11.0 Hz, $C(CH_3)_3$), 0.44 (t, $^{3}J_{HH}$ = 7.3 Hz, CH_3).

^{13}C{^{1}H} NMR (benzene-d_6, 100 MHz) δ (ppm) = 166.5 (d, $^{2}J_{PC}$ = 19.8 Hz, 4-C), 135.2 (d, J = 3.0 Hz, Ar-C), 116.5 (d, J = 22.7 Hz, Ar-C), 49.5 (s, CH_2), 34.4 (d, $^{1}J_{PC}$ = 19.8 Hz, $C(CH_3)_3$), 28.9 (d, $^{2}J_{PC}$ = 14.9 Hz, $C(CH_3)_3$), 15.3 (s, CH_3)

^{31}P NMR (benzene-d_6, 160 MHz) δ (ppm) = 68.9 (m).

HRMS (ESI): m/z calculated for $[C_{15}H_{28}N_2P]^+$ $(M+H)^+$ 267.19846, found 267.19858.

CHN-Analysis: found (calculated) C 67.60 (67.64) H 10.59 (10.22) N 10.53 (10.52).

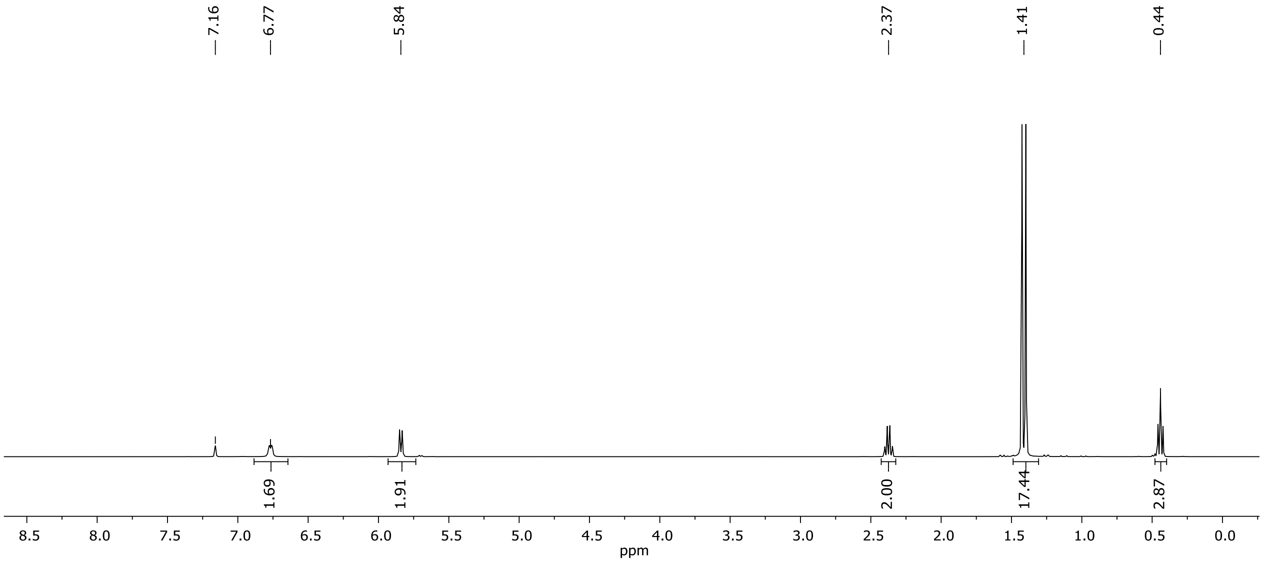

Figure S13: ^{1}H NMR spectrum (benzene-d_6, 300 K, 400 MHz) of **2b**.

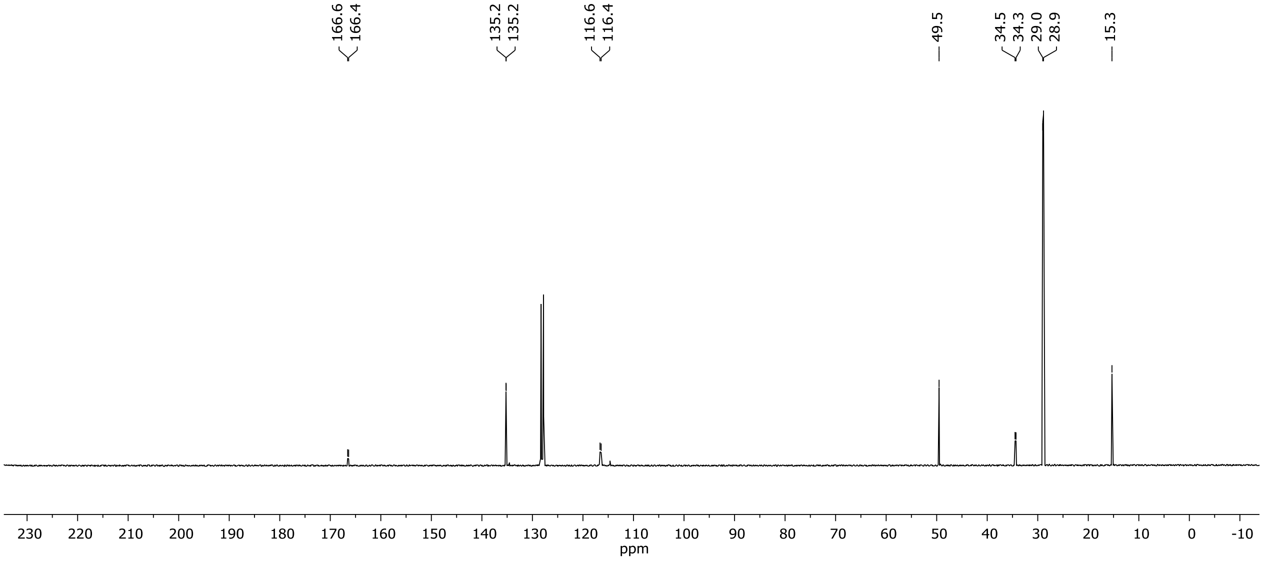

Figure S14: ^{13}C{^{1}H} NMR spectrum (benzene-d_6, 300 K, 100 MHz) of **2b**.

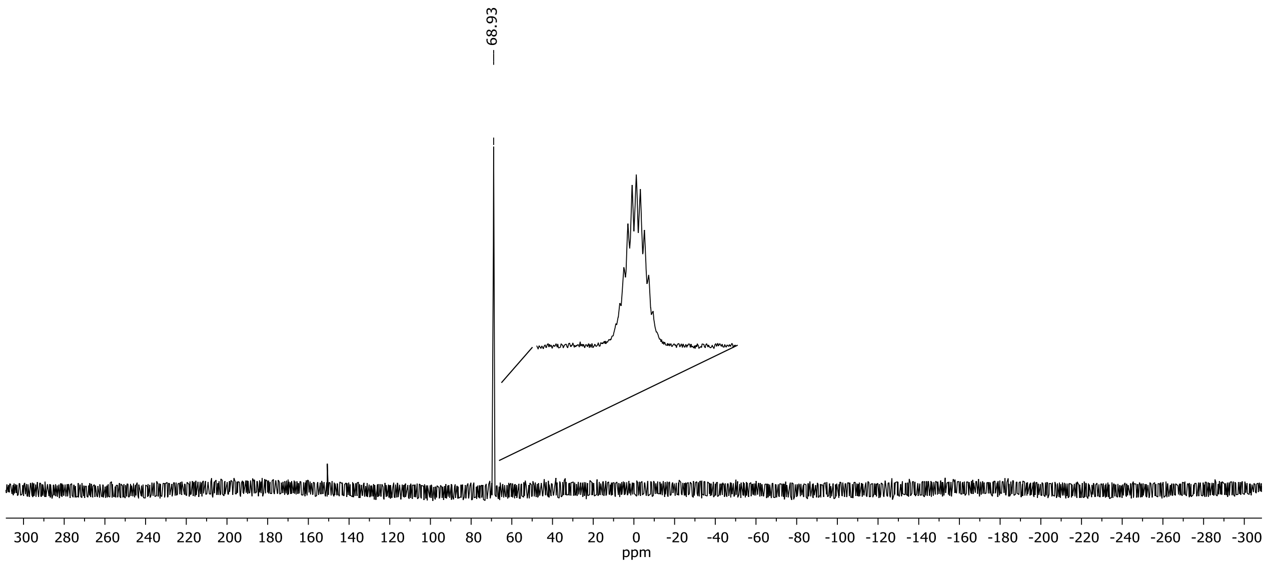

Figure S15: ^{31}P NMR spectrum (benzene-d_6, 300 K, 160 MHz) of **2b**.

Compound 2c

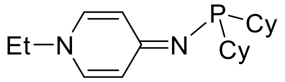

Compound **2c** was prepared according to the general procedure a) using the following: **1a** (699 mg, 3.44 mmol), KHMDS (686 mg, 3.44 mmol) and $PCl(Cy)_2$ (20 mL, 0.086 M in toluene, 1.72 mmol). Yield: 464 mg (1.46 mmol, 85%).

According the general procedure b) the following was used: **1a** (171 mg, 0.844 mmol), KHMDS (337 mg, 1.69 mmol) and $PCl(Cy)_2$ (2 mL, 0.422 M in toluene, 0.844 mmol). Yield: 231 mg (0.726 mmol, 86%).

^{1}H NMR (benzene-d_6, 400 MHz) δ (ppm) = 6.74 (m, 2H, 2-H), 5.83 (d, 2H, $^3J_{HH}$ = 7.5 Hz, 3-H), 2.37 (q, $^3J_{HH}$ = 7.3 Hz, CH_2), 2.28 – 0.76 (m, 22H, Cy), 0.44 (t, $^3J_{HH}$ = 7.3 Hz, CH_3).

^{13}C{^{1}H} NMR (benzene-d_6, 100 MHz) δ (ppm) = 166.4 (d, $^2J_{PC}$ = 17.7 Hz, 4-C), 135.0 (d, $^4J_{PC}$ = 3.1 Hz, 2-C), 116.3 (d, $^3J_{PC}$ = 23.8 Hz, 3-C), 49.5 (s, CH_2), 38.1 (d, J_{PC} = 11.0 Hz, Cy), 30.1 (d, J_{PC} = 17.5 Hz, Cy), 28.4 (d, J_{PC} = 7.8 Hz, Cy), 27.8 (m, Cy), 27.4 (s, Cy), 14.2 (s, CH_3).

^{31}P NMR (benzene-d_6, 160 MHz) δ (ppm) = 49.6 (s).

HRMS (ESI): m/z calculated for $[C_{19}H_{32}N_2P]^+$ $(M+H)^+$ 319.22976, found 319.23007.

CHN-Analysis: found (calculated) C 71.45 (71.66) H 9.81 (9.81) N 8.89 (8.80).

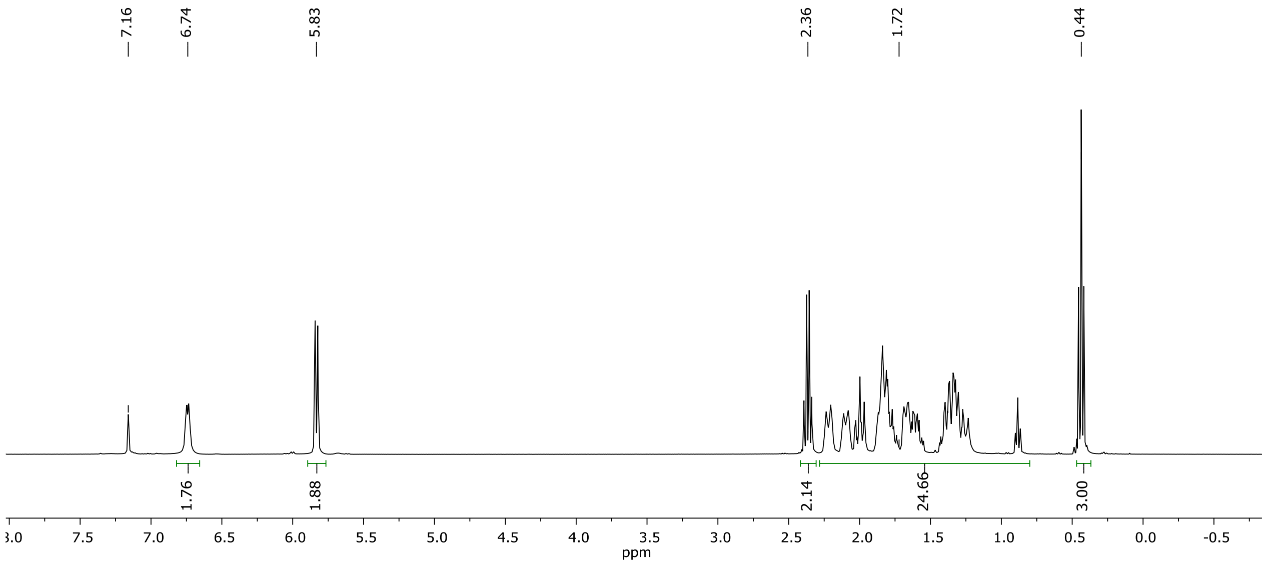

Figure S16: ^{1}H NMR spectrum (benzene-d_6, 300 K, 400 MHz) of **2c**.

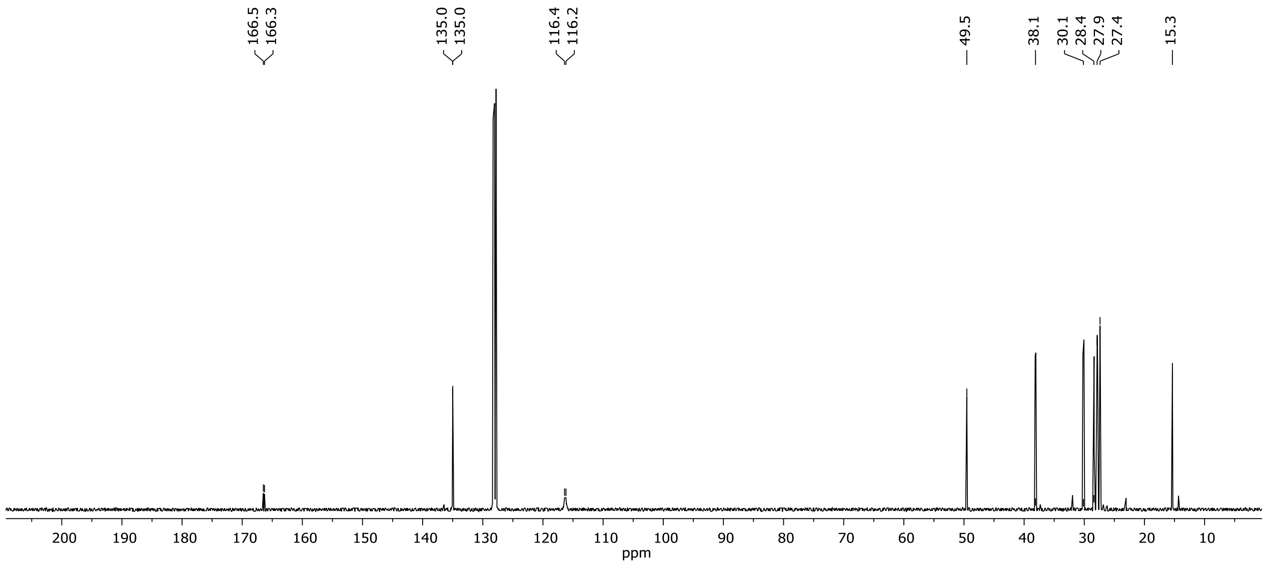

Figure S17: ^{13}C{^{1}H} NMR spectrum (benzene-d_6, 300 K, 100 MHz) of **2c.**

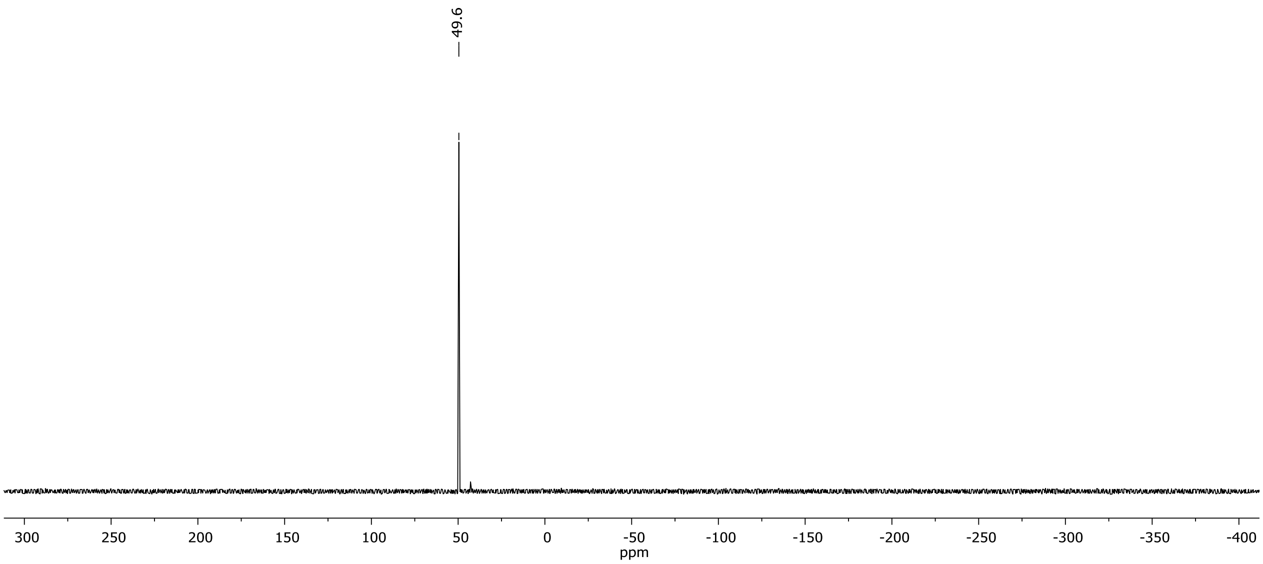

Figure S18: ^{31}P NMR spectrum (benzene-d_6, 300 K, 160 MHz) of **2c**.

Compound 2d

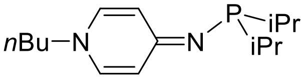

Compound **2d** was prepared according to the general procedure a) using the following: **1b** (596 mg, 2.58 mmol), KHMDS (515 mg, 2.58 mmol) and $PCl(iPr)_2$ (2 mL, 0.645 M in toluene, 1.29 mmol).

Yield: 320 mg (1.20 mmol, 93%).

^{1}H NMR (benzene-d_6, 400 MHz) δ (ppm) = 6.74 (m, 2H, 2-H), 5.88 (d, $^3J_{HH}$ = 7.6 Hz, 2H, 3-H), 2.43 (t, $^3J_{HH}$ = 7.0 Hz, 2H, NCH_2), 2.09 (hept, $^3J_{HH}$ = 7.0 Hz, 2H, C*H* $(CH_3)_2$), 1.54 – 1.19 (m, 12H, $CH(CH_3)_2$), 0.86 (m, 2H, NCH_2CH_2), 0.75 (m, 2H, $N(CH_2)_2CH_2$), 0.58 (t, $^3J_{HH}$ = 7.1 Hz, 3H, $N(CH_2)_3CH_3$).

^{13}C{^{1}H} NMR (benzene-d_6, 100 MHz) δ (ppm) = 166.5 (d, $^2J_{PC}$ = 17.5 Hz, 4-C), 135.5 (2-C), 116.1 (3-C), 54.6 (s, N-CH_2), 32.4 (s, N-CH_2-CH_2), 28.0 (d, $^1J_{PC}$ = 11.0 Hz, *C*H$(CH_3)_2$), 19.6 (m, CH$(CH_3)_2$), 18.3 (N-$(CH_2)_2$-CH_2), 13.5 (s, N-$(CH_2)_3$-CH_3).

^{31}P NMR (benzene-d_6, 160 MHz) δ (ppm) = 56.4 (s).

CHN-Analysis: found (calculated) C 67.21 (67.64) H 10.24 (10.22) N 11.53 (11.52).

HRMS (ESI): m/z calculated for $[C_{15}H_{28}N_2P]^+$ $(M+H)^+$ 267.19846, found 267.19939.

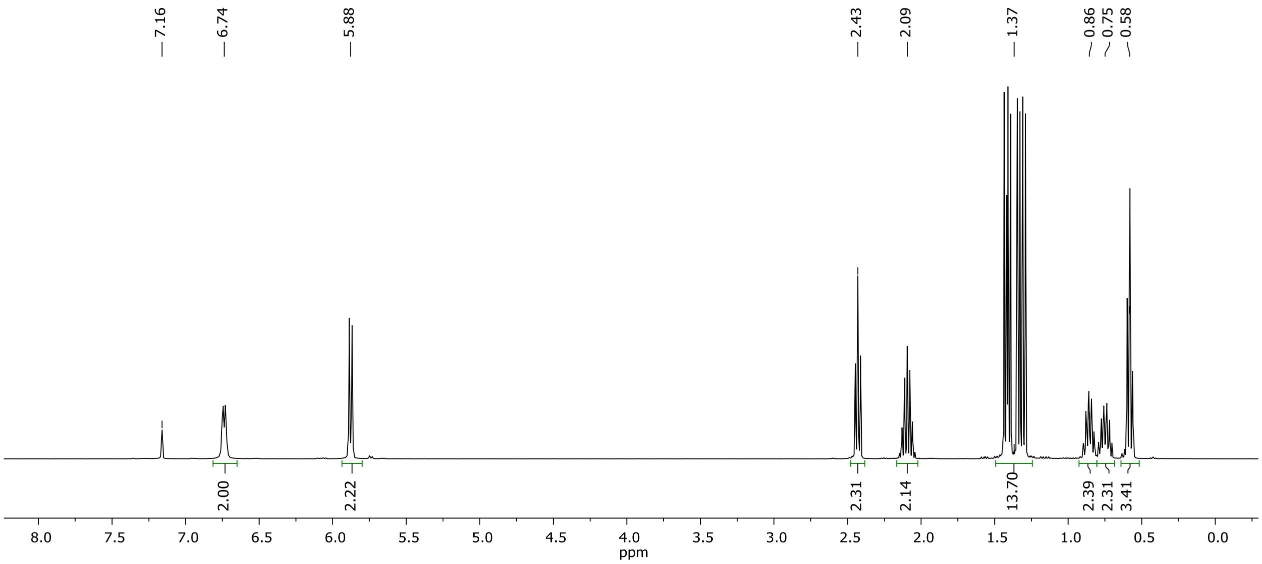

Figure S19: ^{1}H NMR spectrum (benzene-d_6, 300 K, 400 MHz) of **2d**.

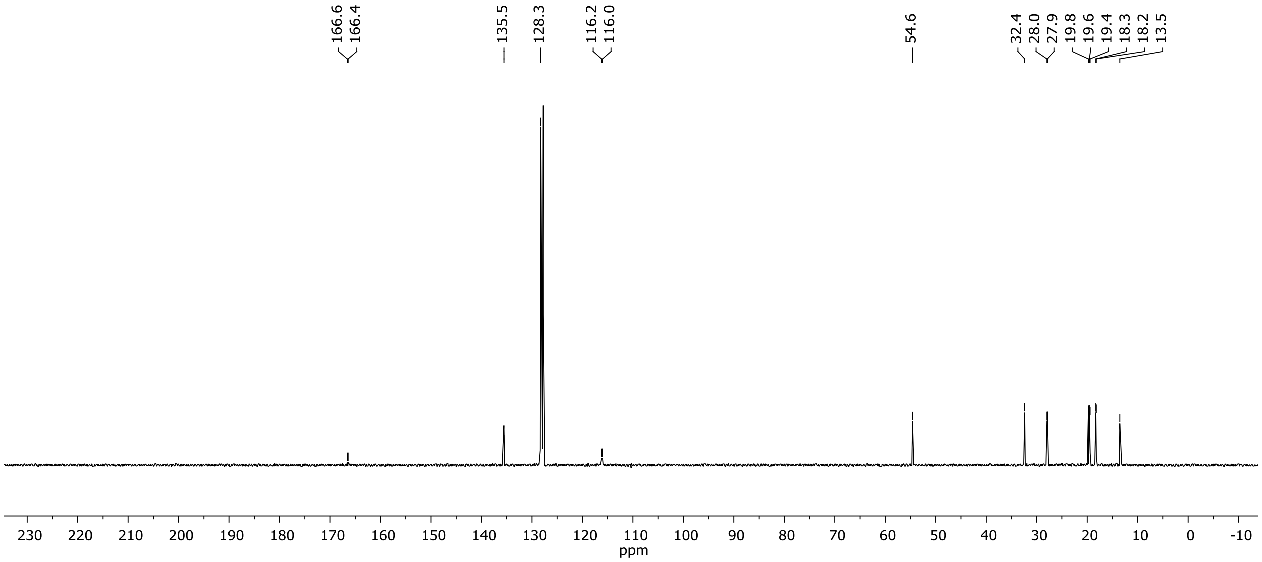

Figure S20: ^{13}C{^{1}H} NMR spectrum (benzene-d_6, 300 K, 100 MHz) of **2d**.

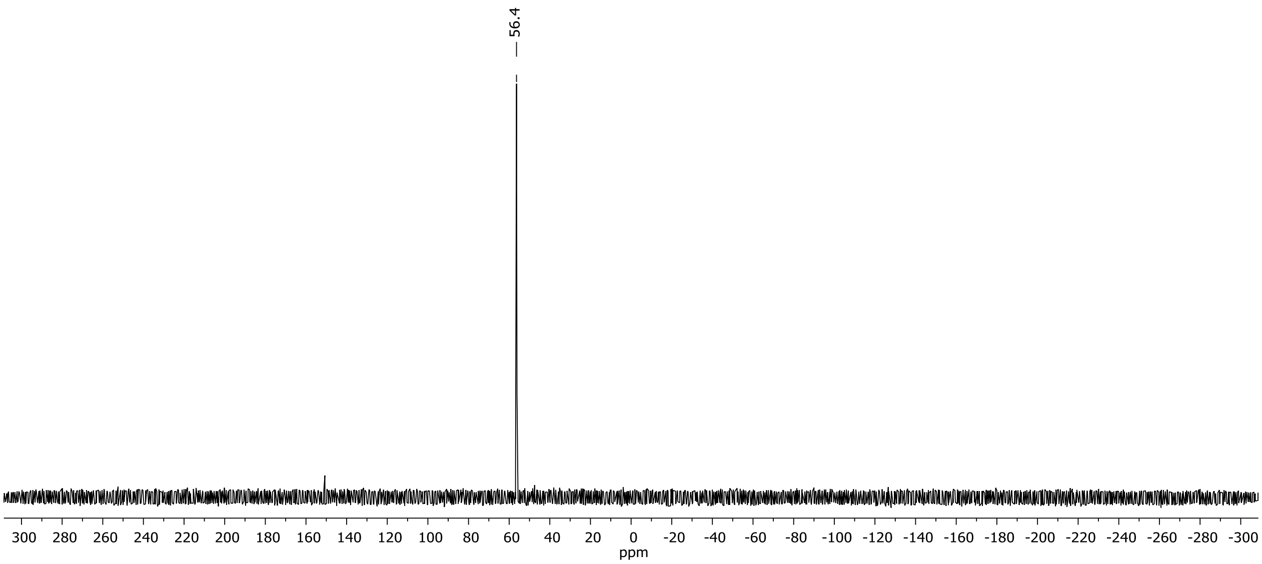

Figure S21: ^{31}P NMR spectrum (benzene-d_6, 300 K, 160 MHz) of **2d**.

Compound 4a

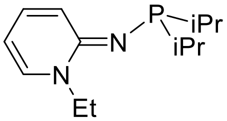

Compound **4a** was prepared according to the general procedure a) using the following: **3a** (524 mg, 2.58 mmol), KHMDS (515 mg, 2.58 mmol) and PCl(iPr)$_2$ (2 mL, 0.645 M in toluene, 1.29 mmol).

Yield: 296 mg (1.24 mmol, 96%).

^{1}H NMR (benzene-d_6, 400 MHz) δ (ppm) = 7.46 (m, 1H, Ar-H), 6.46 (ddd, $^3J_{HH}$ = 9.5 Hz, $^3J_{HH}$ = 6.3 Hz, $^4J_{HH}$ = 2.0 Hz, 1H, Ar-H), 6.25 (dd, $^3J_{HH}$ = 6.9 Hz, $^4J_{HH}$ = 1.9 Hz, 1H, Ar-H), 5.28 (ddd, $^3J_{HH}$ = 6.6 Hz, $^3J_{HH}$ = 6.6 Hz, $^4J_{HH}$ = 1.4 Hz, 1H, Ar-H), 3.56 (q, $^3J_{HH}$ = 7.1 Hz, 2H, CH$_2$), 1.91 (hept, $^3J_{HH}$ = 7.1 Hz, 2H, C*H*(CH$_3$)$_2$), 1.23 (m, 12H, CH(C*H*$_3$)$_2$), 0.99 (t, $^3J_{HH}$ = 7.1 Hz, 3H, CH$_3$).

^{13}C{^{1}H} NMR (benzene-d_6, 100 MHz) δ (ppm) = 158.5 (d, $^2J_{PC}$ = 25.2 Hz, 2-C), 137.3 (s, 6-C), 134.1 (d, $^4J_{PC}$ = 4.0 Hz, 4-C), 118.5 (d, $^3J_{PC}$ = 30.9 Hz, 3-C), 102.4 (d, $^5J_{PC}$ = 1.6 Hz, 5-C), 45.3 (s, CH$_2$), 27.6 (d, J = 11.2 Hz), 19.3 (d, J = 20.1 Hz), 17.7 (d, J = 8.3 Hz), 13.8 (s, CH$_3$).

^{31}P NMR (benzene-d_6, 160 MHz) δ (ppm) = 57.9 (s).

HRMS (ESI): m/z calculated for [C$_{13}$H$_{24}$N$_2$P]$^+$ (M+H)$^+$ 239.16716, found 239.16674.

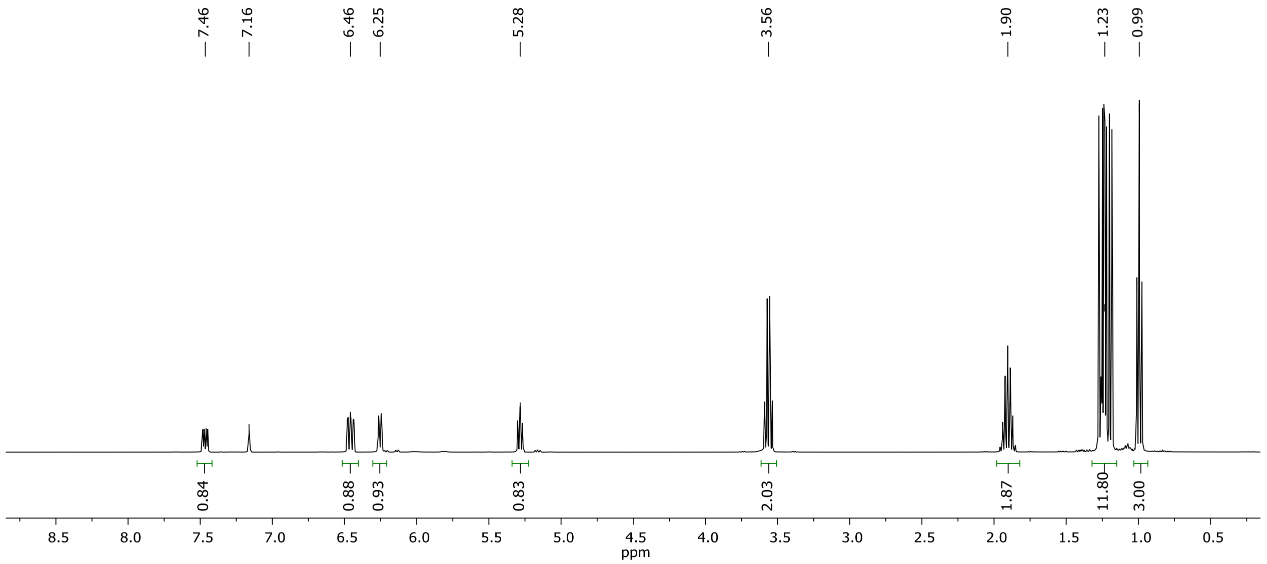

Figure S22: ^{1}H NMR spectrum (benzene-d_6, 300 K, 400 MHz) of **4a**.

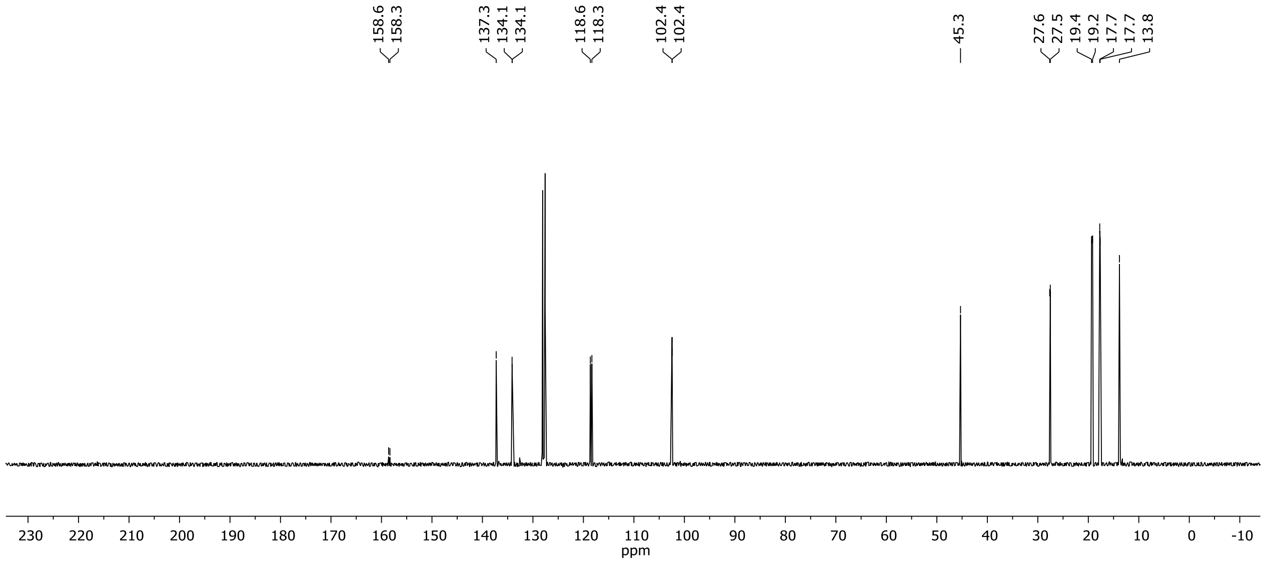

Figure S23: ^{13}C{^{1}H} NMR spectrum (benzene-d_6, 300 K, 100 MHz) of **4a**.

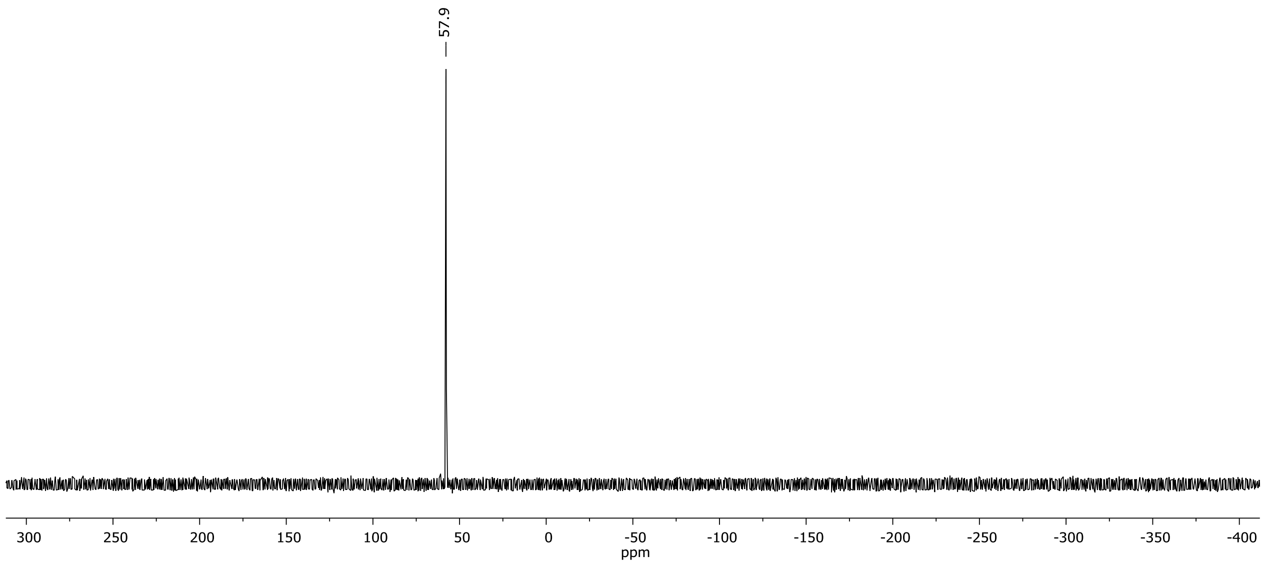

Figure S24: ^{31}P NMR spectrum (benzene-d_6, 300 K, 160 MHz) of **4a**.

Compound 4b

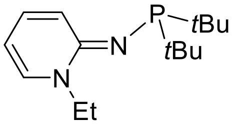

Compound **4b** was prepared according to the general procedure a) using the following: **3a** (1026 mg, 5.05 mmol), KHMDS (1007 mg, 5.05 mmol) and PCl(*t*Bu)$_2$ (5 mL, 0.505 M in toluene, 2.53 mmol). Yield: 611 mg (2.30 mmol, 91%).

^{1}H NMR (benzene-d_6, 400 MHz) δ (ppm) = 7.56 (dddd, $^3J_{HH}$ = 9.4 Hz, J = 4.6 Hz, J = 1.4 Hz, J = 0.7 Hz, 1H, 3-H), 6.47 (ddd, $^3J_{HH}$ = 9.5 Hz, $^3J_{HH}$ = 6.3 Hz, $^4J_{HH}$ = 2.0 Hz, 1H, 4-H), 6.25 (dddd, $^3J_{HH}$ = 6.9 Hz, J = 1.8 Hz, J = 0.8 Hz, J = 0.8 Hz, 1H, 6-H), 5.28 (ddd, $^3J_{HH}$ = 6.8 Hz, $^3J_{HH}$ = 6.3 Hz, $^4J_{HH}$ = 1.4 Hz, 1H, 5-H), 3.62 (q, $^3J_{HH}$ = 7.1 Hz, 2H, CH_2), 1.29 (d, $^3J_{PH}$ = 11.0 Hz, 18H, $C(CH_3)_3$), 1.02 (t, $^3J_{HH}$ = 7.1 Hz, 3H, CH_3).

^{13}C{^{1}H} NMR (benzene-d_6, 100 MHz) δ (ppm) = 158.6 (d, $^2J_{PC}$ = 26.8 Hz, 2-C), 137.4 (s, Ar-C), 134.3 (s, Ar-C), 119.1 (d, J_{PC} = 30.7 Hz, Ar-C), 102.6 (s, Ar-C), 45.4 (s, CH_2), 34.3 (d, $^1J_{PC}$ = 20.2 Hz, $\mathit{C}(CH_3)_3$), 28.8 (d, $^2J_{PC}$ = 15.1 Hz, $C(\mathit{C}H_3)_3$), 14.4 (s, CH_3).

^{31}P NMR (benzene-d_6, 160 MHz) δ (ppm) = 71.5 (m).

HRMS (ESI): m/z calculated for $[C_{15}H_{28}N_2P]^+$ $(M+H)^+$ 267.19846, found 267.19836.

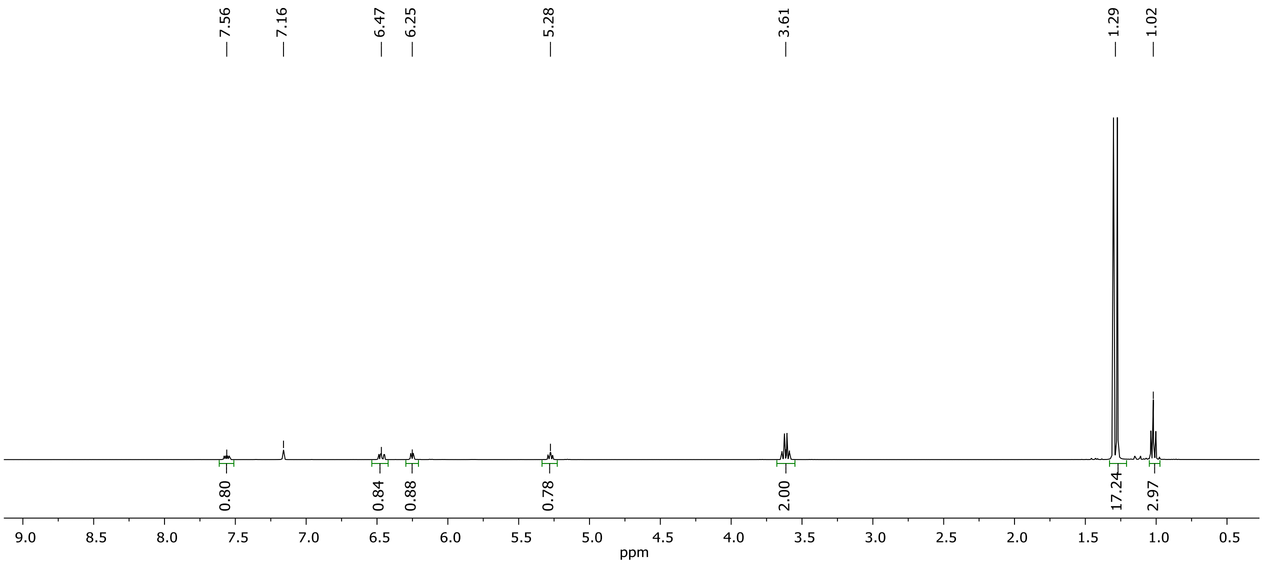

Figure S25: ^{1}H NMR spectrum (benzene-d_6, 300 K, 400 MHz) of **4b**.

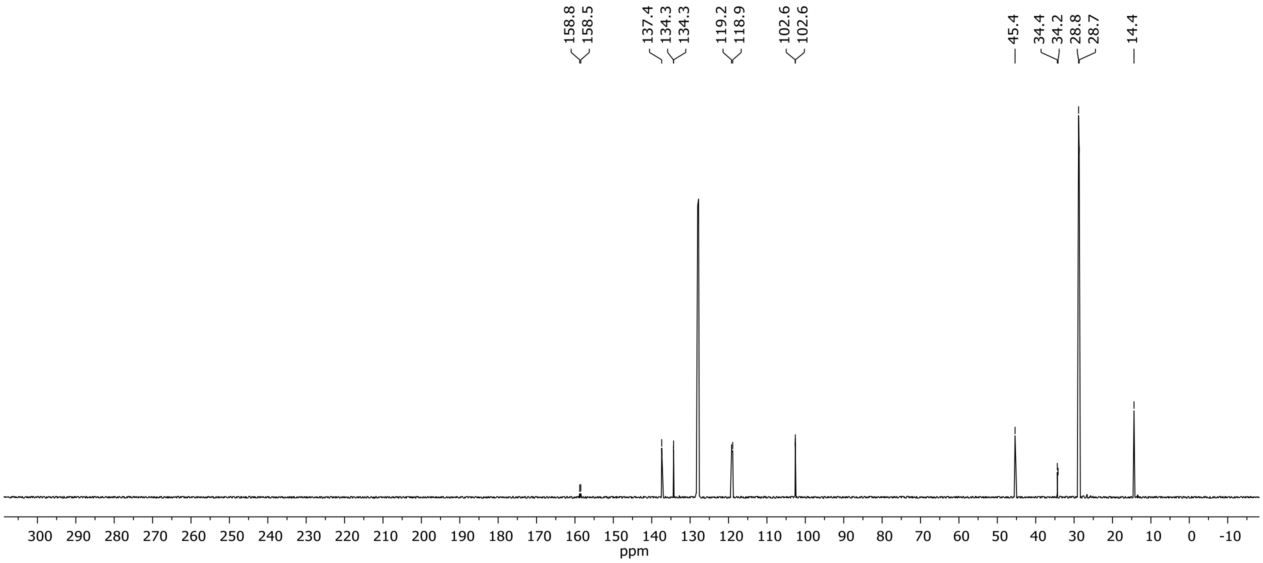

Figure S26: ^{13}C{^{1}H} NMR spectrum (benzene-d_6, 300 K, 100 MHz) of **4b**.

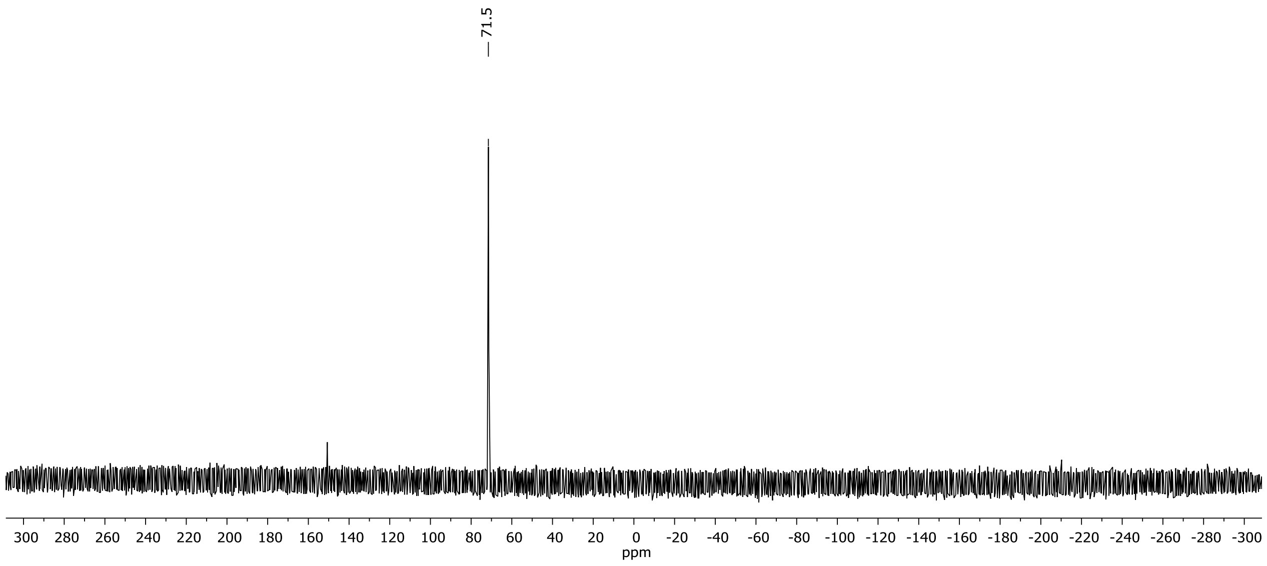

Figure S27: ^{31}P NMR spectrum (benzene-d_6, 300 K, 160 MHz) of **4b**.

Compound 4c

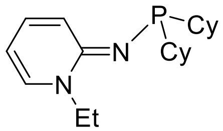

Compound **4c** was prepared according to the general procedure a) using the following: **3a** (857 mg, 4.22 mmol), KHMDS (842 mg, 4.22 mmol) and PCl(Cy)$_2$ (5 mL, 0.422 M in toluene, 2.11 mmol). Yield: 593 mg (1.86 mmol, 88%).

^{1}H NMR (benzene-d_6, 400 MHz) δ (ppm) = 7.50 (dddd, $^3J_{HH}$ = 9.4 Hz, J = 4.4 Hz, J = 1.0 Hz, J = 1.0 Hz, 1H, 3-H), 6.48 (ddd, $^3J_{HH}$ = 9.4 Hz, $^3J_{HH}$ = 6.3 Hz, $^4J_{HH}$ = 2.0 Hz, 1H, 4-H), 6.25 (dddd, $^3J_{HH}$ = 6.9 Hz, J = 1.8 Hz, J = 0.8 Hz, J = 0.8 Hz, 1H, 6-H), 5.29 (ddd, $^3J_{HH}$ = 6.6 Hz, $^3J_{HH}$ = 6.6 Hz, $^4J_{HH}$ = 1.4 Hz, 1H, 5-H), 3.59 (q, $^3J_{HH}$ = 7.0 Hz, 2H, CH$_2$), 2.20 – 1.12 (m, 22H, Cy), 1.02 (t, $^3J_{HH}$ = 7.1 Hz, 3H, CH$_3$).

^{13}C{^{1}H} NMR (benzene-d_6, 100 MHz) δ (ppm) = 158.6 (d, $^2J_{PC}$ = 25.8 Hz, 2-C), 137.5 (s, Ar-C), 134.2 (d, J_{PC} = 4.0 Hz, Ar-C), 118.7 (d, J_{PC} = 31.6 Hz, Ar-C), 102.6 (s, Ar-C), 45.4 (s, CH$_2$), 37.9 (d, J_{PC} = 11.5 Hz, cy), 29.9 (d, J_{PC} = 18.2 Hz, Cy), 28.1 (d, J_{PC} = 7.4 Hz, Cy), 27.8 (d, J_{PC} = 7.1 Hz, Cy), 27.7 (s, Cy), 14.2 (s, CH$_3$).

^{31}P NMR (benzene-d_6, 160 MHz) δ (ppm) = 51.5 (s).

HRMS (ESI): m/z calculated for $[C_{19}H_{32}N_2P]^+$ $(M+H)^+$ 319.22976, found 319.22958.

CHN-Analysis: found (calculated) C 71.42 (71.66) H 10.04 (9.81) N 8.56 (8.80).

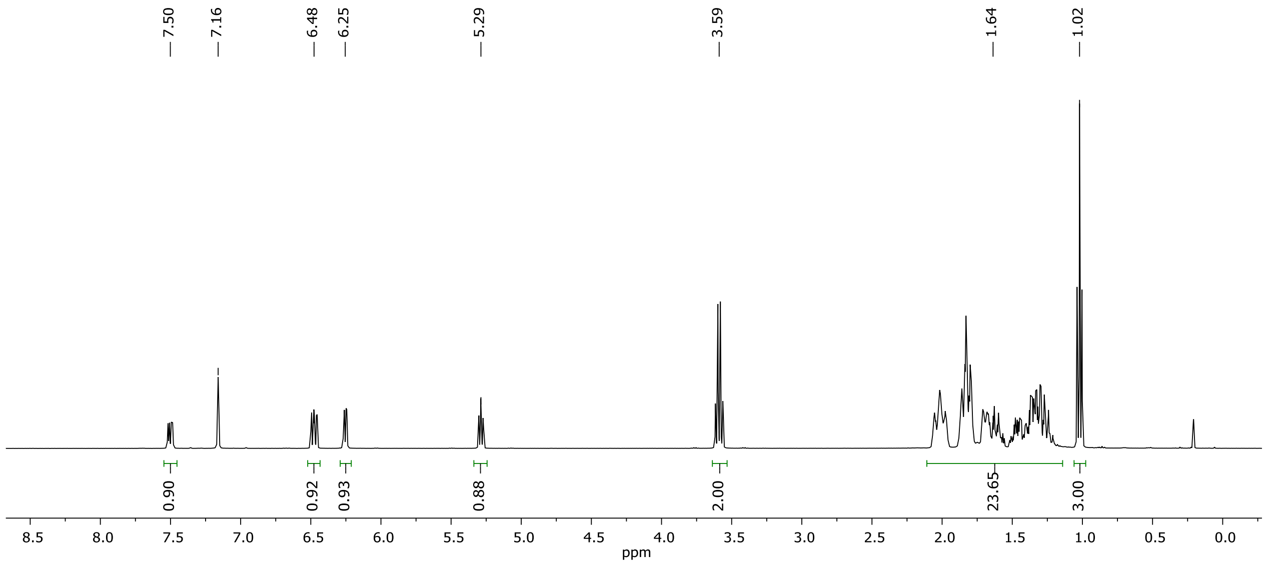

Figure S28: ^{1}H NMR spectrum (benzene-d_6, 300 K, 400 MHz) of **4c**.

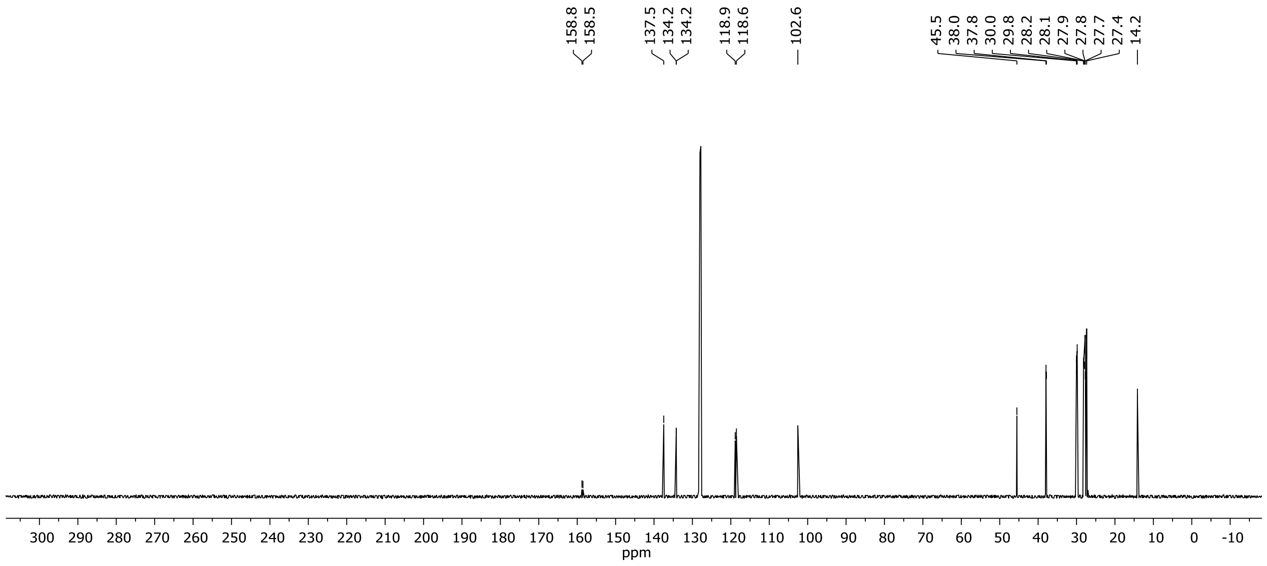

Figure S29: ^{13}C{^{1}H} NMR spectrum (benzene-d_6, 300 K, 100 MHz) of **4c.**

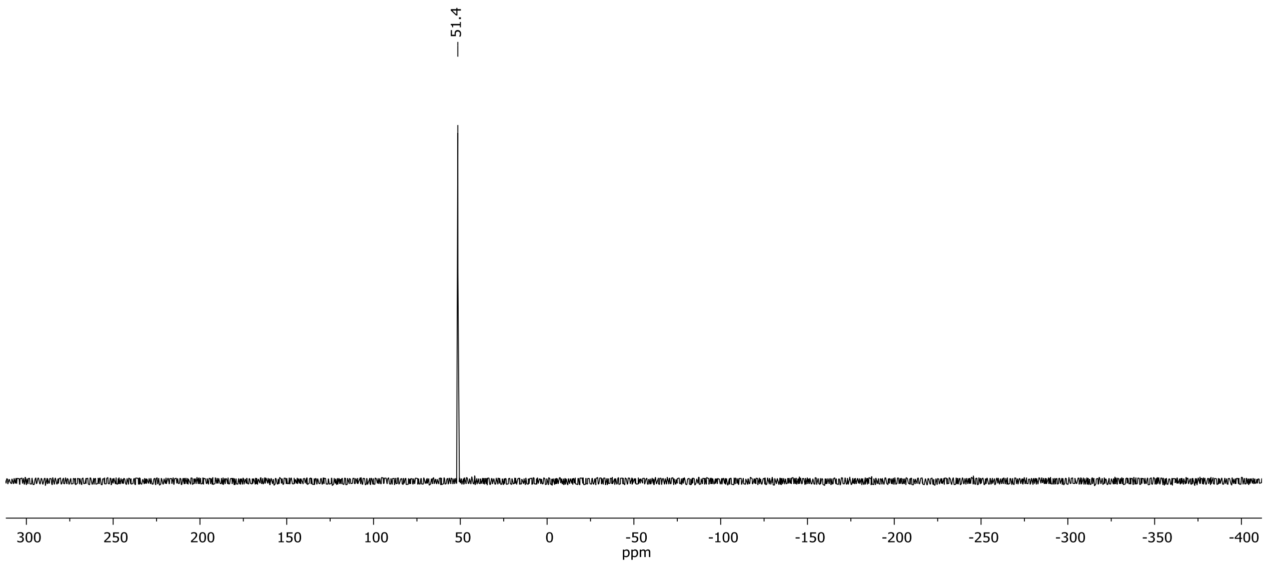

Figure S30: ^{31}P NMR spectrum (benzene-d_6, 300 K, 160 MHz) of **4c**.

Compound 4d

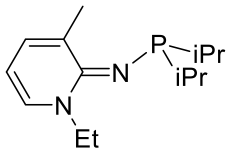

Compound **4d** was prepared according to the general procedure a) using the following: **3b** (560 mg, 2.58 mmol) KHMDS (515 mg, 2.58 mmol) and PCl(iPr)$_2$ (2 mL, 0.645 M in toluene, 1.29 mmol). Yield: 324 mg (1.17 mmol, 99%).

^{1}H NMR (benzene-d_6, 400 MHz) δ (ppm) = 6.42 (ddd, $^3J_{HH}$ = 6.6 Hz, $^4J_{HH}$ = 2.1 Hz, $^4J_{HH}$ = 1.1 Hz, 1H, Ar-H), 6.37 (dd, $^3J_{HH}$ = 6.4 Hz, $^4J_{HH}$ = 1.8 Hz, 1H, Ar-H), 5.40 (dd, $^3J_{HH}$ = 6.7 Hz, $^3J_{HH}$ = 6.7 Hz, 1H, 5-H), 3.69 (q, $^3J_{HH}$ = 7.1 Hz, 2H, CH$_2$), 2.51 (d, $^4J_{HH}$ = 2.7 Hz, 3H, CH$_3$), 1.80 (heptd, $^3J_{HH}$ = 7.0 Hz, $^2J_{PH}$ = 1.6 Hz, 2H, C*H*(CH$_3$)$_2$), 1.21 (m, 12H, CH(C*H*$_3$)$_2$, 1.03 (t, $^3J_{HH}$ = 7.0 Hz, 3H, CH$_2$C*H*$_3$).

^{13}C{^{1}H} NMR (benzene-d_6, 100 MHz) δ (ppm) = 154.4 (d, $^2J_{PC}$ = 18.4 Hz, 2-C), 135.6 (s, Ar-C), 135.4 (s, Ar-C), 129.7 (s, 3-C), 102.5 (s, 5-C), 46.5 (s, CH$_2$), 28.8 (d, J = 14.2 Hz), 25.5 (d, J = 31.2 Hz), 18.9 (d, J = 20.6 Hz), 17.7 (d, J = 9.1 Hz), 14.2 (s, CH$_3$).

^{31}P NMR (benzene-d_6, 160 MHz) δ (ppm) = 63.9 (s).

CHN-Analysis: found (calculated) C 65.87 (66.64) H 9.18 (9.99) N 11.87 (11.10).

HRMS (ESI): m/z calculated for [$C_{14}H_{26}N_2P$]$^+$ (M+H)$^+$ 253.18281, found 253.18241.

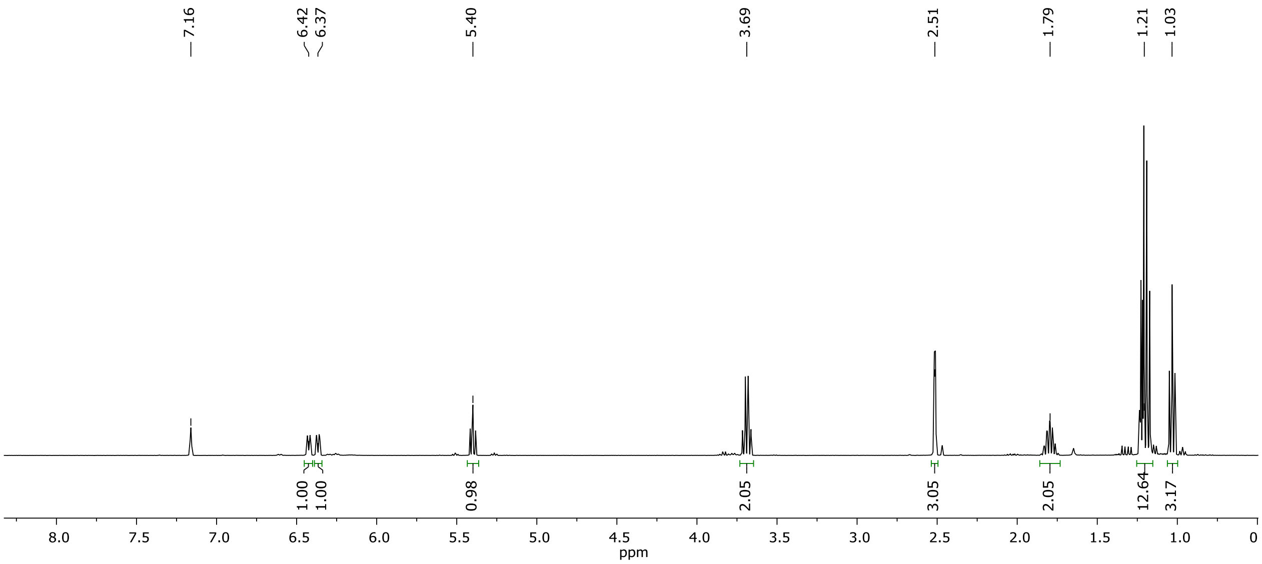

Figure S31: ^{1}H NMR spectrum (benzene-d_6, 300 K, 400 MHz) of **4d**.

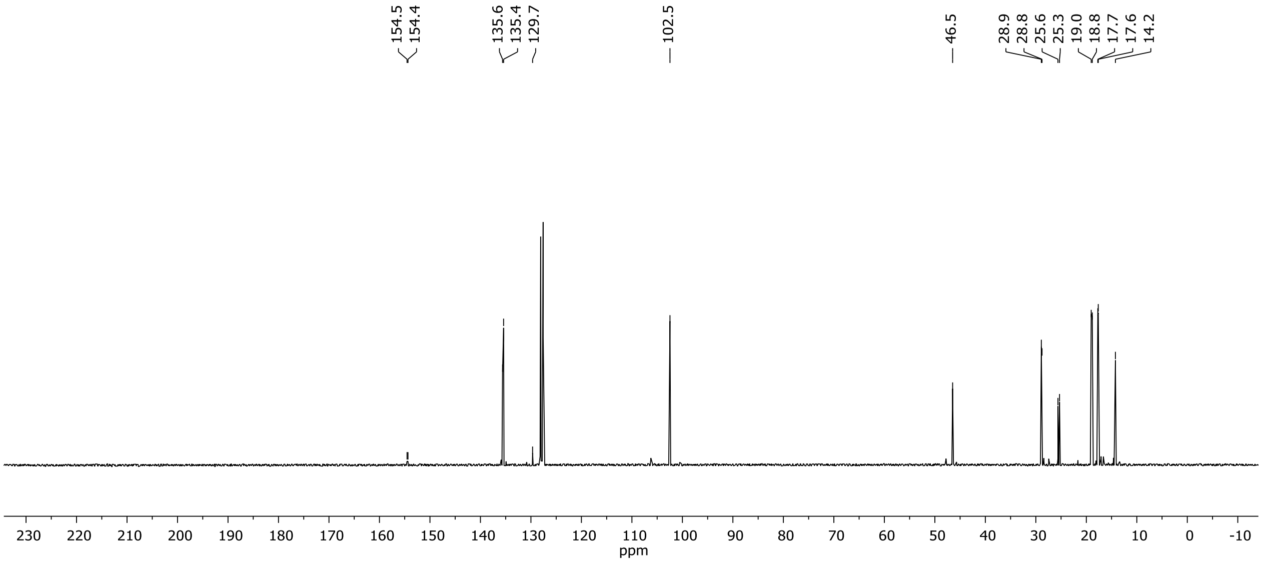

Figure S32: ^{13}C{^{1}H} NMR spectrum (benzene-d_6, 300 K, 100 MHz) of **4d**.

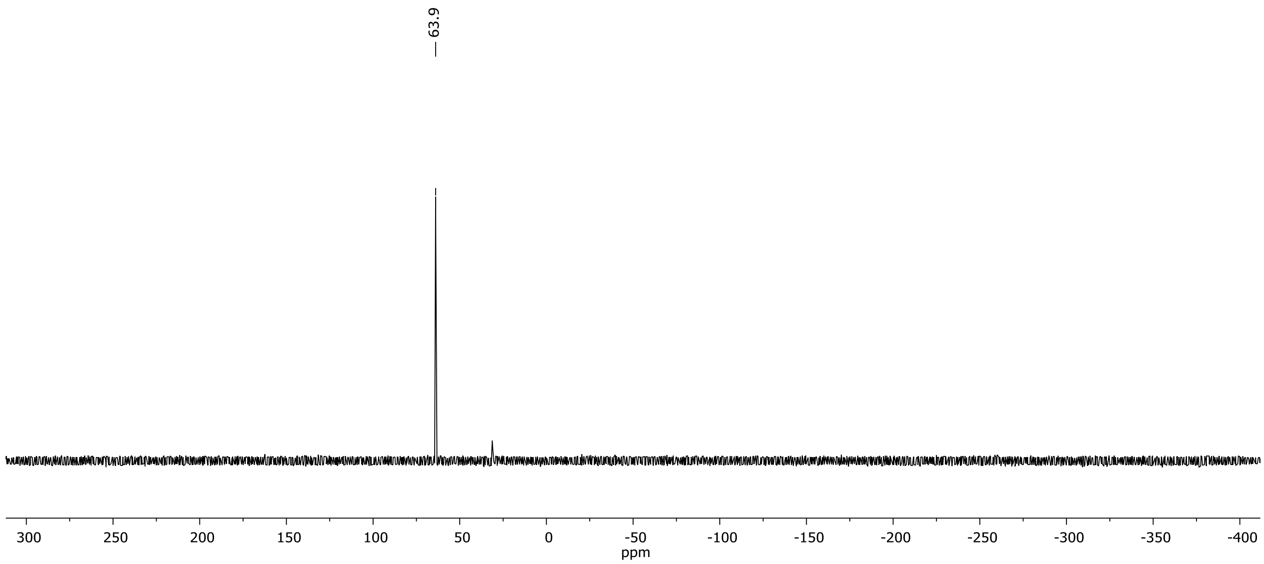

Figure S33: ^{31}P NMR spectrum (benzene-d_6, 300 K, 160 MHz) of **4d**.

Synthesis of **5a-b** and **6a-b**

Compound 5a

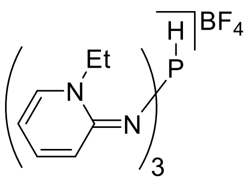

Pyridinium salt **3a** (720 mg, 3.54 mmol, 3.1 eq.) and NEt_3 (1.1 mL, 8.00 mmol, 7 eq.) were suspended in DCM (25 mL). At -78 °C PCl_3 (0.1 mL, 1.14 mmol, 1 eq.) was added dropwise and the solution was allowed to warm to room temperature. All volatile compounds were removed *in vacuo* and the residue was redissolved in a minimum amount of MeOH. $NaBF_4$ (188 mg, 1.72 mmol, 1.5 eq.) was added and the solution was cooled to -78 °C (Note that the low temperature is necessary owing to the good solubility of **5a** in MeOH). The precipitate was filtered off, washed with MeOH at -78 °C and dried at 50 °C *in vacuo* for 16 h. Yield: 423 mg (0.877 mmol, 77%).

^{1}H NMR (MeCN-d_3, 400 MHz) δ (ppm) = 7.87 (d, $^1J_{PH}$ = 544.0 Hz, PH), 7.70 (ddd, $^3J_{HH}$ = 6.8 Hz, $^4J_{HH}$ = 2.2 Hz, $^4J_{PH}$ = 2.2 Hz, 3H, 3-CH), 7.50 (ddd, $^3J_{HH}$ = 8.9 Hz, $^3J_{HH}$ = 6.8 Hz, $^4J_{HH}$ = 1.9 Hz, 3H, 4-CH), 7.24 – 7.15 (m, 3H, 6-CH), 6.54 (ddd, $^3J_{HH}$ = 6.7 Hz, $^3J_{HH}$ = 6.7 Hz, $^4J_{HH}$ = 1.4 Hz, 3H, 5-CH), 4.19 (q, $^3J_{HH}$ = 7.1 Hz, 6H, CH_2), 1.32 (t, $^3J_{HH}$ = 7.1 Hz, 9H, CH_3).

^{13}C{^{1}H} NMR (MeCN-d_3, 100 MHz) δ (ppm) = 157.7 (d, $^2J_{PC}$ = 8.5 Hz, 2-C), 140.8 (d, J = 1.8 Hz, Ar-C), 140.6 (d, J = 2.2 Hz, Ar-C), 120.3 (d, $^4J_{PC}$ = 11.8 Hz, 3-C), 111.0 (s, 5-C), 48.6 (s, CH_2), 14.6 (s, CH_3).

^{11}B{^{1}H} NMR (MeCN-d_3, 128 MHz) δ (ppm) = -1.1 (s).

^{19}F NMR (MeCN-d_3, 376 MHz) δ (ppm) = -151.8 (s, $^{10}BF_4$), -151.9 (s, $^{11}BF_4$).

^{31}P NMR (MeCN-d_3, 160 MHz) δ (ppm) = -5.9 (d, $^1J_{PH}$ = 544.0 Hz).

^{31}P{^{1}H} NMR (MeCN-d_3, 160 MHz) δ (ppm) = -5.9 (s).

HRMS (ESI): m/z calculated for $[C_{21}H_{28}N_6P]^+$ $(M-BF_4)^+$ 395.21076, found 395.21060.

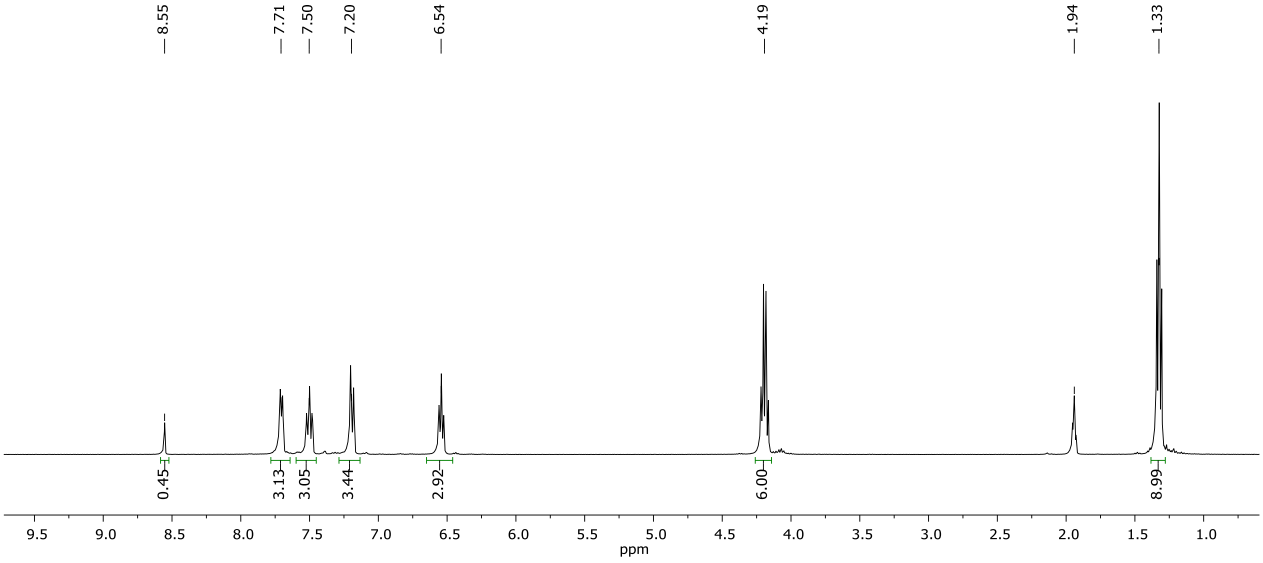

Figure S34: ^{1}H NMR spectrum (MeCN-d_3, 300 K, 400 MHz) of **5a**.

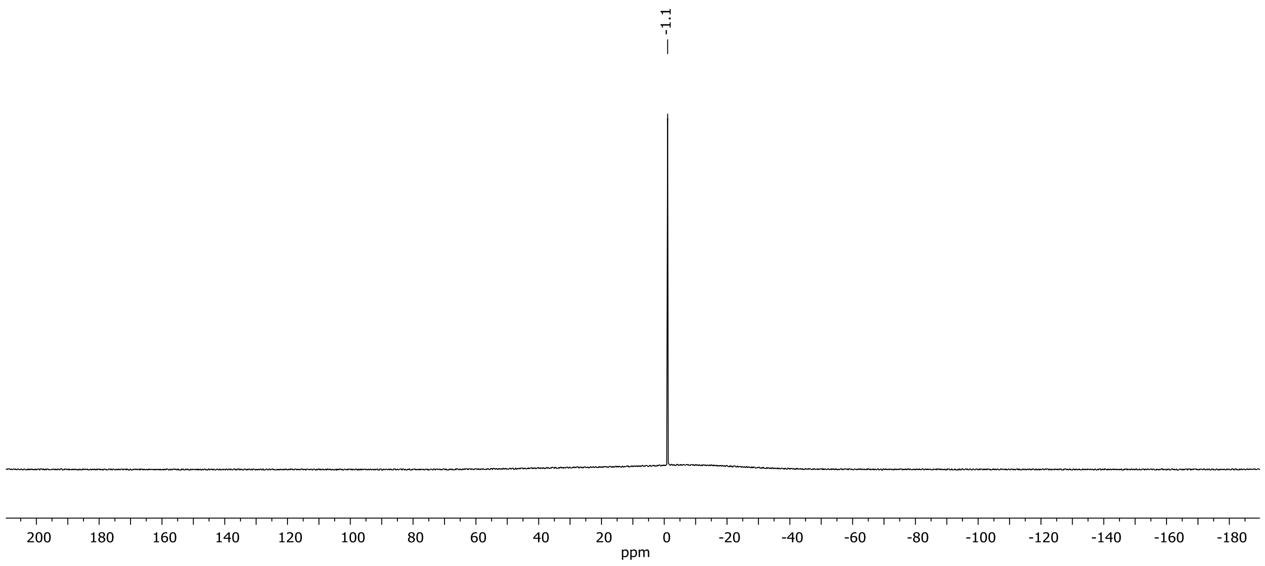

Figure S35: $^{11}B\{^1H\}$ NMR spectrum (MeCN-d_3, 300 K, 128 MHz) of **5a**.

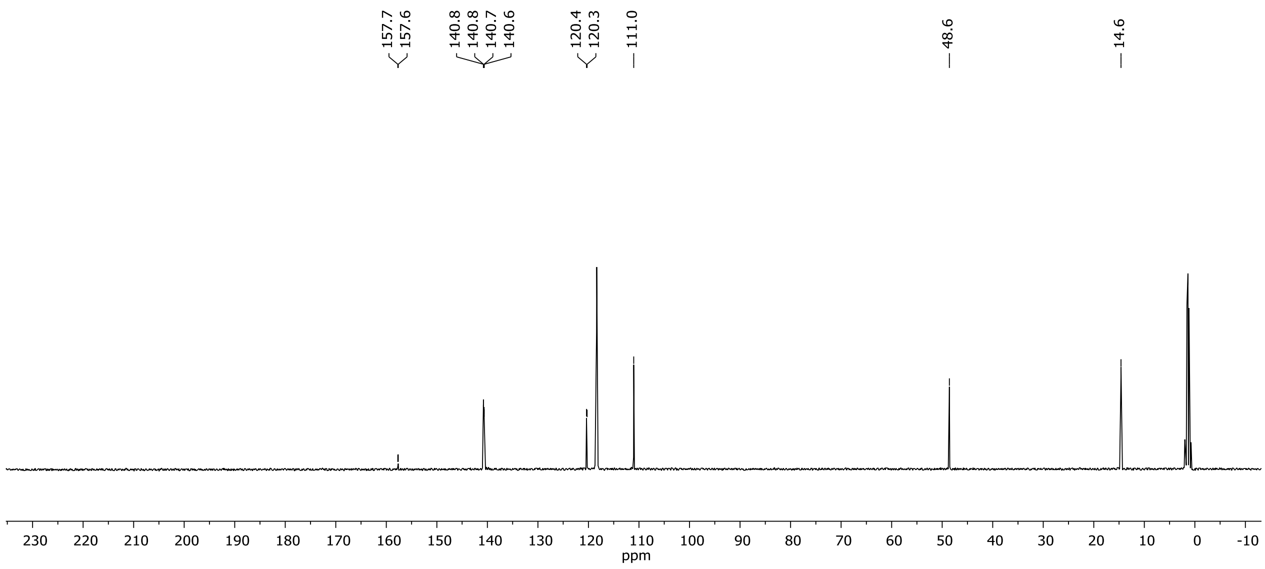

Figure S36: $^{13}C\{^1H\}$ NMR spectrum (MeCN-d_3, 300 K, 100 MHz) of **5a**.

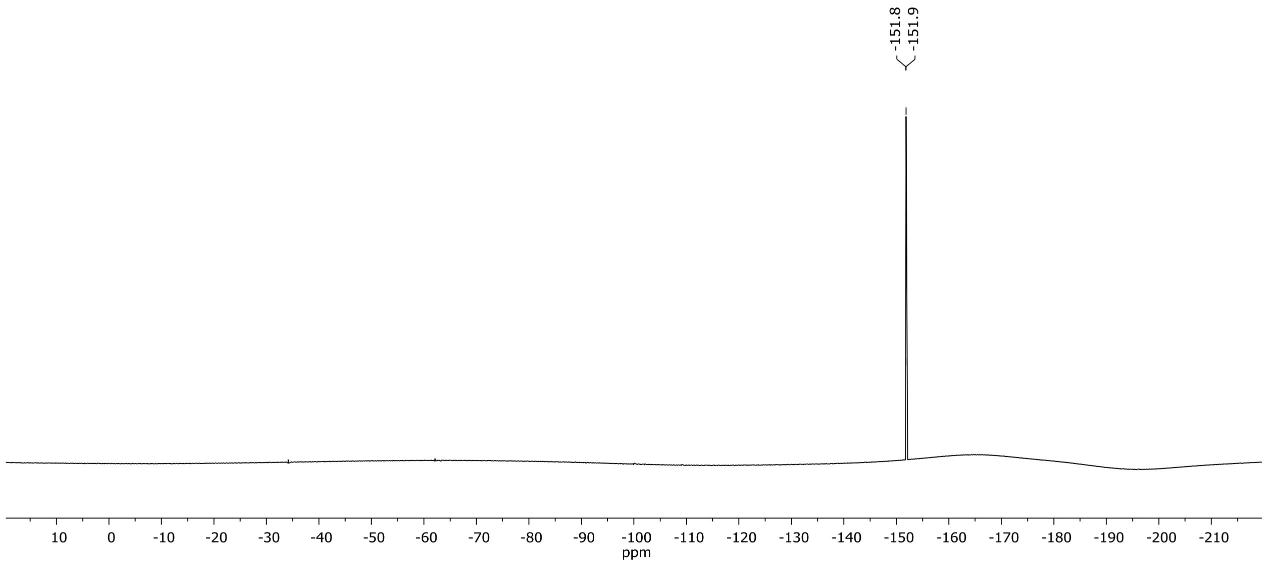

Figure S37: ^{19}F NMR spectrum (MeCN-d_3, 300 K, 376 MHz) of **5a**.

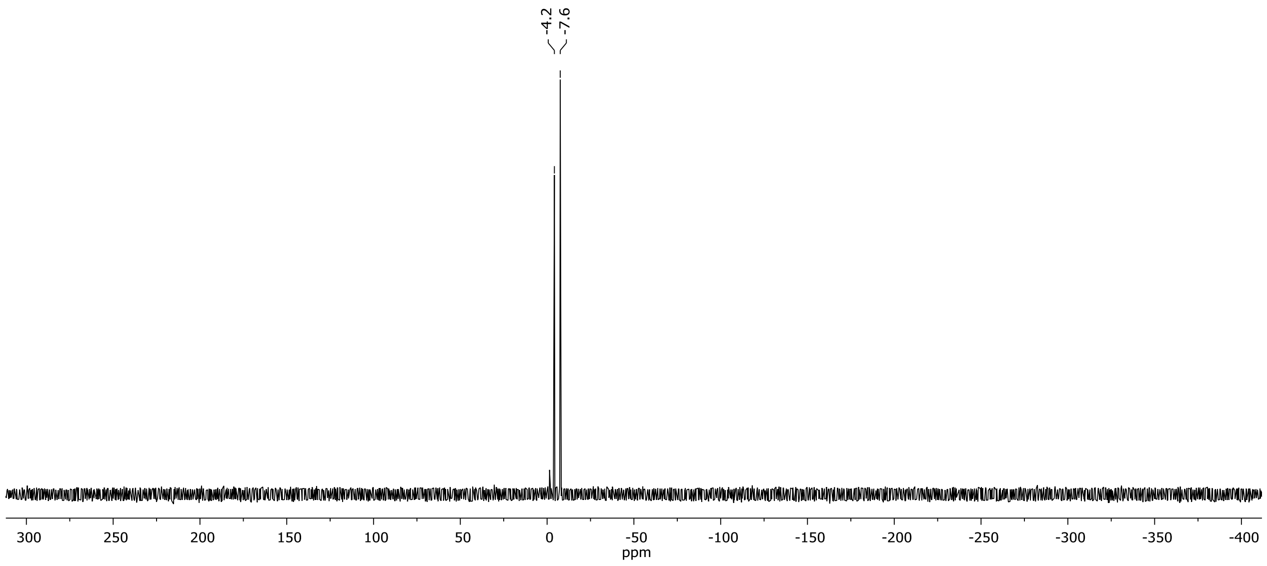

Figure S38: ^{31}P NMR spectrum (MeCN-d_3, 300 K, 160 MHz) of **5a**.

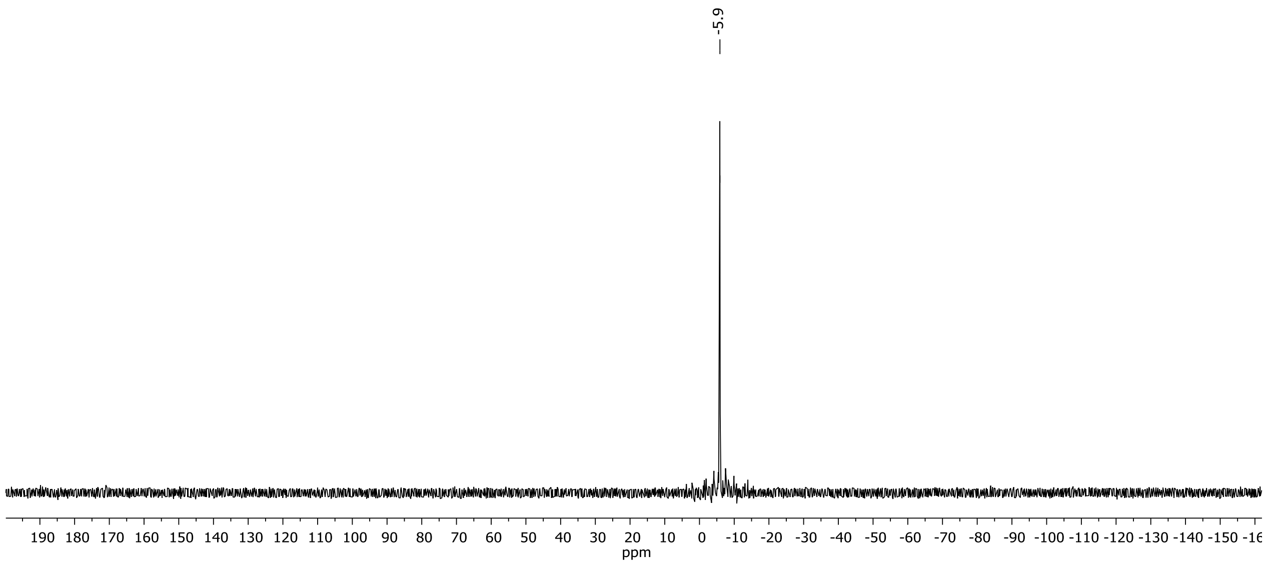

Figure S39: $^{31}P\{^{1}H\}$ NMR spectrum (MeCN-d_3, 300 K, 160 MHz) of **5a**.

Compound 5b

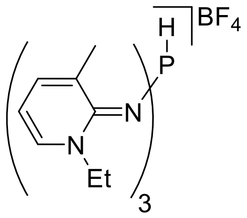

Pyridinium salt **3b** (7.45 g, 34.30 mmol, 3 eq.) and NEt_3 (12.7 mL, 91.46 mmol, 8 eq.) were dissolved in MeCN (30 mL). At -35 °C PCl_3 (1.0 mL, 11.4 mmol, 1 eq.) was added dropwise and the reaction mixture was allowed to warm at room temperature. All volatile components were removed *in vacuo* and the residue was dissolved in H_2O (50 mL). $NaBF_4$ (1.88 g, 17.1 mmol, 1.5 eq.) was added to the solution resulting in the precipitation of **5b**. The precipitate was filtered off, washed with H_2O and dried at 50 °C for 16 h *in vacuo* to afford **5b** as a pale-yellow solid. Yield: 5.10 g (9.72 mmol, 85%).

Upscaling: For 25 g Scale the following was used: **3b** (38.0 g, 175 mmol, 3 eq.), NEt_3 (50.0 mL, 362 mmol, 8 eq.), PCl_3 (5.1 mL, 58.3 mmol, 1 eq.) and $NaBF_4$ (7.68 g, 70.0 mmol, 1.2 eq.). Yield: 23.3 g (44.5 mmol, 76%).

^{1}H NMR (MeCN-d_3, 400 MHz) δ (ppm) = 8.97 (d, $^{1}J_{PH}$ = 571.3 Hz, 1H, PH), 7.61 (ddd, $^{3}J_{HH}$ = 6.8 Hz, $^{4}J_{HH}$ = 2.2 Hz, $^{4}J_{HH}$ = 2.2 Hz, 3H, CH), 7.46 (ddd, $^{3}J_{HH}$ = 7.1 Hz, $^{4}J_{HH}$ = 1.4 Hz, $^{4}J_{HH}$ = 1.4 Hz, 3H, CH), 6.49 (dd, $^{3}J_{HH}$ = 6.8 Hz, $^{3}J_{HH}$ = 6.8 Hz, 3H, 5-CH), 4.12 (q, $^{3}J_{HH}$ = 7.1 Hz, 6H, CH_2), 2.41 (s, 9H, Ar-CH_3), 1.19 (t, $^{3}J_{HH}$ = 7.1 Hz, 9H, CH_2CH_3).

$^{1}H\{^{31}P\}$ NMR (MeCN-d_3, 400 MHz) δ (ppm) = 8.97 (s, PH), 7.62 (d, $^{3}J_{HH}$ = 6.6 Hz, CH), 7.45 (d, $^{3}J_{HH}$ = 7.1 Hz, CH), 6.49 (dd, $^{3}J_{HH}$ = 6.8 Hz, $^{3}J_{HH}$ = 6.8 Hz, 5-CH), 4.12 (q, $^{3}J_{HH}$ = 7.1 Hz, CH_2), 2.41 (s, Ar-CH_3), 1.19 (t, $^{3}J_{HH}$ = 7.1 Hz, CH_2CH_3).

$^{11}B\{^{1}H\}$ NMR (MeCN-d_3, 128 MHz) δ (ppm) = -1.2 (s).

$^{13}C\{^{1}H\}$ NMR (MeCN-d_3, 100 MHz) δ (ppm) = 154.7 (d, $^{2}J_{PC}$ = 11.4 Hz, 2-C), 140.1 (s, Ar-C), 138.2 (d, J_{PC} = 1.1 Hz, Ar-C) , 131.8 (d, $^{3}J_{PC}$ = 3.4 Hz, 3-C), 111.0 (s, 5-C), 49.5 (s, CH_2), 21.4 (d, $^{4}J_{PC}$ = 1.6 Hz, Ar-CH_3), 14.8 (s, CH_2CH_3).

^{19}F NMR (MeCN-d_3, 376 MHz) δ (ppm) = -151.8 (s, $^{10}BF_4$), -151.8 (s, $^{11}BF_4$).

^{31}P NMR (MeCN-d_3, 160 MHz) δ (ppm) = -29.6 (d, $^1J_{PH}$ = 571.6 Hz).

^{31}P{^{1}H} NMR (MeCN-d_3, 160 MHz) δ (ppm) = -29.6 (s).

CHN-Analysis: found (calculated) C 54.35 (54.97) H 6.45 (6.54) N 15.97 (16.03).

HRMS (ESI): m/z calculated for $[C_{24}H_{34}N_6P]^+$ $(M-BF_4)^+$ 437.25881, found 437.25753.

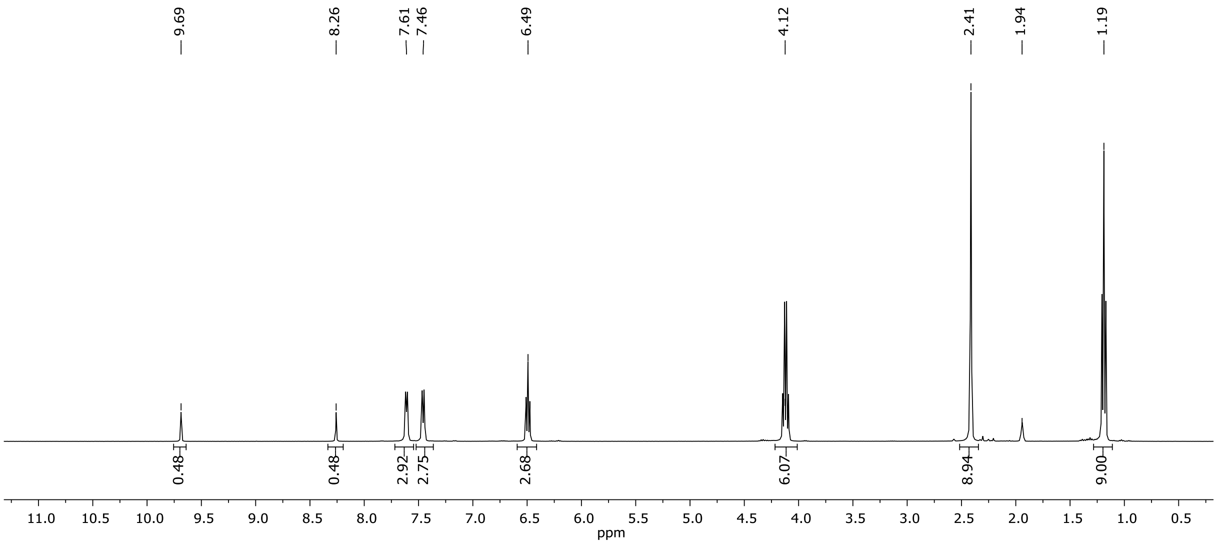

Figure S40: ^{1}H NMR spectrum (MeCN-d_3, 300 K, 400 MHz) of **5b**.

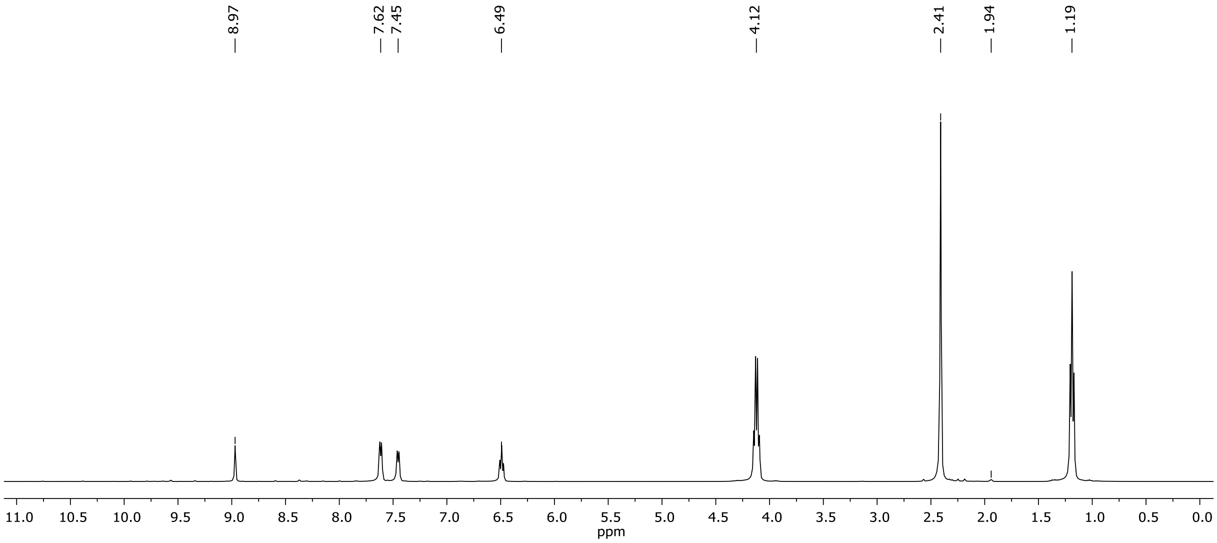

Figure S41: ^{1}H{^{31}P} NMR spectrum (MeCN-d_3, 300 K, 400 MHz) of **5b**.

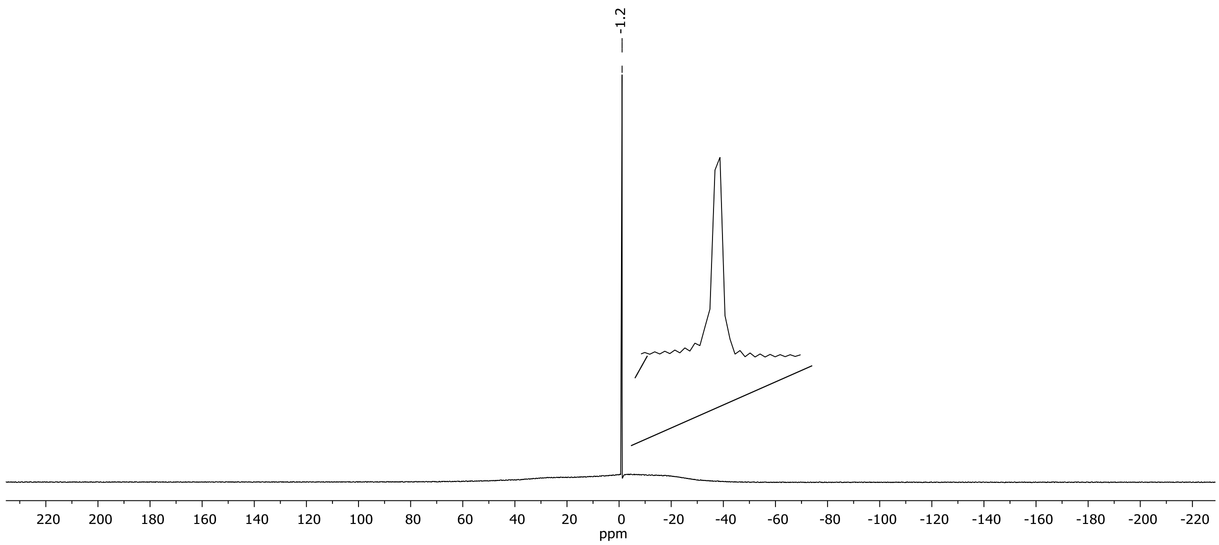

Figure S42: $^{11}B\{^{1}H\}$ NMR spectrum (MeCN-d_3, 300 K, 128 MHz) of **5b**.

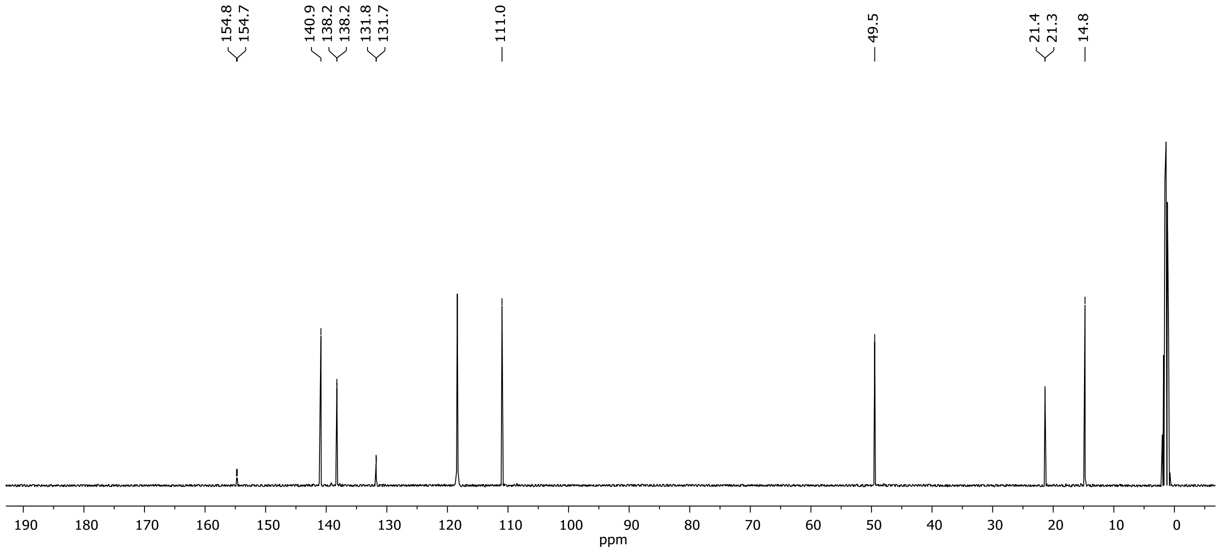

Figure S43: $^{13}C\{^{1}H\}$ NMR spectrum (MeCN-d_3, 300 K, 100 MHz) of **5b**.

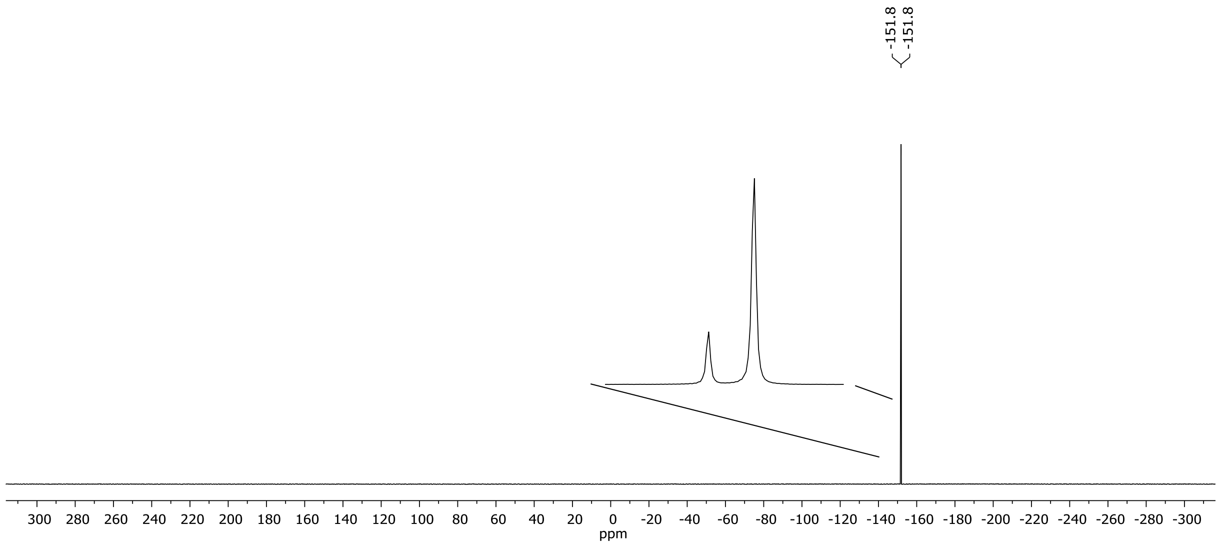

Figure S44: ^{19}F NMR spectrum (MeCN-d_3, 300 K, 376 MHz) of **5b**.

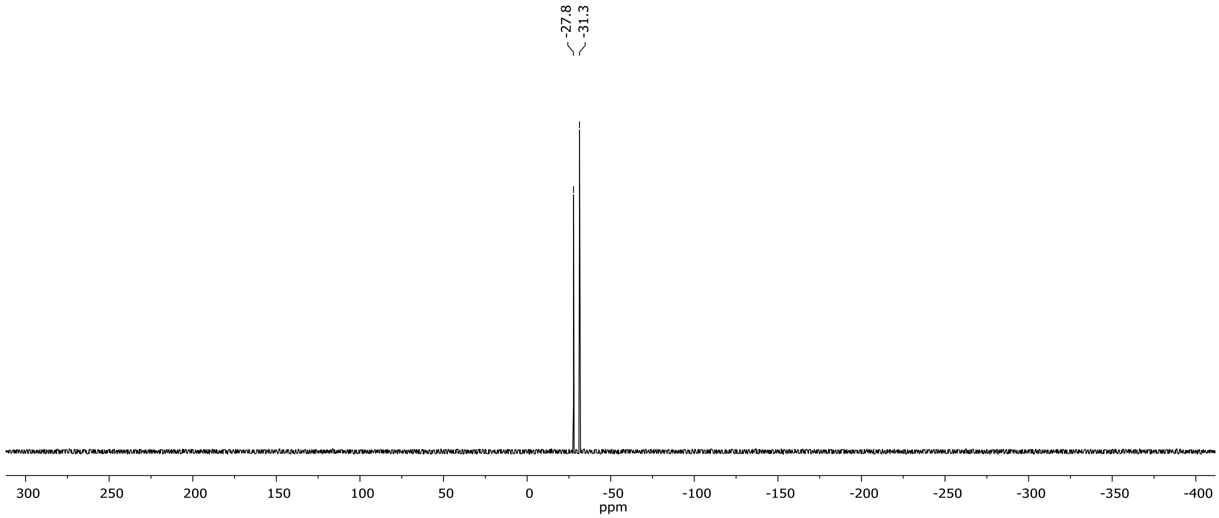

Figure S45: ^{31}P NMR spectrum (MeCN-d_3, 300 K, 160 MHz) of **5b**.

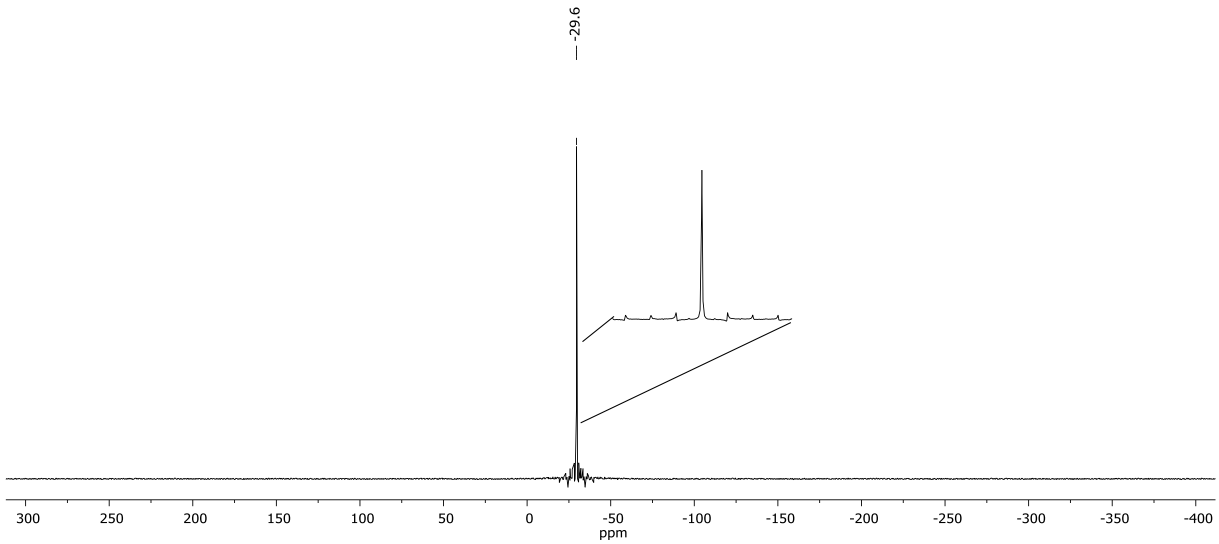

Figure S46: $^{31}P\{^1H\}$ NMR spectrum (MeCN-d_3, 300 K, 160 MHz) of **5b**.

Compound 6a

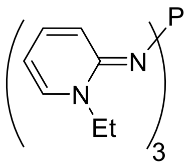

Phosphonium salt **5a** (100 mg, 0.207 mmol, 1 eq.) and KHMDS (41 mg, 0.207 mmol, 1 eq.) were suspended in toluene (5 mL) and stirred for 16 h. All volatile compounds were removed *in vacuo* and the residue was extracted with toluene (20 mL) to afford **6a** as a red wax-like solid. Yield: 66 mg (0.168 mmol, 81%).

^{1}H NMR (benzene-d_6, 400 MHz) δ (ppm) = 7.62 (ddd, $^3J_{HH}$ = 9.4 Hz, $^4J_{HH}$ = 1.9 Hz, $^4J_{PH}$ = 1.9 Hz, 3H, 3-CH), 6.59 (ddd, $^3J_{HH}$ = 9.4 Hz, $^3J_{HH}$ = 6.2 Hz, $^4J_{HH}$ = 2.0 Hz, 3H, 4-CH), 6.59 (m, 3H, 6-CH), 5.36 (dd, $^3J_{HH}$ = 6.5 Hz, $^3J_{HH}$ = 6.5 Hz, $^4J_{HH}$ = 1.4 Hz, 3H, 5-CH), 3.65 (q, $^3J_{HH}$ = 7.1 Hz, 6H, CH_2), 1.02 (t, $^3J_{HH}$ = 7.1 Hz, 9H, CH_3).

^{13}C{^{1}H} NMR (benzene-d_6, 100 MHz) δ (ppm) = 153.1 (d, $^2J_{PC}$ = 17.0 Hz, 2-C), 137.7 (s, 6-C), 133.2 (d, $^5J_{PC}$ = 3.4 Hz, 4-C), 120.8 (d, $^4J_{PC}$ = 25.6 Hz, 3-C), 102.1 (s, 5-C), 45.4 (s, CH_2), 14.3 (s, CH_3).

^{31}P NMR (benzene-d_6, 160 MHz) δ (ppm) = 86.4 (s).

HRMS (ESI): m/z calculated for $[C_{21}H_{28}N_6P]^+$ $(M+H)^+$ 395.21076, found 395.21083.

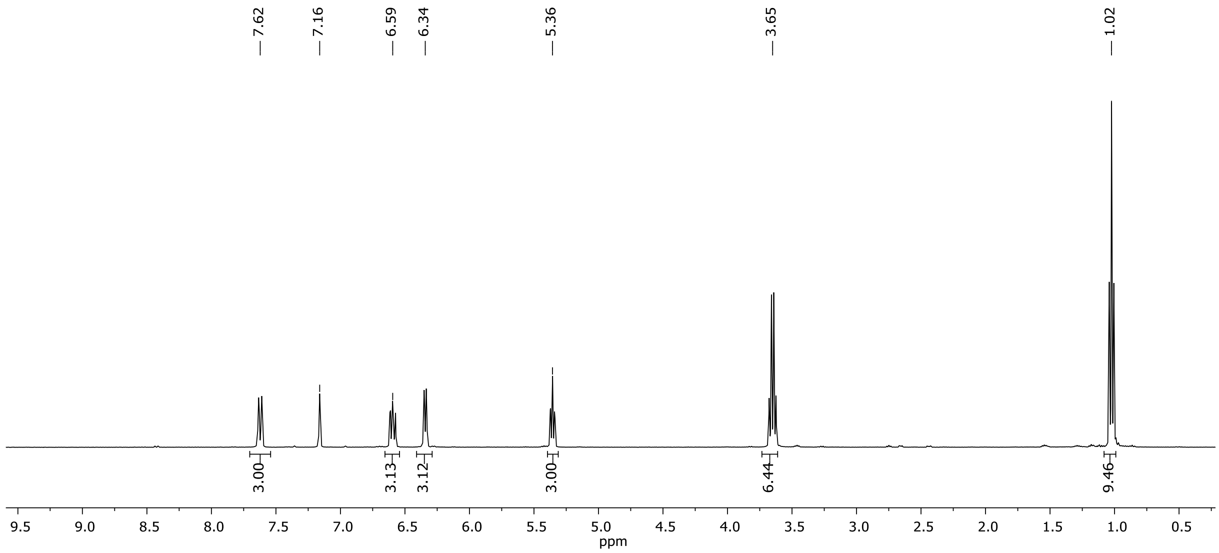

Figure S47: ^{1}H NMR spectrum (benzene-d_6, 300 K, 400 MHz) of **6a**.

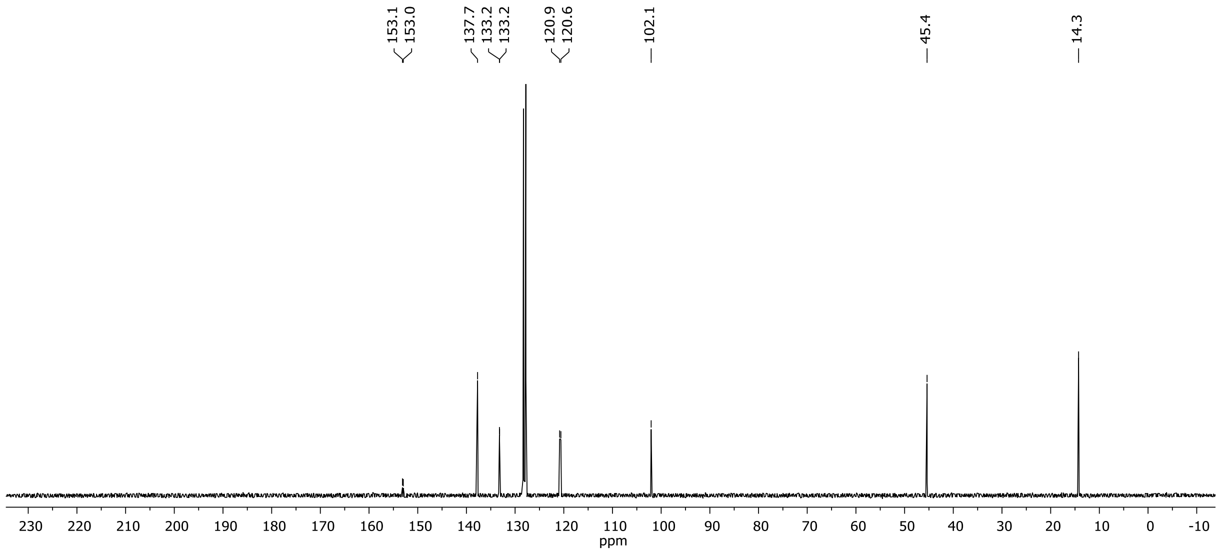

Figure S48: $^{13}C\{^{1}H\}$ NMR spectrum (benzene-d_6, 300 K, 100 MHz) of **6a**.

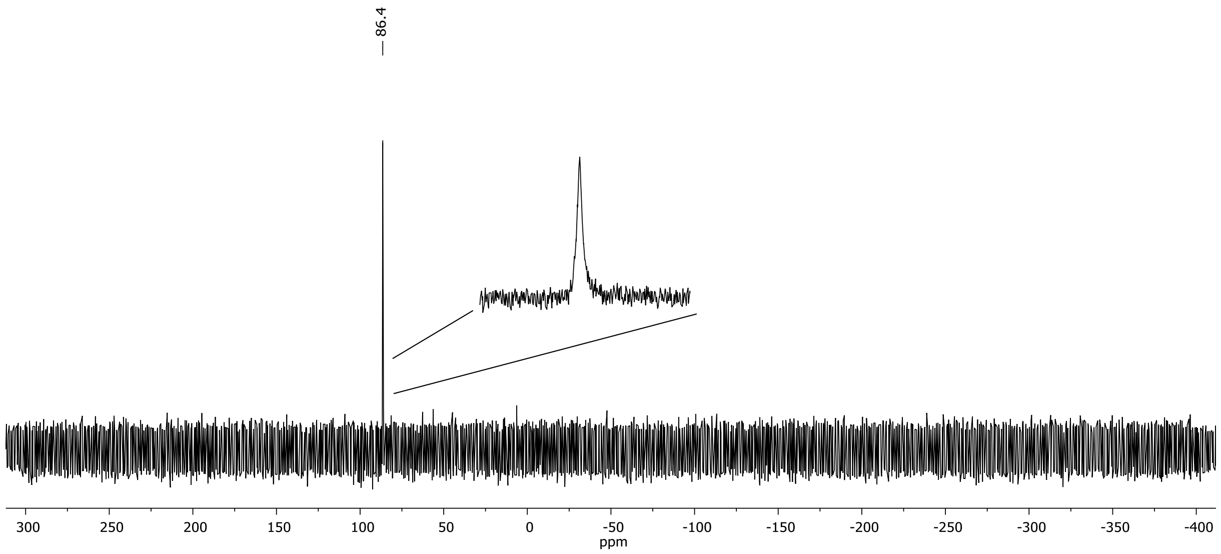

Figure S49: ^{31}P NMR spectrum (benzene-d_6, 300 K, 160 MHz) of **6a**.

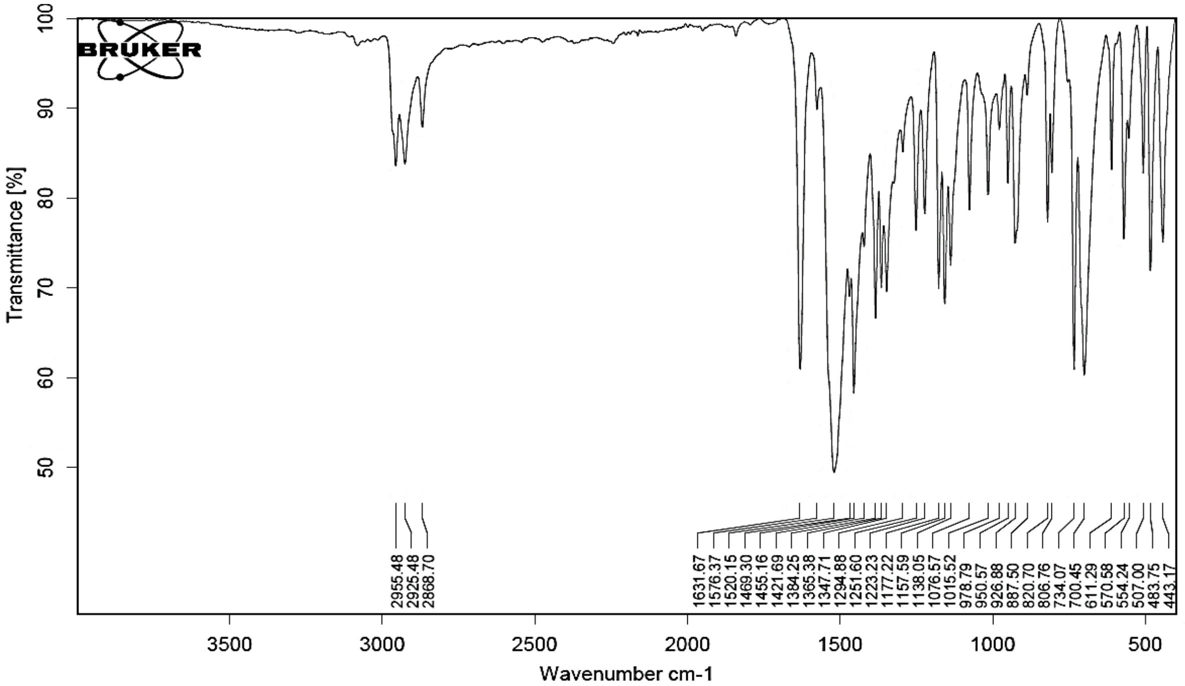

Figure S50: FT-IR spectrum of **6a** (solid).

Compound 6b

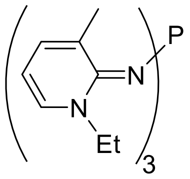

Phosphonium salt **5b** (858 mg, 1.64 mmol, 1 eq.) and KHMDS (326 mg, 1.64 mmol, 1 eq.) were suspended in toluene (10 mL) and stirred for 16 h. All volatile compounds were removed *in vacuo* and the residue was extracted with toluene (2 x 20 mL) to afford **6b** as a dark red solid. Yield: 687 mg (1.57 mmol, 96%).

^{1}H NMR (benzene-d_6, 400 MHz) δ (ppm) = 6.53 (ddd, $^{3}J_{HH}$ = 6.3 Hz, $^{4}J_{HH}$ = 1.8 Hz, $^{4}J_{HH}$ = 1.8 Hz, 3H, CH), 6.43 (dd, $^{3}J_{HH}$ = 6.7 Hz, $^{4}J_{HH}$ = 2.0 Hz, 3H, CH), 5.37 (dd, $^{3}J_{HH}$ = 6.6 Hz, $^{3}J_{HH}$ = 6.6 Hz, 3H, 5-CH), 3.79 (q, $^{3}J_{HH}$ = 7.1 Hz, 6H, CH_2), 3.71 (m, 9H, Ar-CH_3), 1.12 (t, $^{3}J_{HH}$ = 7.0 Hz, 9H, CH_2CH_3).

^{13}C{^{1}H} NMR (benzene-d_6, 100 MHz) δ (ppm) = 148.7 (d, $^{2}J_{PC}$ = 14.0 Hz, 2-C), 135.6 (s, Ar-C), 133.7 (s, Ar-C) , 130.9 (s, 3-C), 101.5 (s, 5-C), 46.6 (s, CH_2), 24.1 (d, $^{4}J_{PC}$ = 28.1 Hz, Ar-CH_3), 14.7 (d, $^{5}J_{PC}$ = 1.8 Hz, CH_2CH_3).

^{31}P NMR (benzene-d_6, 160 MHz) δ (ppm) = 83.5 (s).

CHN-Analysis: found (calculated) C 65.04 (66.03) H 7.23 (7.62) N 18.89 (19.25).

HRMS (ESI): m/z calculated for $[C_{24}H_{34}N_6P]^+$ $(M+H)^+$ 437.25771, found 437.25772.

IR (neat): $\tilde{\nu}$ = 2962 (w), 2931 (w), 1625 (s), 1598 (w), 1563 (s), 1522 (vs), 1456 (s), 1439 (s), 1426 (s), 1376 (m), 1349 (m), 1336 (m), 1253 (w), 1191 (s), 1162 (m), 1104 (s), 1081 (m).

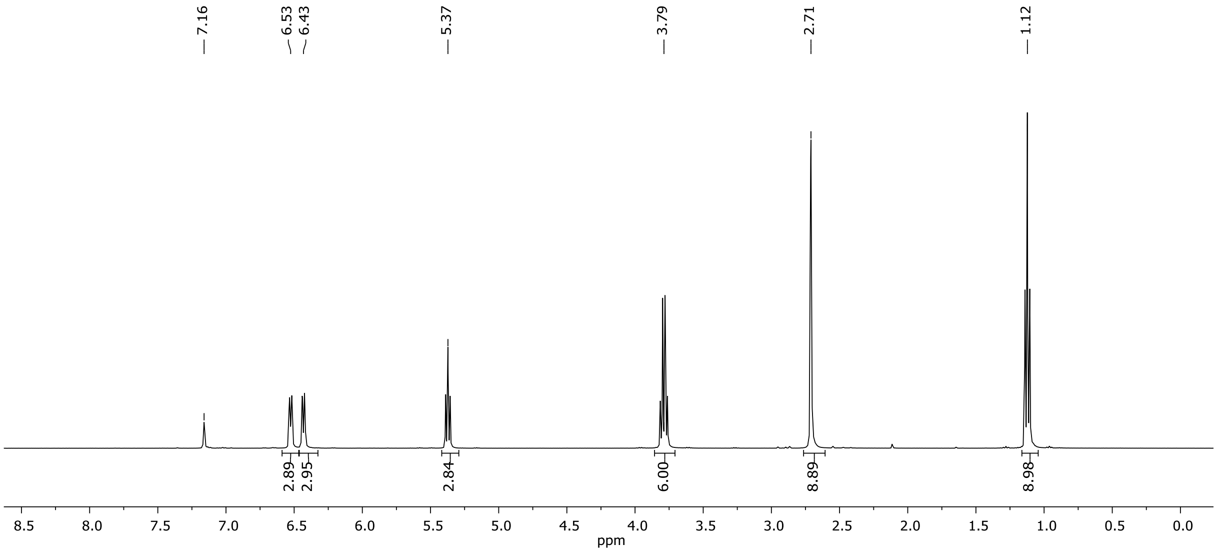

Figure S51: ^{1}H NMR spectrum (benzene-d_6, 300 K, 400 MHz) of **6b**.

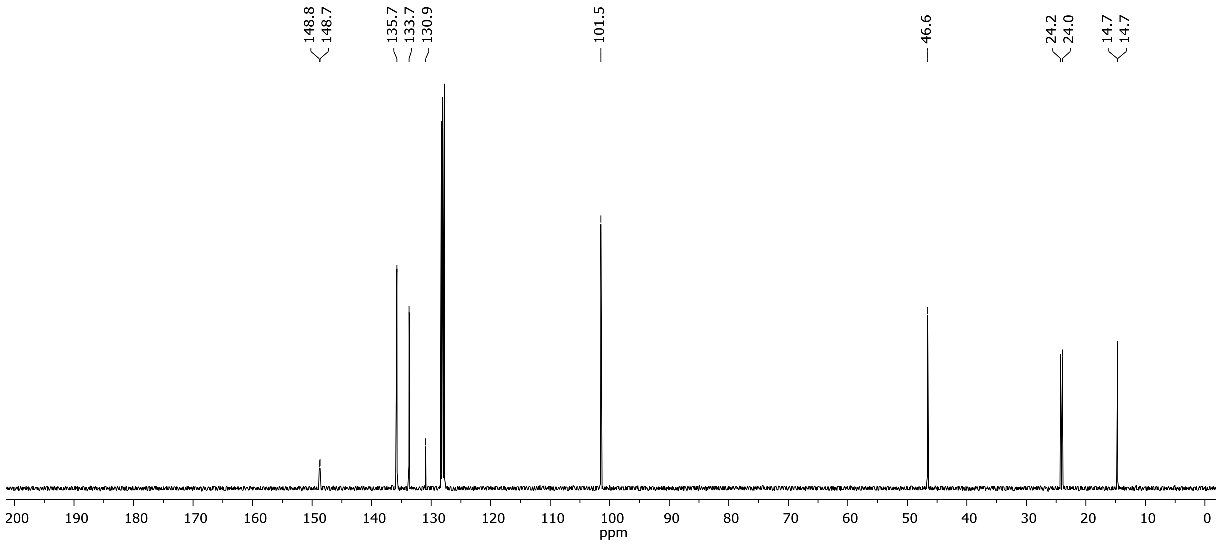

Figure S52: $^{13}C\{^1H\}$ NMR spectrum (benzene-d_6, 300 K, 100 MHz) of **6b**.

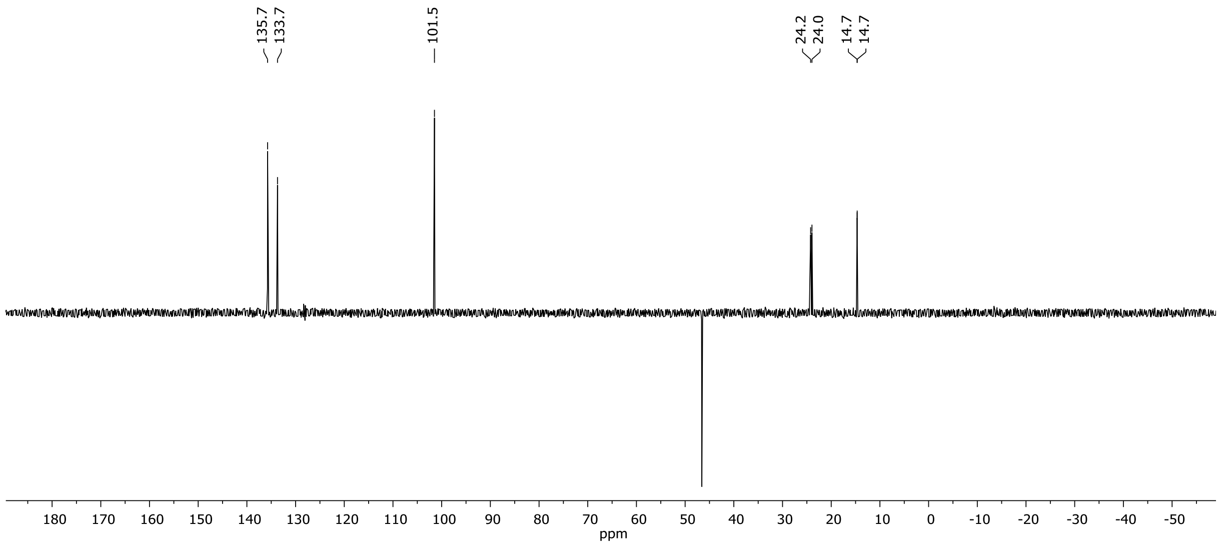

Figure S53: $^{13}C\{^1H\}$Dept135 NMR spectrum (benzene-d_6, 300 K, 100 MHz) of **6b**.

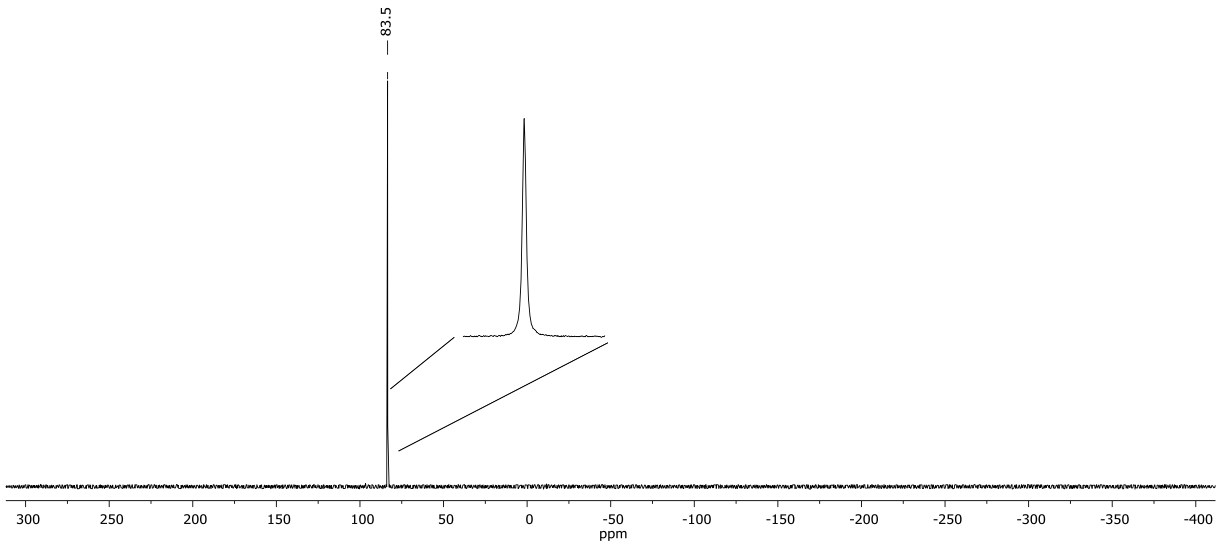

Figure S54: ^{31}P NMR spectrum (benzene-d_6, 300 K, 160 MHz) of **6b**.

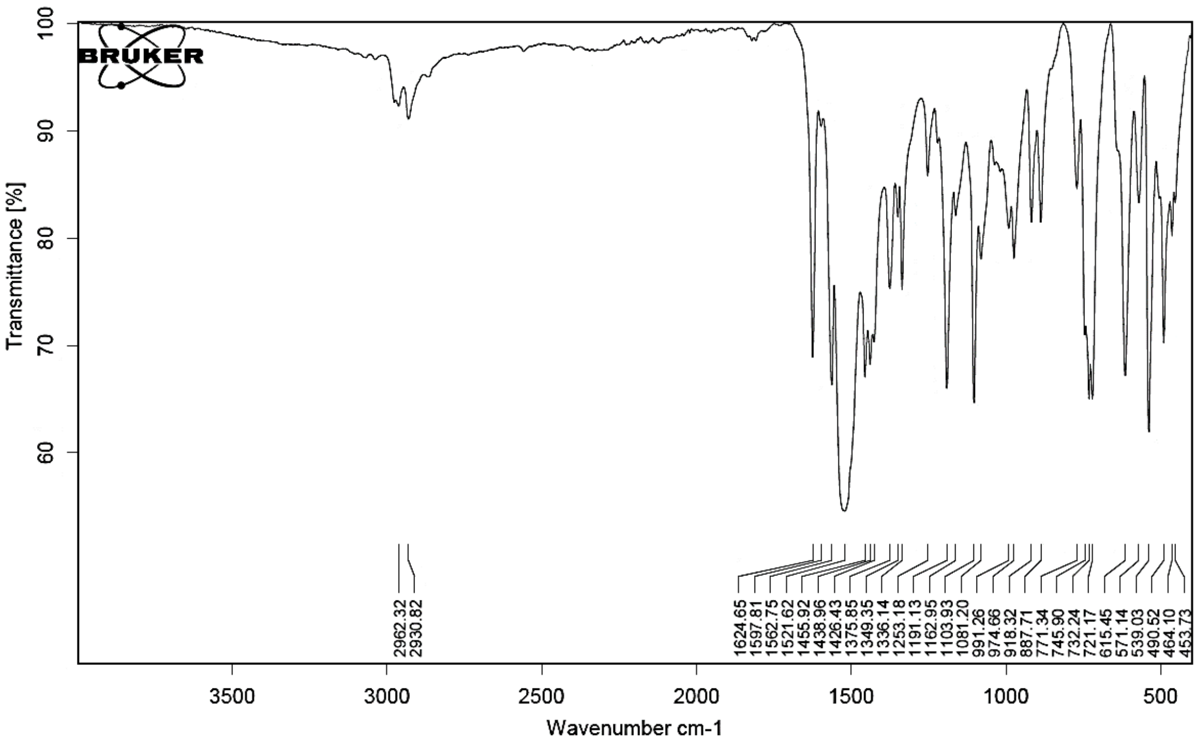

Figure S55: FT-IR spectrum of **6b** (solid).

Synthesis of **7** and **8**

Compound 7

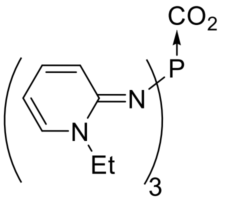

Phosphine **6a** (30 mg, 0.063 mmol) was dissolved in THF (5 mL). After removal of the atmosphere the solution was pressurized with CO_2 (2 bar). The precipitate was filtered off and dried *in vacuo* yielding **7** as yellow solid in quantitative yield.

^{1}H NMR (MeCN-d_3, 400 MHz) δ (ppm) = 7.55 (ddd, $^{3}J_{HH}$ = 6.8 Hz, $^{4}J_{HH}$ = 2.1 Hz, $^{4}J_{PH}$ = 2.1 Hz, 3H, 3-CH), 7.49 (d, $^{3}J_{HH}$ = 9.4 Hz, 3H, 4-CH), 7.32 (ddd, $^{3}J_{HH}$ = 8.9 Hz, $^{3}J_{HH}$ = 6.7 Hz, $^{4}J_{HH}$ = 1.9 Hz, 3H, 6-CH), 6.33 (ddd, $^{3}J_{HH}$ = 6.7 Hz, $^{3}J_{HH}$ = 6.7 Hz, $^{4}J_{HH}$ = 1.5 Hz, 3H, 5-CH), 4.12 (q, $^{3}J_{HH}$ = 7.1 Hz, 6H, CH_2), 1.31 (t, $^{3}J_{HH}$ = 7.1 Hz, 9H, CH_3).

^{13}C{^{1}H} NMR (MeCN-d_3, 100 MHz) δ (ppm) = 156.7 (d, $^{2}J_{PC}$ = 5.8 Hz, 2-C), 138.6 (d, J_{PC} = 1.8 Hz, Ar-C), 137.4 (s, Ar-C), 121.7 (d, J_{PC} = 7.8 Hz, Ar-C), 108.2 (s, 5-C), 46.9 (s, CH_2), 13.9 (s, CH_3).

^{31}P NMR (MeCN-d_3, 160 MHz) δ (ppm) = -3.7 (s).

HRMS (ESI): m/z calculated for $[C_{21}H_{28}N_6P]^+$ $(M-CO_2+H)^+$ 395.21076, found 395.21250.

IR (neat): $\tilde{\nu}$ = 2976 (w), 2932 (w), 1632 (s), 1566 (w), 1541 (m), 1505 (vs), 1456 (s), 1389 (s), 1349 (m), 1290 (m), 1273 (m), 1164 (m), 1144 (w), 1032 (m).

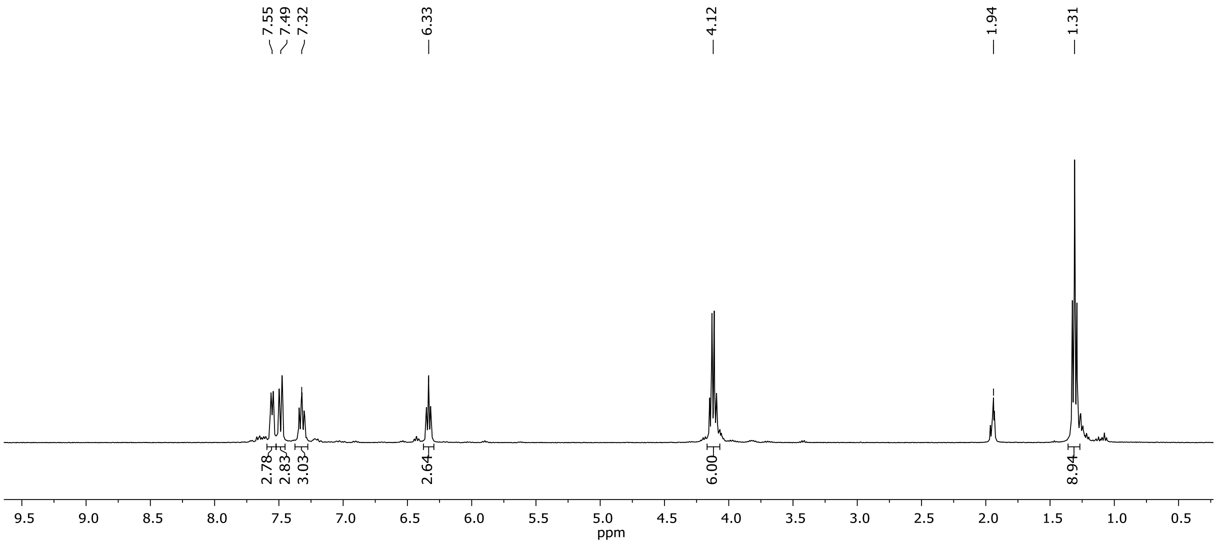

Figure S56: ^{1}H NMR spectrum (MeCN-d_3, 300 K, 400 MHz) of **7**.

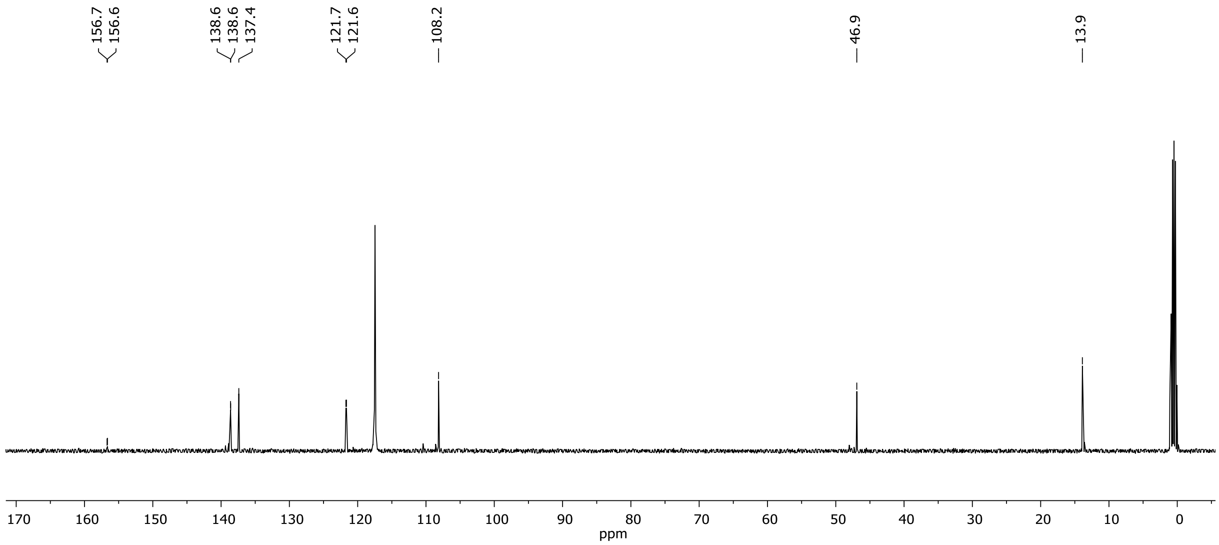

Figure S57: $^{13}C\{^{1}H\}$ NMR spectrum (MeCN-d_3, 300 K, 100 MHz) of **7**.

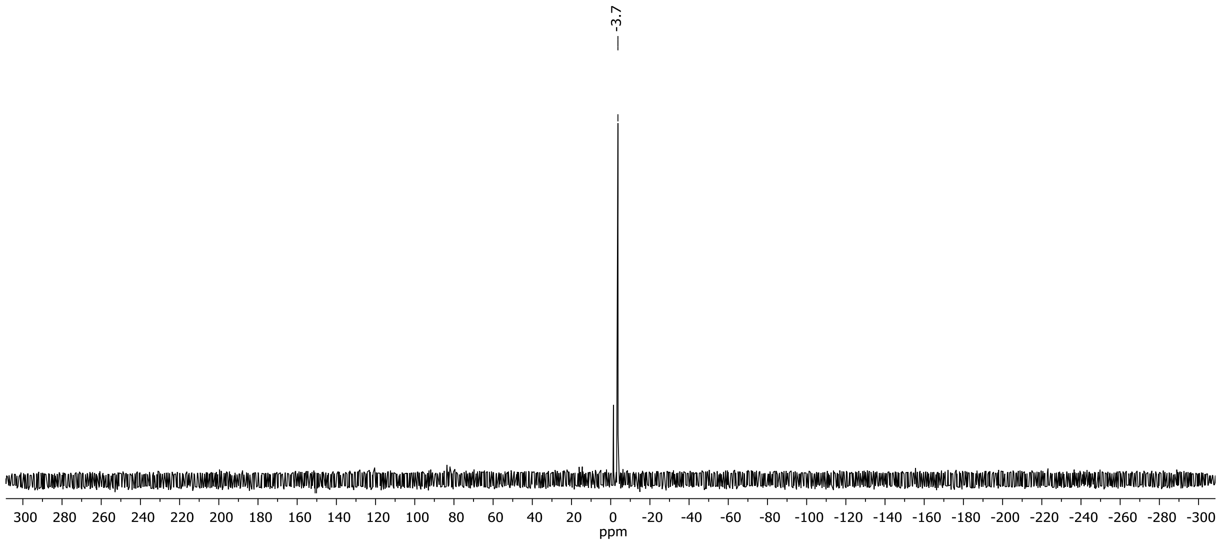

Figure S58: ^{31}P NMR spectrum (MeCN-d_3, 300 K, 160 MHz) of **7**.

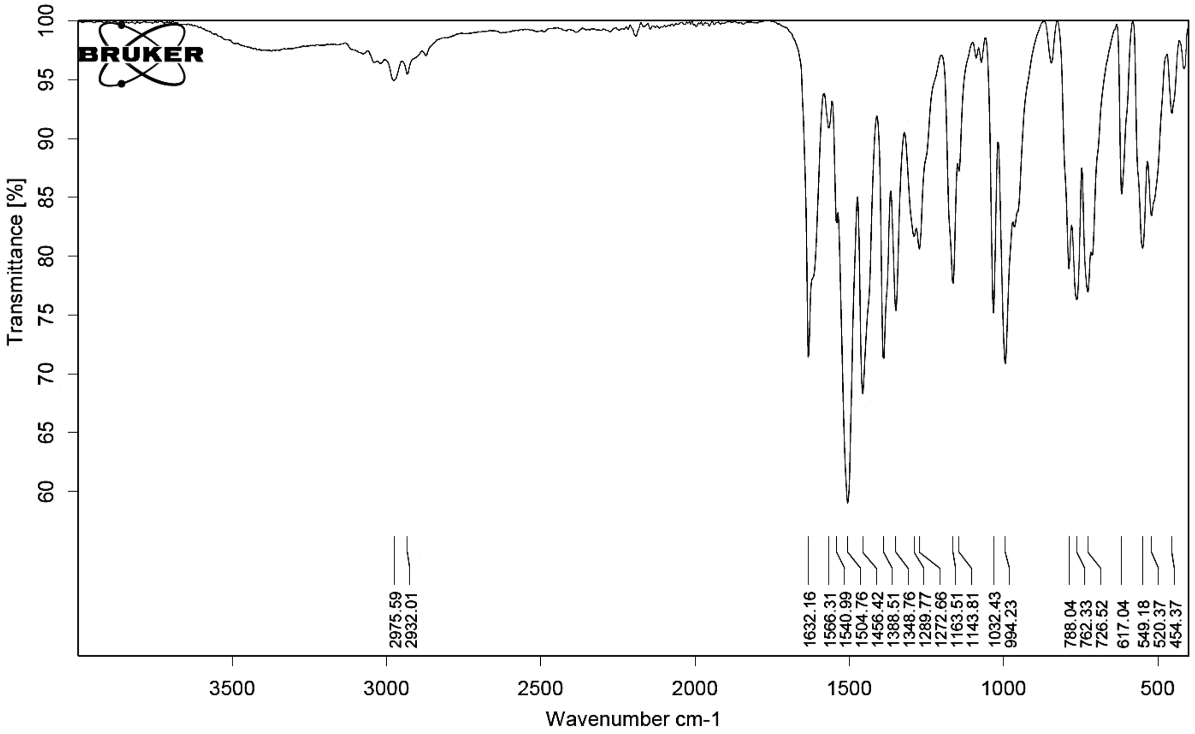

Figure S59: FT-IR spectrum of **7** (solid).

Compound 8

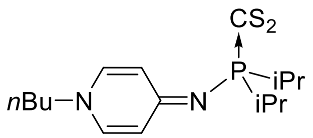

Phosphine **2d** (30 mg, 0.113 mmol, 1 eq.) was dissolved in THF (3 mL). CS_2 (0.1 mL, 13 mg, 0.171 mmol, 1.5 eq) was added dropwise and the reaction mixture was stirred for 12 h. After removing all volatile compounds *in vacuo*, the colourless product was isolated in quantitative yield.

^{1}H NMR (benzene-d_6, 400.1 MHz) δ (ppm) = 6.73 (m, 4H, Ar-H), 3.16 (t, $^3J_{HH}$ = 7.4 Hz, 2H, N-CH_2), 3.08 (m, 2H, C*H*(CH_3)$_2$), 1.36 (m, 12H, CH(C*H*$_3$)$_2$), 1.08 (m, 2H, N-CH_2-C*H*$_2$), 0.87 (m, 2H, N-(CH_2)$_2$-C*H*$_2$), 0.62 (t, $^3J_{HH}$ = 7.3 Hz, 3H, N-(CH_2)$_3$-C*H*$_3$).

^{13}C{^{1}H} NMR (benzene-d_6, 100.6 MHz) δ (ppm) = 241.3 (d, $^1J_{PC}$ = 26.0 Hz, *C*S_2), 167.1 (aryl-C), 139.0 (aryl-C), 119.8 (aryl-C), 56.6 (s, N-CH_2), 32.6 (s, N-CH_2-*C*H_2), 27.4 (d, $^1J_{PC}$ = 64.9 Hz, *C*H(CH_3)$_2$), 19.5 (s, N-(CH_2)$_2$-*C*H_2), 17.5 (d, $^2J_{PC}$ = 1.8 Hz, CH(*C*H_3)$_2$), 17.1 (d, $^2J_{PC}$ = 3.4 Hz, CH(*C*H_3)$_2$), 13.6 (s, N-(CH_2)$_3$-*C*H_3).

^{31}P NMR (benzene-d_6, 162.0 MHz) δ (ppm) = 28.7 (m).

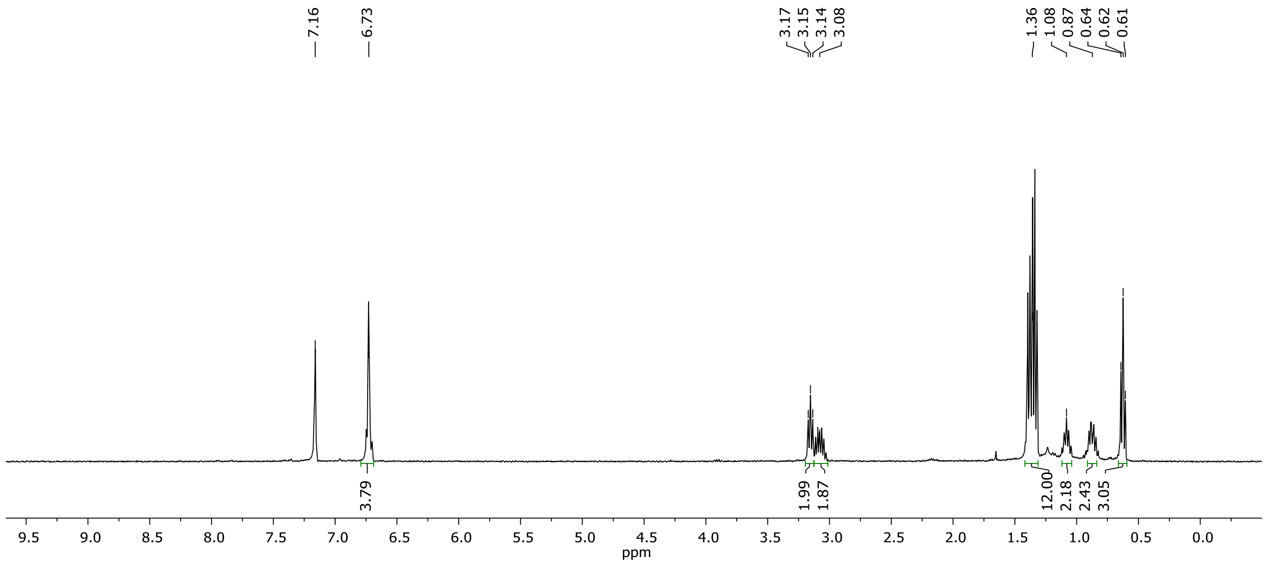

Figure S60: ^{1}H NMR spectrum (benzene-d_6, 300 K, 400 MHz) of **8**.

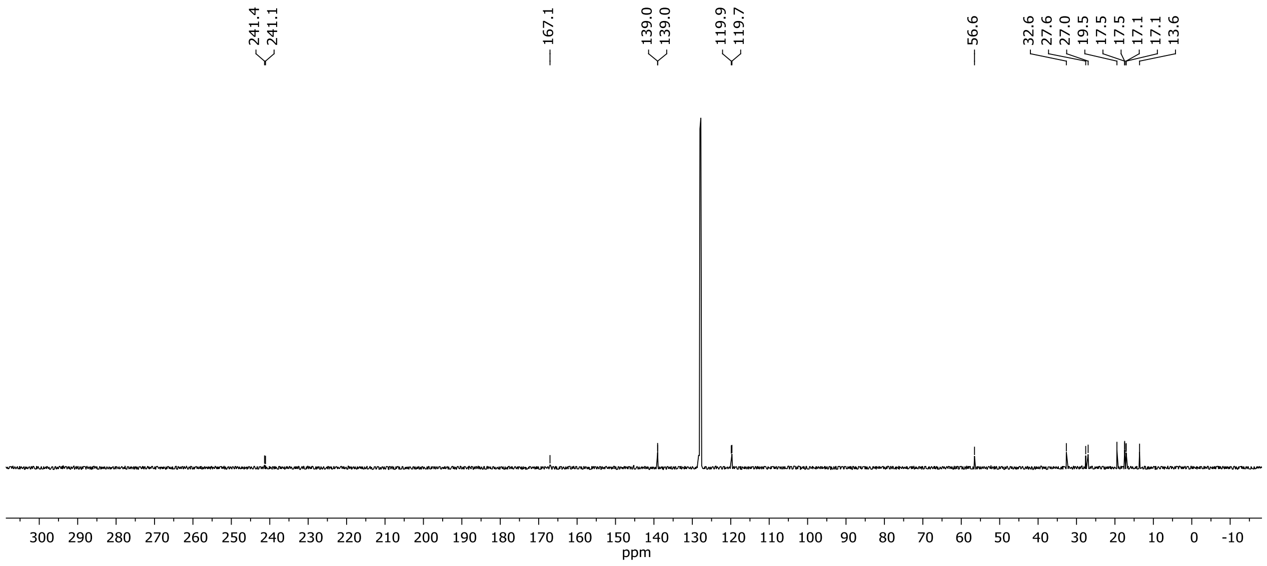

Figure S61: $^{13}C\{^{1}H\}$ NMR spectrum (benzene-d_6, 300 K, 100 MHz) of **8**.

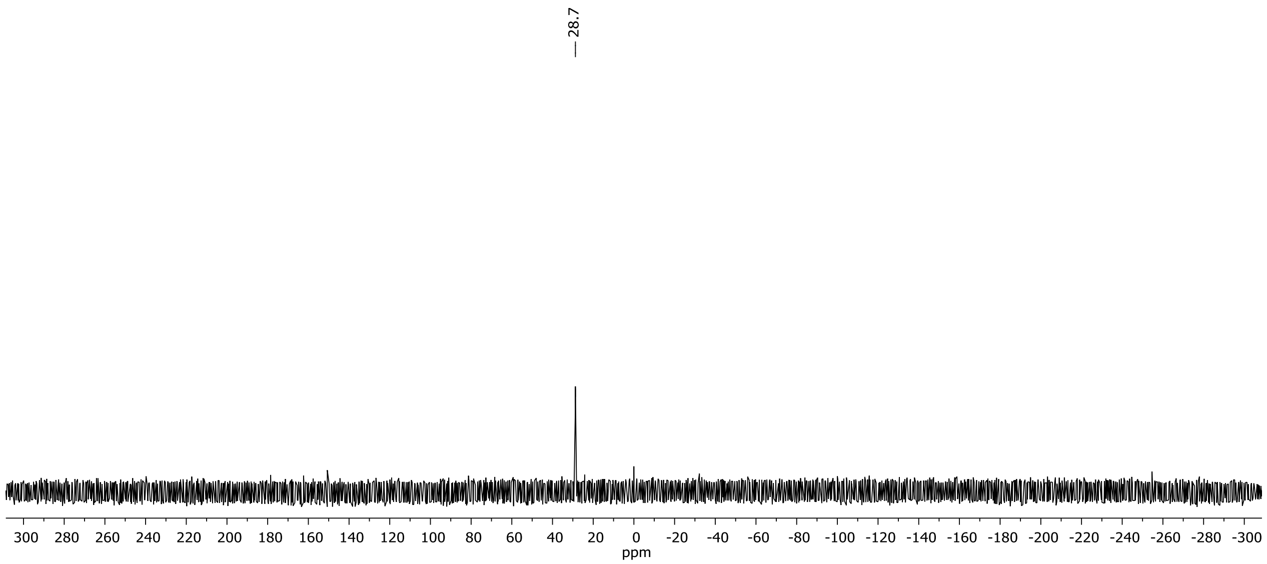

Figure S62: ^{31}P NMR spectrum (benzene-d_6, 300 K, 160 MHz) of **8**.

Synthesis of metal complexes **9-11**

Compound 9

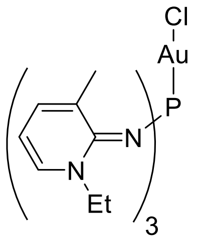

A solution of **6b** (50 mg, 0.115 mmol, 1 eq.) in toluene (2 mL) was added to a stirred suspension of $[AuCl(SMe_2)]$ (34 mg, 0.115 mmol, 1 eq.) in toluene (2 mL) at -40 °C. The solution was allowed to warm to room temperature and stirred for 16 h. All volatile compounds were removed *in vacuo* to afford **9** as brown solid in quantitative yield.

^{1}H NMR (CD_2Cl_2, 400 MHz) δ (ppm) = 7.23 (dddd, $^3J_{HH}$ = 6.9 Hz, J = 2.4 Hz, J = 1.2 Hz, J = 1.2 Hz, 3H, CH), 7.08 (m, 3H, CH), 6.06 (dd, $^3J_{HH}$ = 6.7 Hz, $^3J_{HH}$ = 6.7 Hz, 3H, 5-CH), 4.01 (q, $^3J_{HH}$ = 7.1 Hz, 6H, CH_2), 2.71 (d, J = 1.1 Hz, 9H, Ar-CH_3), 1.17 (t, $^3J_{HH}$ = 7.1 Hz, 9H, CH_2CH_3).

^{13}C{^{1}H} NMR (CD_2Cl_2, 100 MHz) δ (ppm) = 151.5 (d, $^2J_{PC}$ = 5.3 Hz, 2-C), 137.4 (s, Ar-C), 136.1 (s, Ar-C) , 131.7 (d, $^3J_{PC}$ = 3.9 Hz, 3-C), 106.6 (s, 5-C), 47.9 (s, CH_2), 23.7 (d, $^4J_{PC}$ = 3.6 Hz, Ar-CH_3), 14.8 (s, CH_2CH_3).

^{31}P NMR (CD_2Cl_2, 160 MHz) δ (ppm) = 26.6 (s).

HRMS (ESI): m/z calculated for $[C_{24}H_{34}N_6PAuCl]^+$ $(M+H)^+$ 669.19311, found 669.19640.

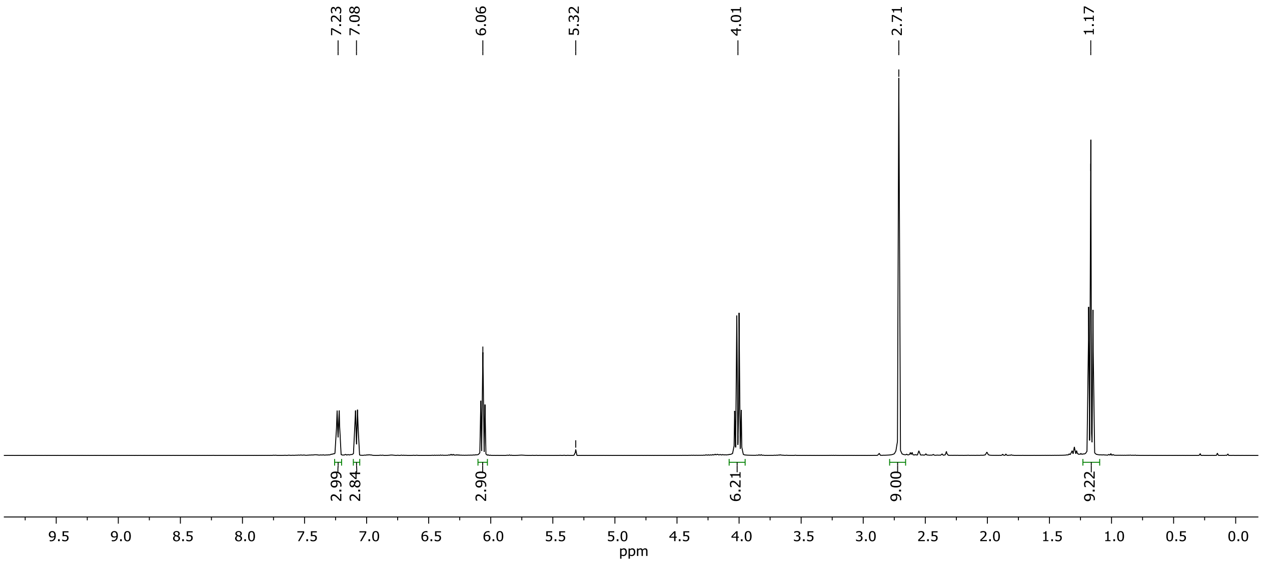

Figure S63: ^{1}H NMR spectrum (CD_2Cl_2, 300 K, 400 MHz) of **9**.

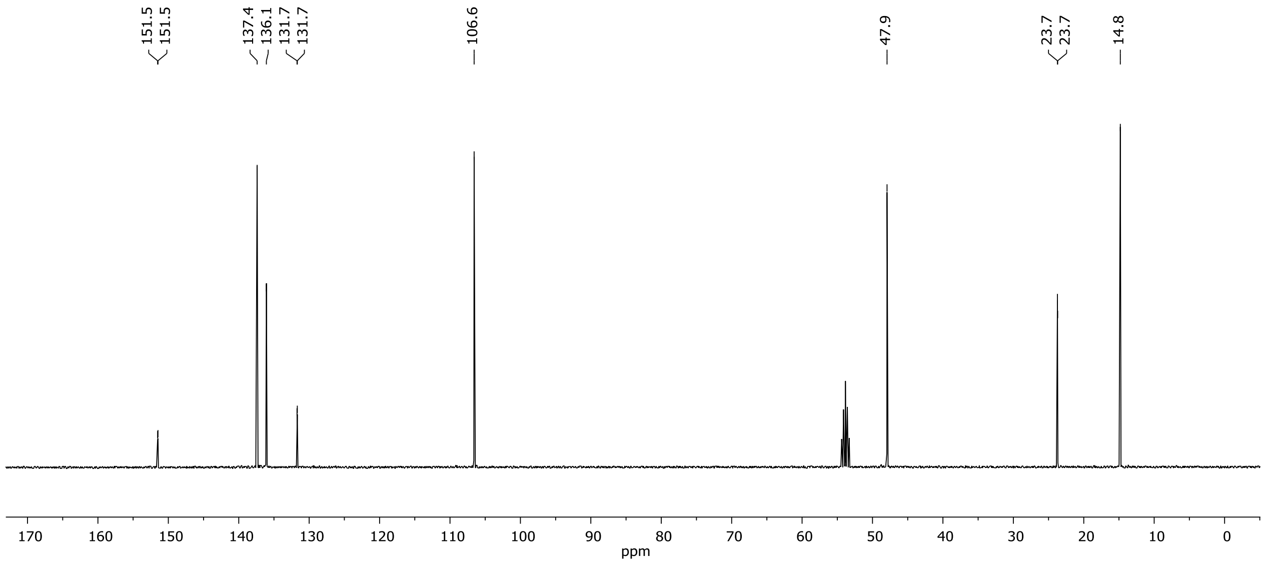

Figure S64: $^{13}C\{^1H\}$ NMR spectrum (CD_2Cl_2, 300 K, 100 MHz) of **9**.

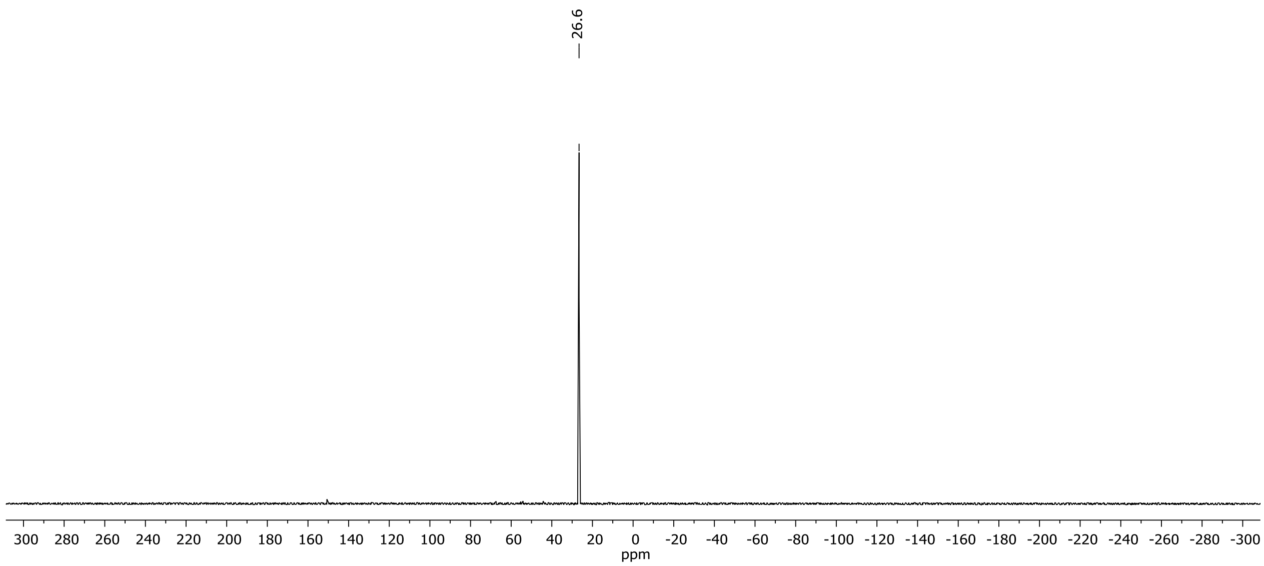

Figure S65: ^{31}P NMR spectrum (CD_2Cl_2, 300 K, 160 MHz) of **9**.

Compound 10

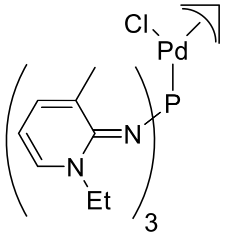

A solution of $[Pd(allyl)Cl]_2$ (15 mg, 0.041 mmol, 1 eq.) in THF (2 mL) was added dropwise to a solution of **6b** (36 mg, 0.082 mmol, 2 eq.) in THF (5 mL) at -78 °C. The mixture was allowed to warm to room temperature and all volatile compounds were removed *in vacuo* to afford **10** as yellow powder in quantitative yield.

One-Pot procedure starting from **5b**: The phosphonium salt **5b** (43 mg, 0.082 mmol, 2 eq.) and KHMDS (16 mg, 0.082 mmol, 2 eq.) were suspended in toluene (5 mL) and stirred for 16 h. A solution of $[Pd(allyl)Cl]_2$ (15 mg, 0.041 mmol, 1 eq.) in THF (5 mL)

was added dropwise at -78 °C. The reaction mixture was allowed to warm to room temperature and all volatile compounds were removed *in vacuo* to afford **10** in quantitative yield.

^{1}H NMR (CD_2Cl_2, 400 MHz) δ (ppm) = 7.19 (ddd, $^{3}J_{HH}$ = 6.9 Hz, $^{4}J_{HH}$ = 2.0 Hz, $^{4}J_{HH}$ = 2.0 Hz, 3H, CH), 6.95 (ddd, $^{3}J_{HH}$ = 6.7 Hz, $^{4}J_{HH}$ = 1.6 Hz, $^{4}J_{HH}$ = 1.6 Hz, 3H, CH), 5.92 (dd, $^{3}J_{HH}$ = 6.7 Hz, $^{3}J_{HH}$ = 6.7 Hz, 3H, 5-CH), 5.24 (m, 1H, Allyl-H), 4.28 (q, $^{3}J_{HH}$ = 7.1 Hz, 6H, CH_2), 3.21 (m, 1H, allyl-H), 2.56 (m, 9H, Ar-CH_3), 1.81 (m, 2H, Allyl-H), 1.31 (t, $^{3}J_{HH}$ = 7.1 Hz, 9H, CH_2CH_3).

^{13}C{^{1}H} NMR (CD_2Cl_2, 100 MHz) δ (ppm) = 149.9 (d, $^{2}J_{PC}$ = 4.5 Hz, 2-C), 136.0 (d, J = 1.4 Hz), 135.9 (s, Ar-C), 131.2 (d, J = 4.5 Hz), 116.5 (d, J = 9.3 Hz), 105.2, 75.2 (d, J = 50.3 Hz), 68.1, 49.4 (d, J = 10.0 Hz), 47.4, 26.0, 22.7 (d, J = 3.4 Hz), 15.2 (s, CH_2CH_3).

^{31}P NMR (CD_2Cl_2, 160 MHz) δ (ppm) = 34.8 (s).

HRMS (ESI): m/z calculated for $[C_{27}H_{38}N_6PPd:]^+$ $(M\text{-}Cl)^+$ 583.19352, found: 583.19168.

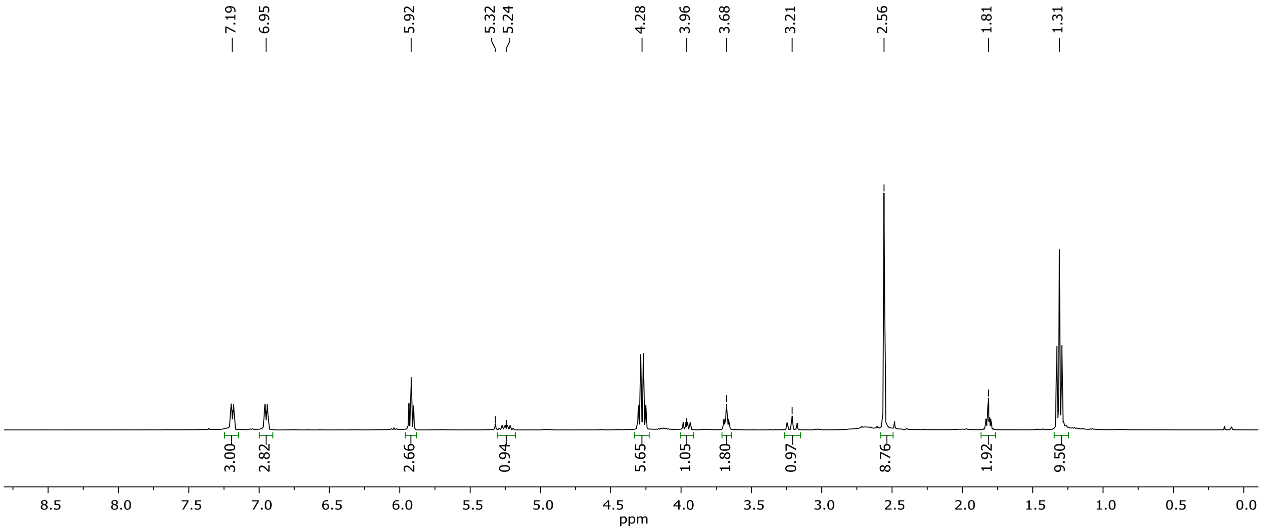

Figure S66: ^{1}H NMR spectrum (CD_2Cl_2, 300 K, 400 MHz) of **10**.

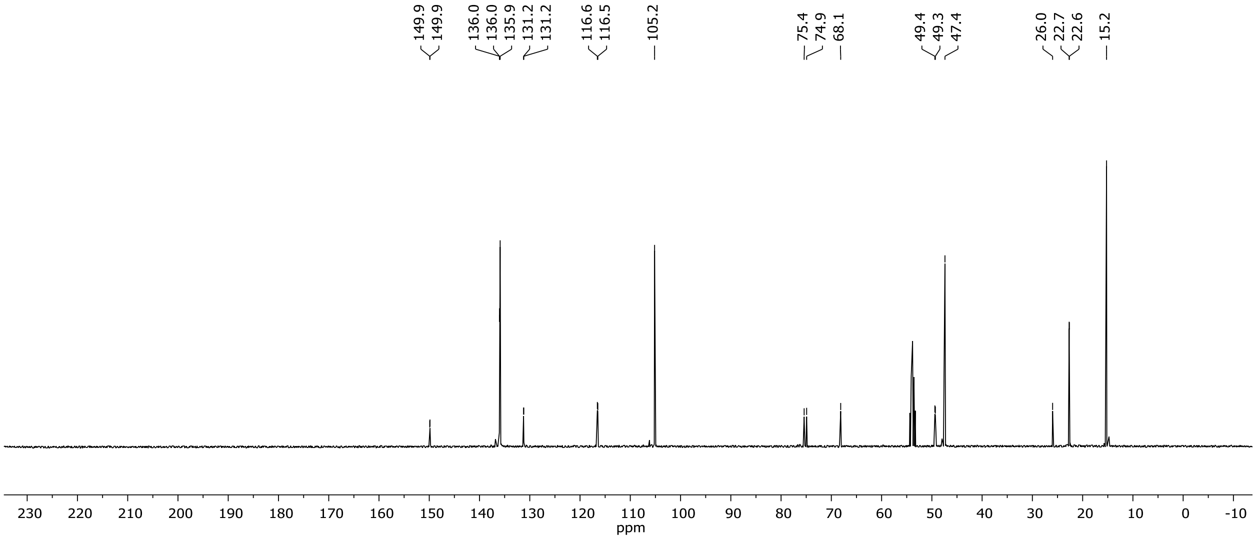

Figure S67: ^{13}C{^{1}H} NMR spectrum (CD_2Cl_2, 300 K, 100 MHz) of **10**.

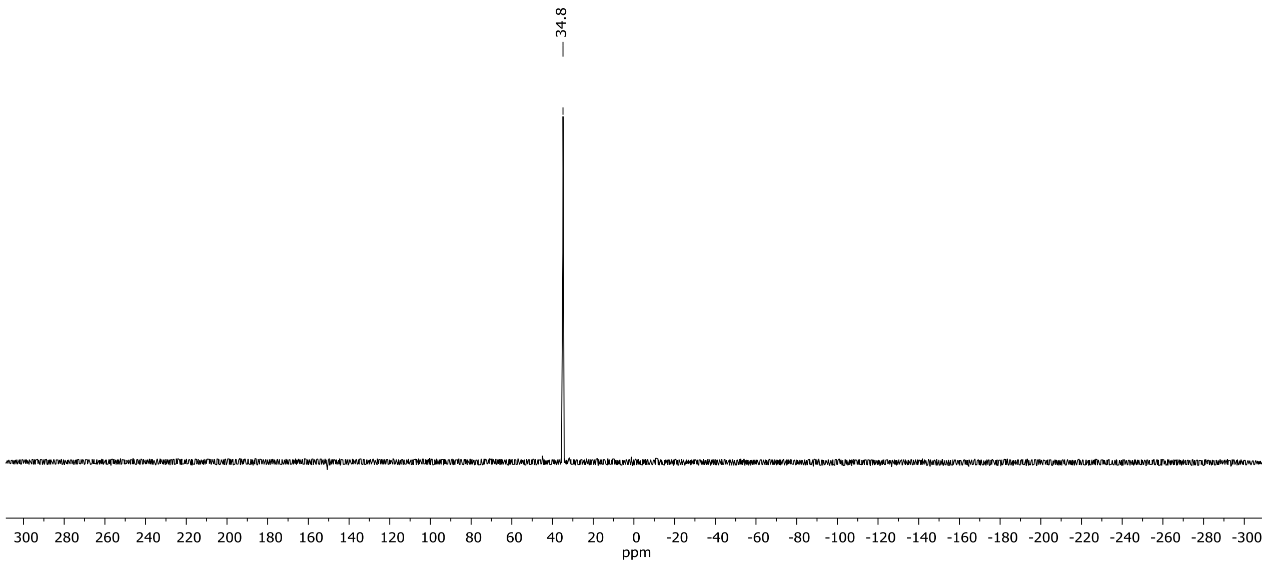

Figure S68: ^{31}P NMR spectrum (CD_2Cl_2, 300 K, 160 MHz) of **10**.

Compound 11

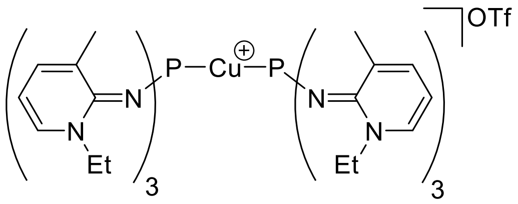

A solution of $[CuOTf]_2$•toluene (24 mg, 0.046 mmol, 1 eq.) in toluene (5 mL) was added dropwise to a solution of **6b** (80 mg, 0.184 mmol, 4 eq.) in toluene (5 mL) at -78 °C. The reaction mixture was allowed to warm to room temperature and all volatile compounds were removed *in vacuo* to afford **11** as yellow powder in quantitative yield.

^{1}H NMR (THF-d_8, 400 MHz) δ (ppm) = 7.45 (m, 3H, CH), 6.95 (d, $^{3}J_{HH}$ = 6.8 Hz, 3H, CH), 5.98 (dd, $^{3}J_{HH}$ = 6.7 Hz, $^{3}J_{HH}$ = 6.7 Hz, 3H, 5-CH), 4.01 (q, $^{3}J_{HH}$ = 7.0 Hz, 6H, CH_2), 2.57 (s, 9H, Ar-CH_3), 1.13 (t, $^{3}J_{HH}$ = 7.0 Hz, 9H, CH_2CH_3).

^{13}C{^{1}H} NMR THF-d_8, 100 MHz) δ (ppm) = 151.8 (s, 2-C), 137.6 (m, Ar-C), 130.5 (s, Ar-C), 105.9 (d, J = 11.6 Hz, Ar-C), 47.7 (s, CH_2), 24.0 (d, $^{4}J_{PC}$ = 15.8 Hz, Ar-CH_3), 14.9 (d, $^{5}J_{PC}$ = 13.1 Hz, CH_2CH_3).

^{19}F NMR (THF-d_8, 376 MHz) δ (ppm) = -78.8 (s).

^{31}P NMR (THF-d_8, 160 MHz) δ (ppm) = 40.9 (s).

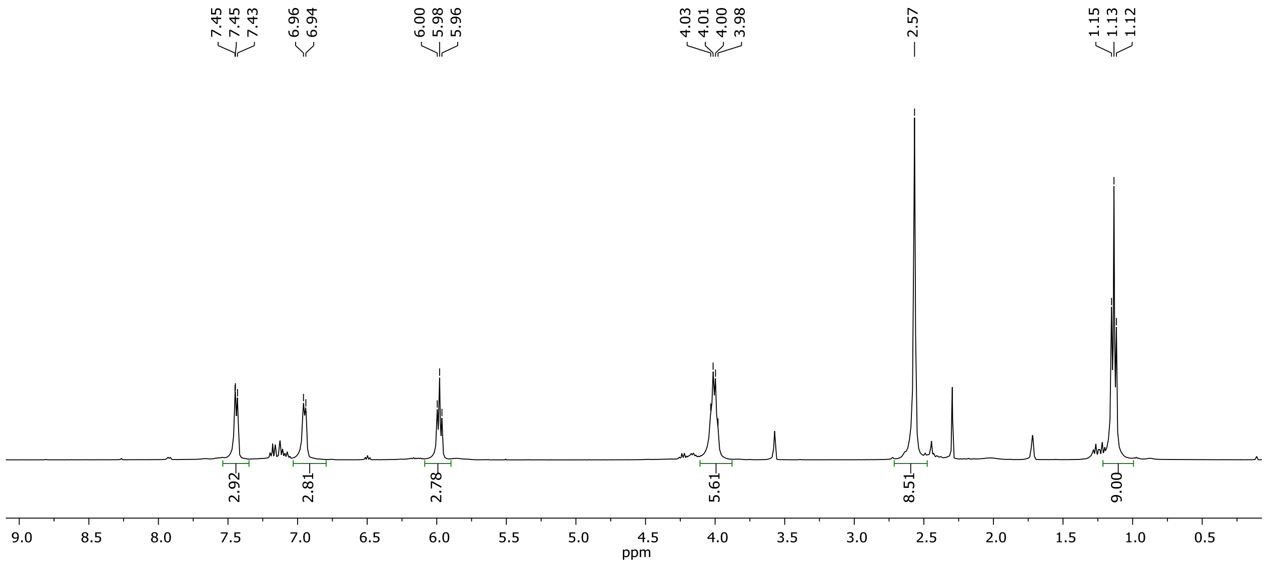

Figure S69: ^{1}H NMR spectrum (THF-d_8, 300 K, 400 MHz) of **11**.

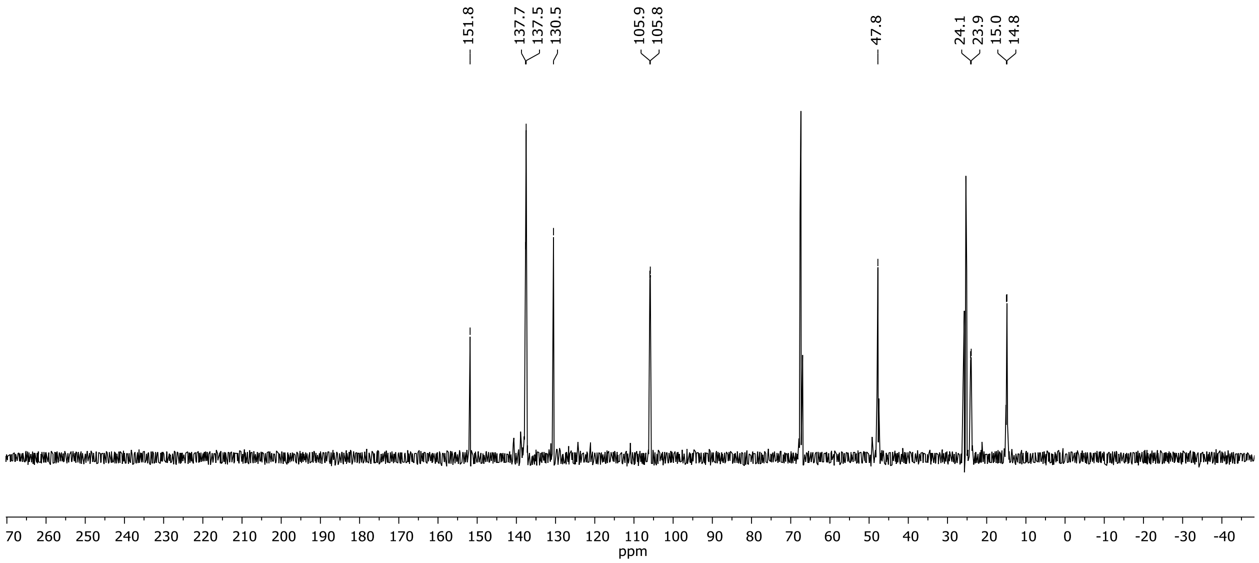

Figure S70: ^{13}C{^{1}H} NMR spectrum (THF-d_8, 300 K, 100 MHz) of **11**.

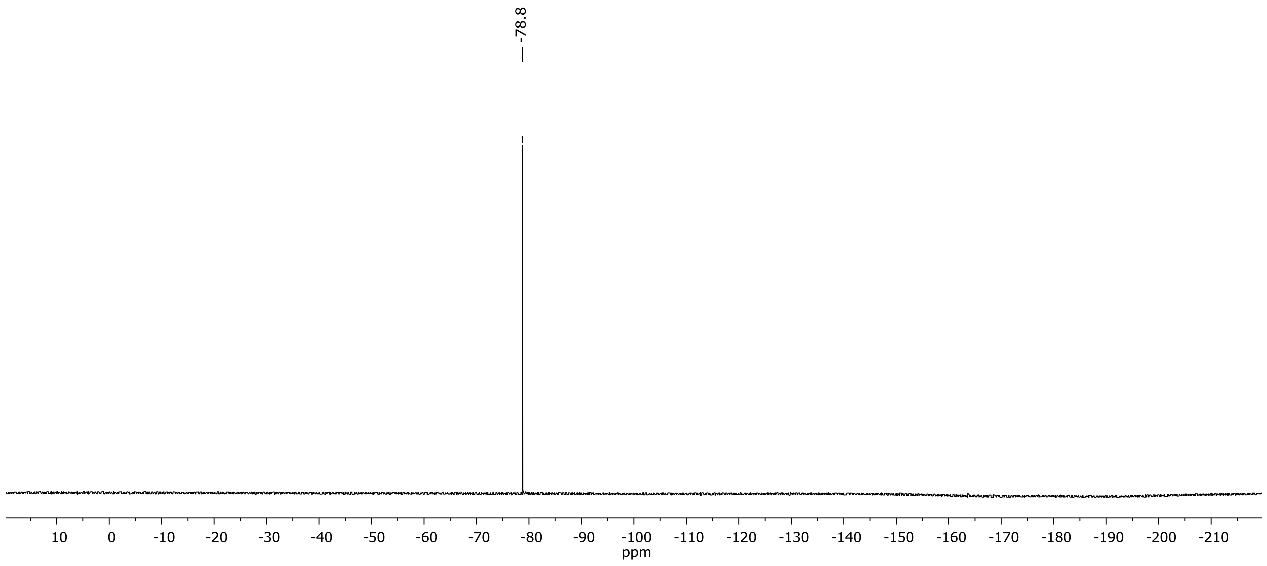

Figure S71: ^{19}F NMR spectrum (THF-d_8, 300 K, 376 MHz) of **11**.

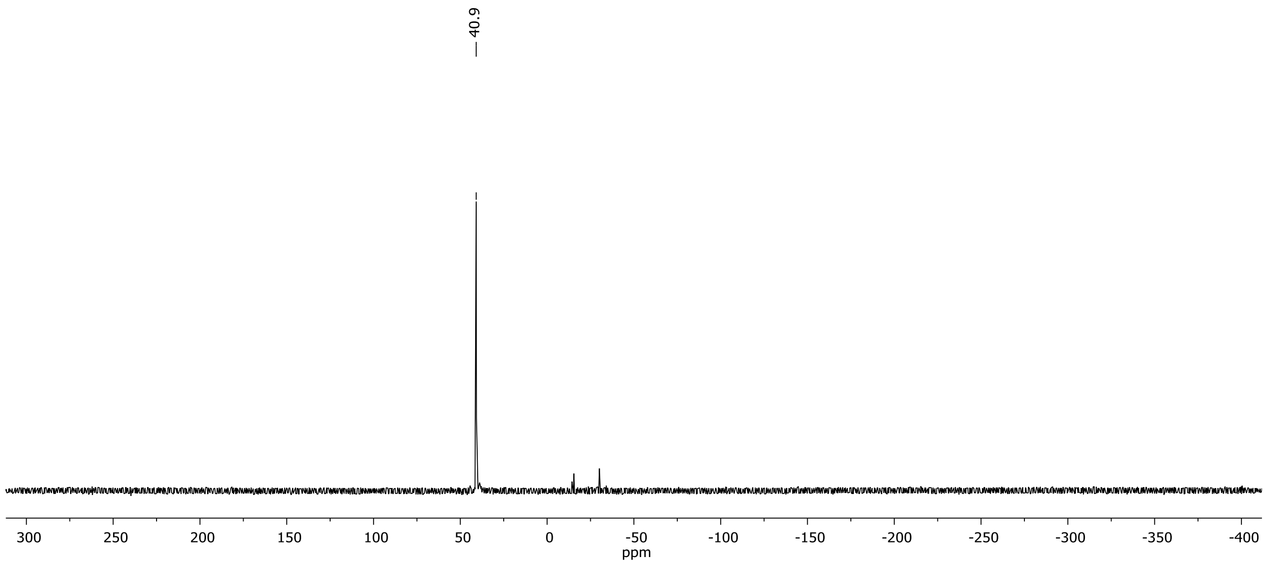

Figure S72: ^{31}P NMR spectrum (THF-d_8, 300 K, 160 MHz) of **11**.

Preparation of $[Ni(CO)_3($**PR₃**$)]$

A solution of $Ni(CO)_4$ (0.2 M in toluene, 1.5 eq.) was added to a solution of corresponding phosphine (30 mg, 1 eq.) in toluene (2 mL) After stirring for 30 min all volatile compounds were removed *in vacuo*. Either the solid residue or a solution of it in CH_2Cl_2 was analyzed by IR spectroscopy.

$[Ni(CO)_3($**2a**$)]$: **IR (CH_2Cl_2)** $\tilde{\nu}$ (A_1 CO) = 2052.1 cm^{-1}.

$[Ni(CO)_3($**2a**$)]$: **IR (solid)** $\tilde{\nu}$ (A_1 CO) = 2049.2 cm^{-1}.

$[Ni(CO)_3($**2b**$)]$: **IR (CH_2Cl_2)** $\tilde{\nu}$ (A_1 CO) = 2049.2 cm^{-1}.

$[Ni(CO)_3($**2c**$)]$: **IR (CH_2Cl_2)** $\tilde{\nu}$ (A_1 CO) = 2050.8 cm^{-1}.

$[Ni(CO)_3($**4a**$)]$: **IR (CH_2Cl_2)** $\tilde{\nu}$ (A_1 CO) = 2052.9 cm^{-1}.

$[Ni(CO)_3($**4a**$)]$: **IR (solid)** $\tilde{\nu}$ (A_1 CO) = 2051.6 cm^{-1}.

$[Ni(CO)_3($**4b**$)]$: **IR (CH_2Cl_2)** $\tilde{\nu}$ (A_1 CO) = 2051.3 cm^{-1}.

$[Ni(CO)_3($**4c**$)]$: **IR (CH_2Cl_2)** $\tilde{\nu}$ (A_1 CO) = 2052.2 cm^{-1}.

$[Ni(CO)_3($**4d**$)]$: **IR (CH_2Cl_2)** $\tilde{\nu}$ (A_1 CO) = 2051.3 cm^{-1}.

$[Ni(CO)_3($**4d**$)]$: **IR (solid)** $\tilde{\nu}$ (A_1 CO) = 2049.6 cm^{-1}.

$[Ni(CO)_3($**6a**$)]$: **IR (CH_2Cl_2)** $\tilde{\nu}$ (A_1 CO) = 2041.2 cm^{-1}.

$[Ni(CO)_3($**6b**$)]$: **IR (CH_2Cl_2)** $\tilde{\nu}$ (A_1 CO) = 2040.7 cm^{-1}.

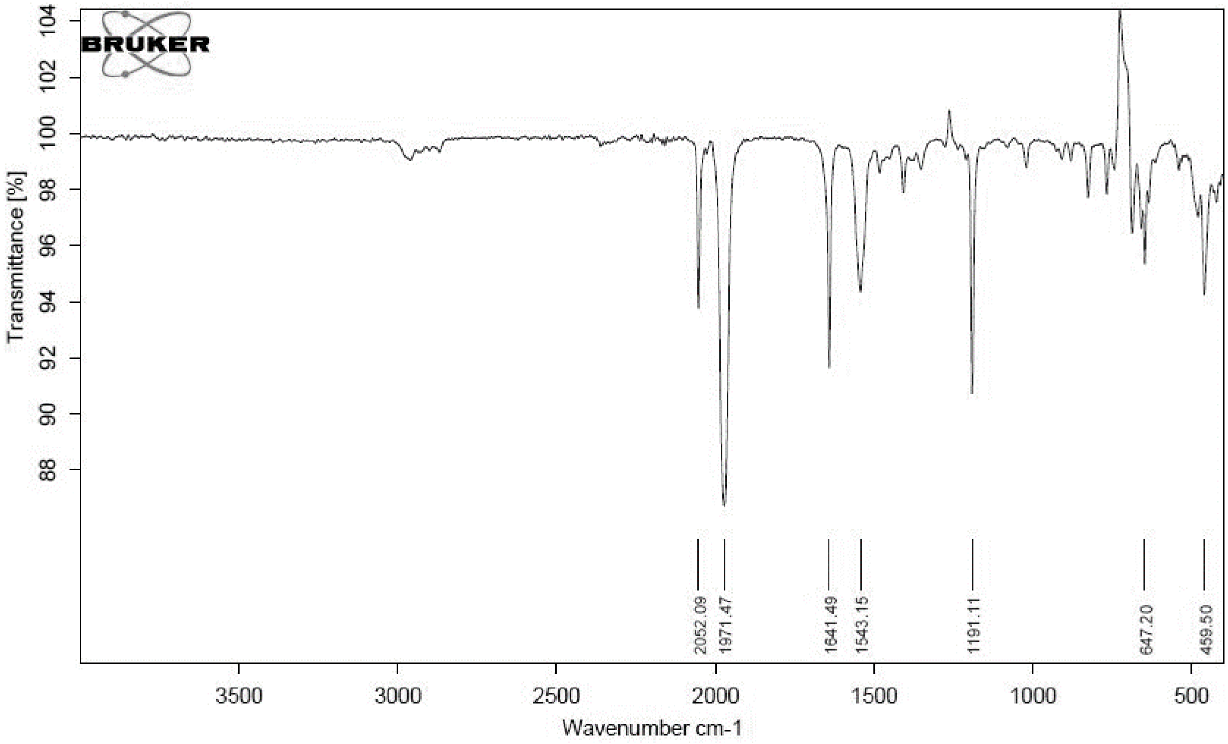

Figure S73: FT-IR spectrum of $[Ni(CO)_3($**2a**$)]$ (in CH_2Cl_2).

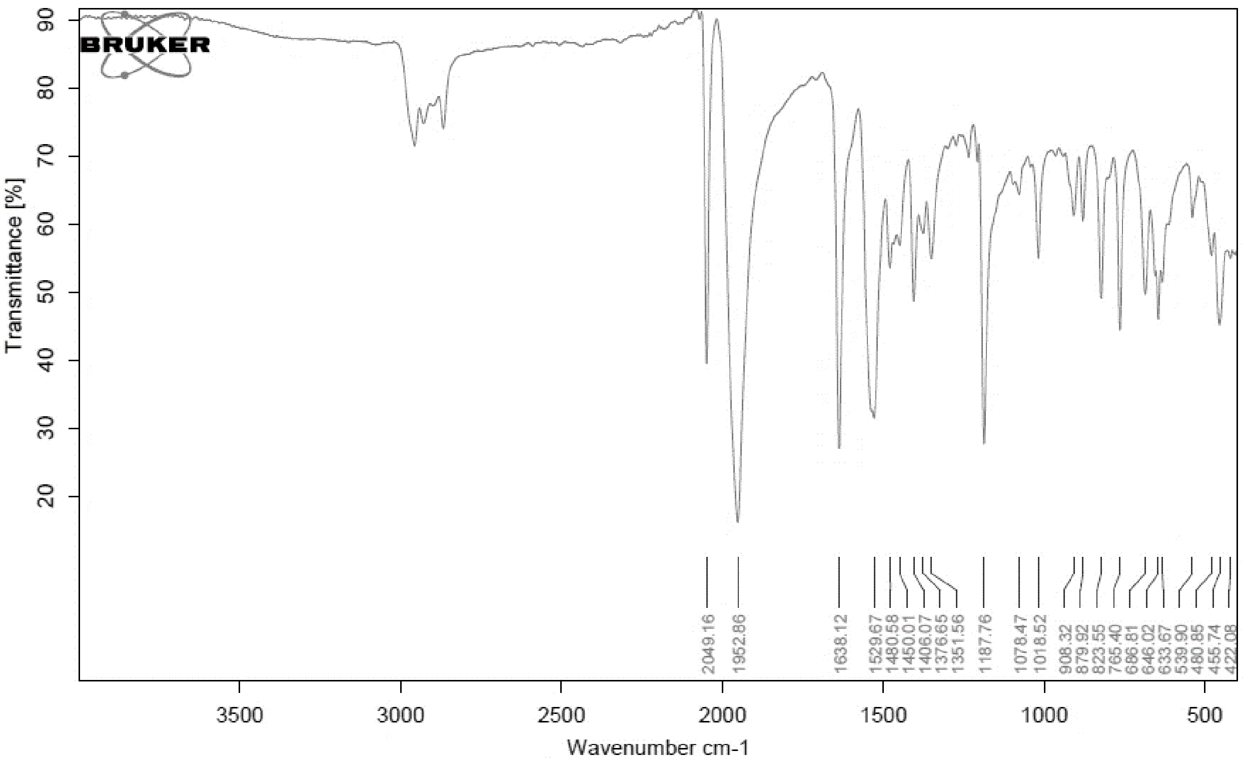

Figure S74: FT-IR spectrum of [Ni(CO)$_3$(**2a**)] (solid).

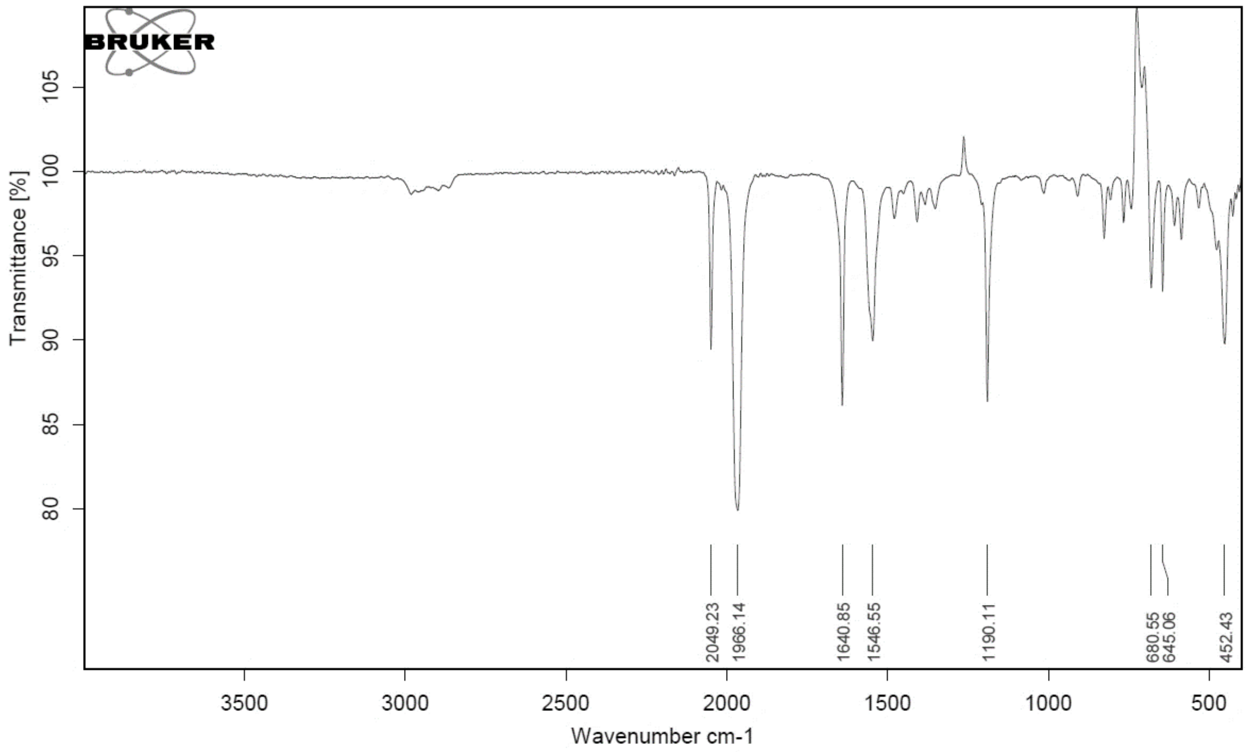

Figure S75: FT-IR spectrum of [Ni(CO)$_3$(**2b**)] (in CH_2Cl_2).

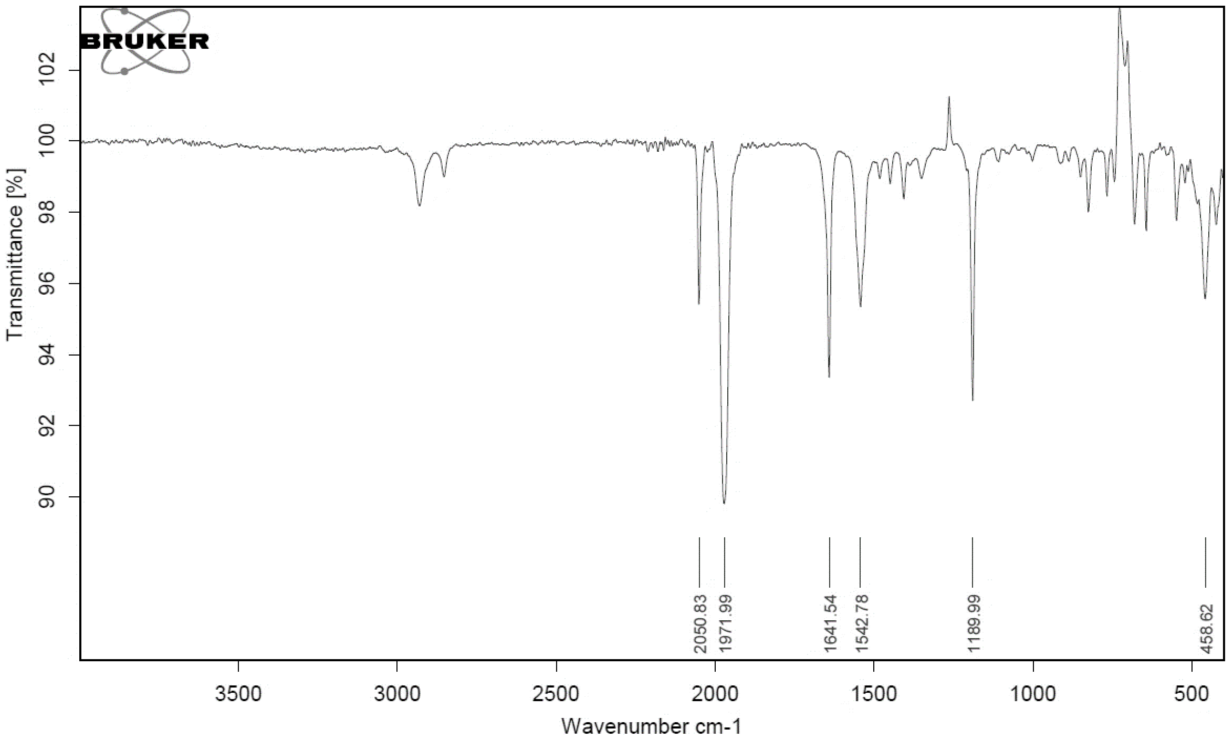

Figure S76: FT-IR spectrum of [Ni(CO)$_3$(**2c**)] (in CH_2Cl_2).

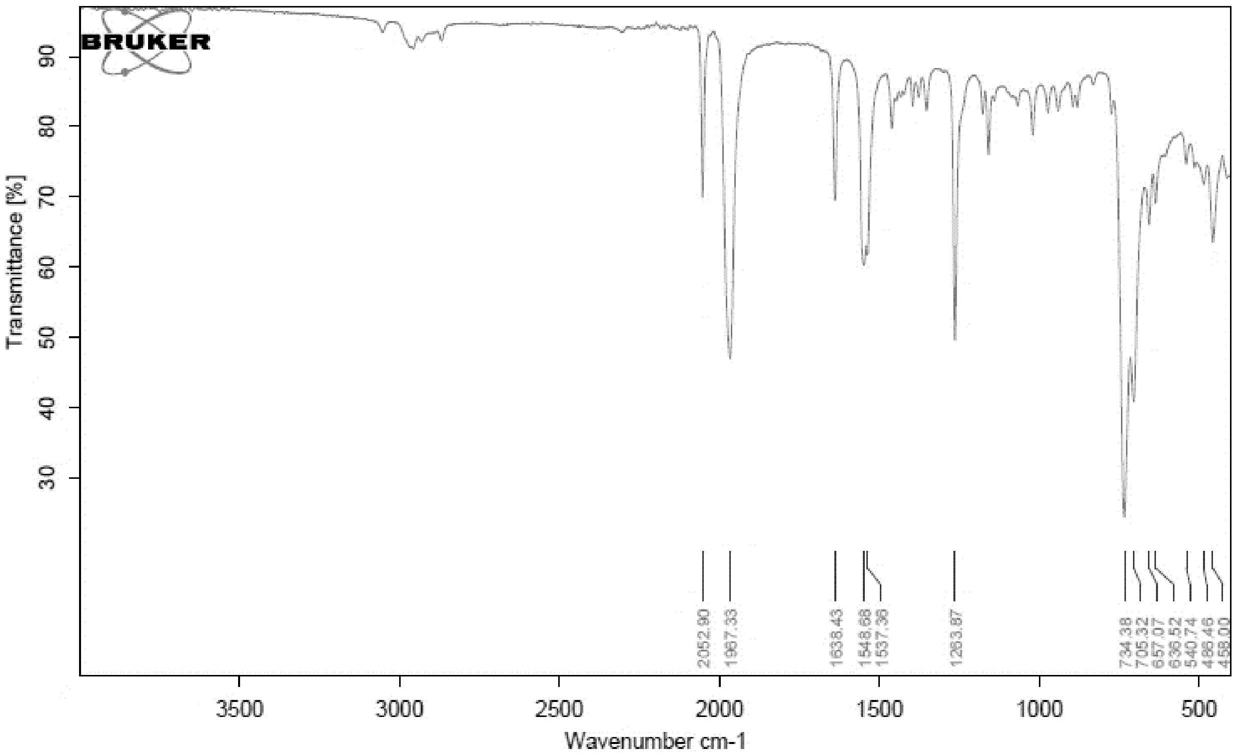

Figure S77: FT-IR spectrum of [Ni(CO)$_3$(**4a**)] (in CH_2Cl_2).

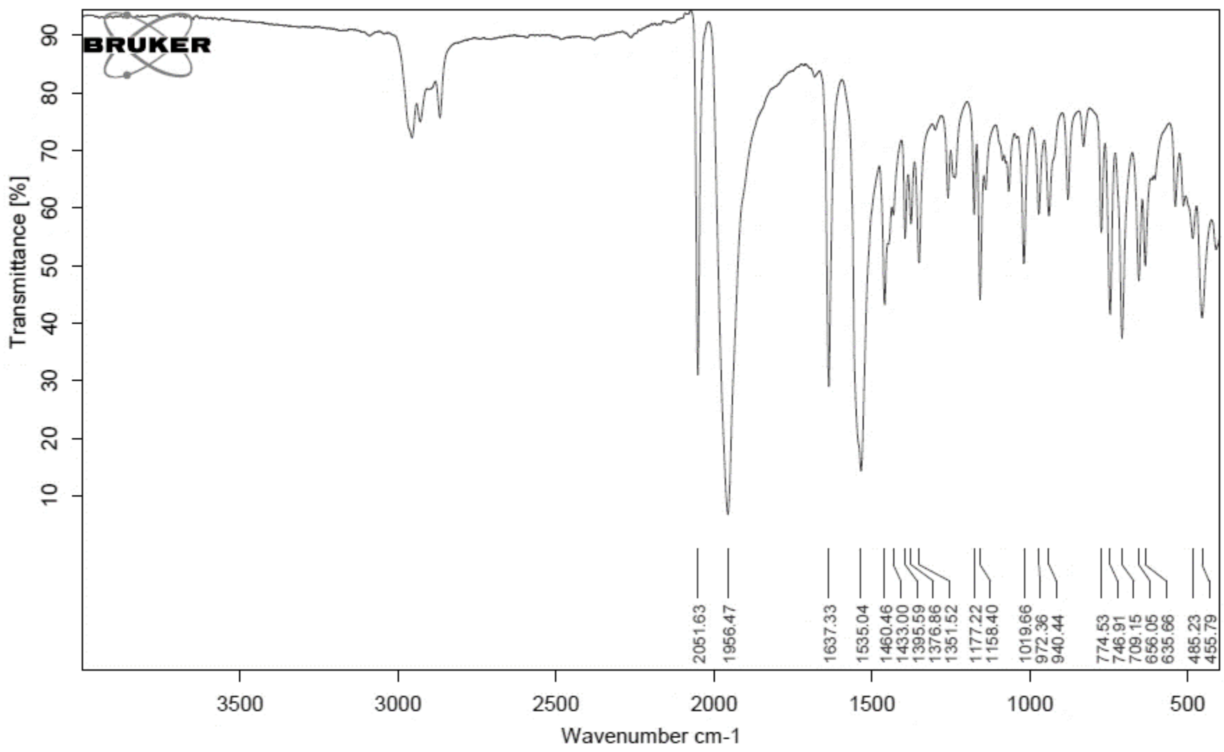

Figure S78: FT-IR spectrum of [Ni(CO)$_3$(**4a**)] (solid).

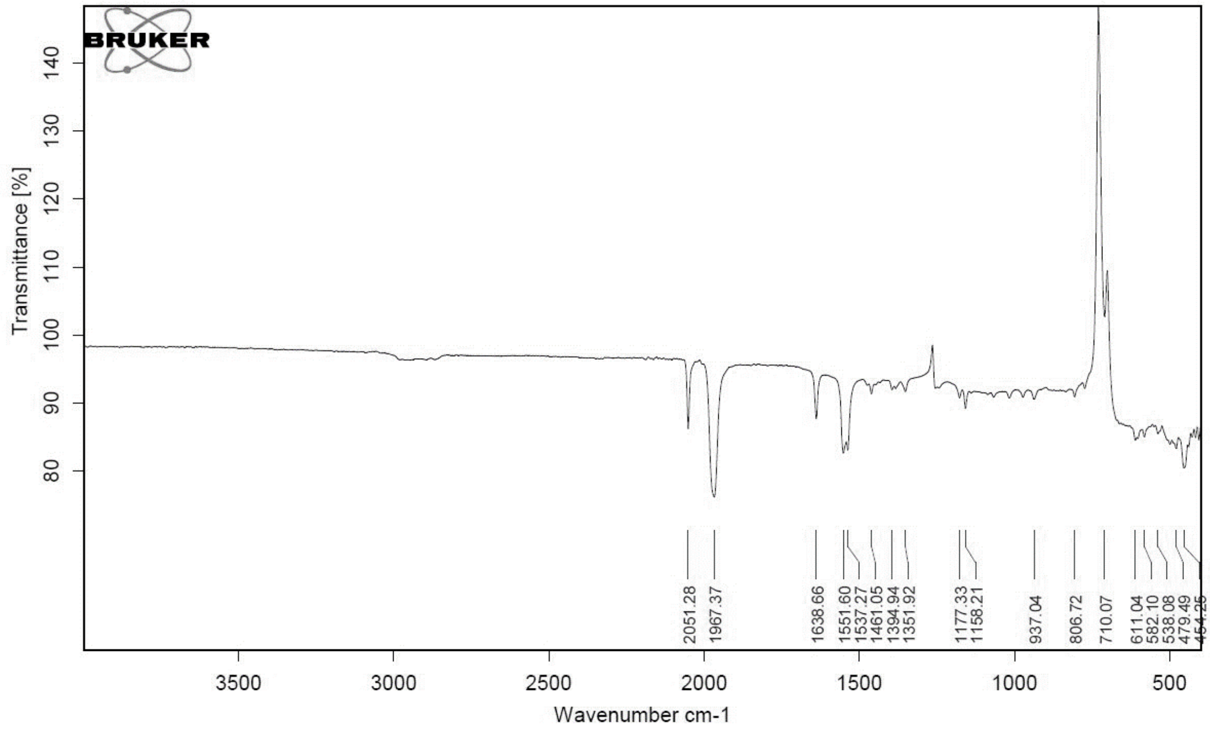

Figure S79: FT-IR spectrum of [Ni(CO)$_3$(**4b**)] (in CH_2Cl_2).

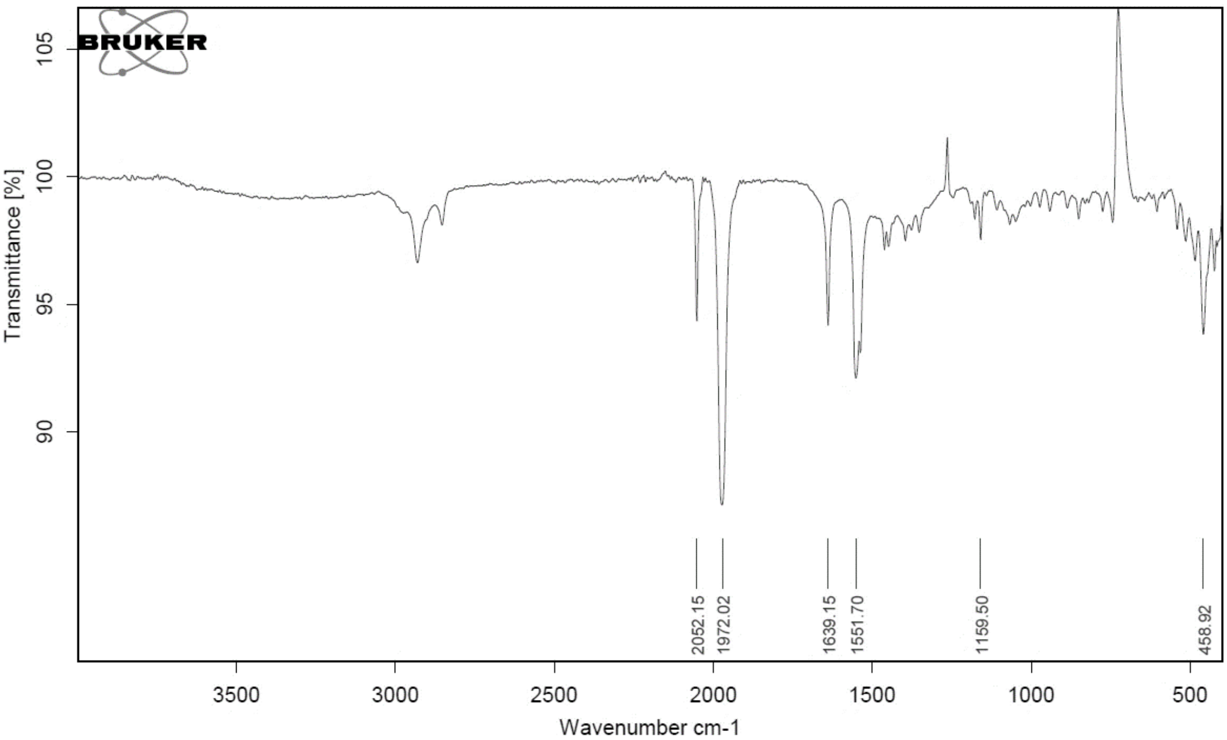

Figure S80: FT-IR spectrum of [Ni(CO)$_3$(**4c**)] (in CH_2Cl_2).

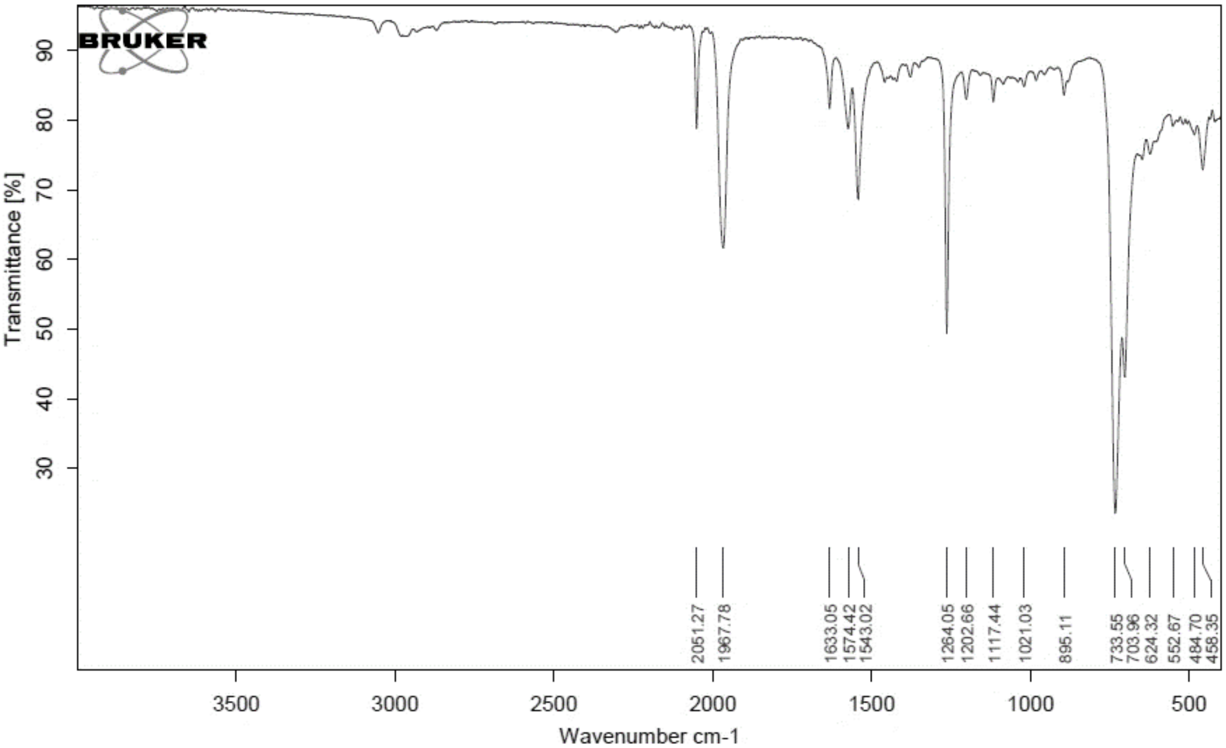

Figure S81: FT-IR spectrum of [Ni(CO)$_3$(**4d**)] (in CH_2Cl_2).

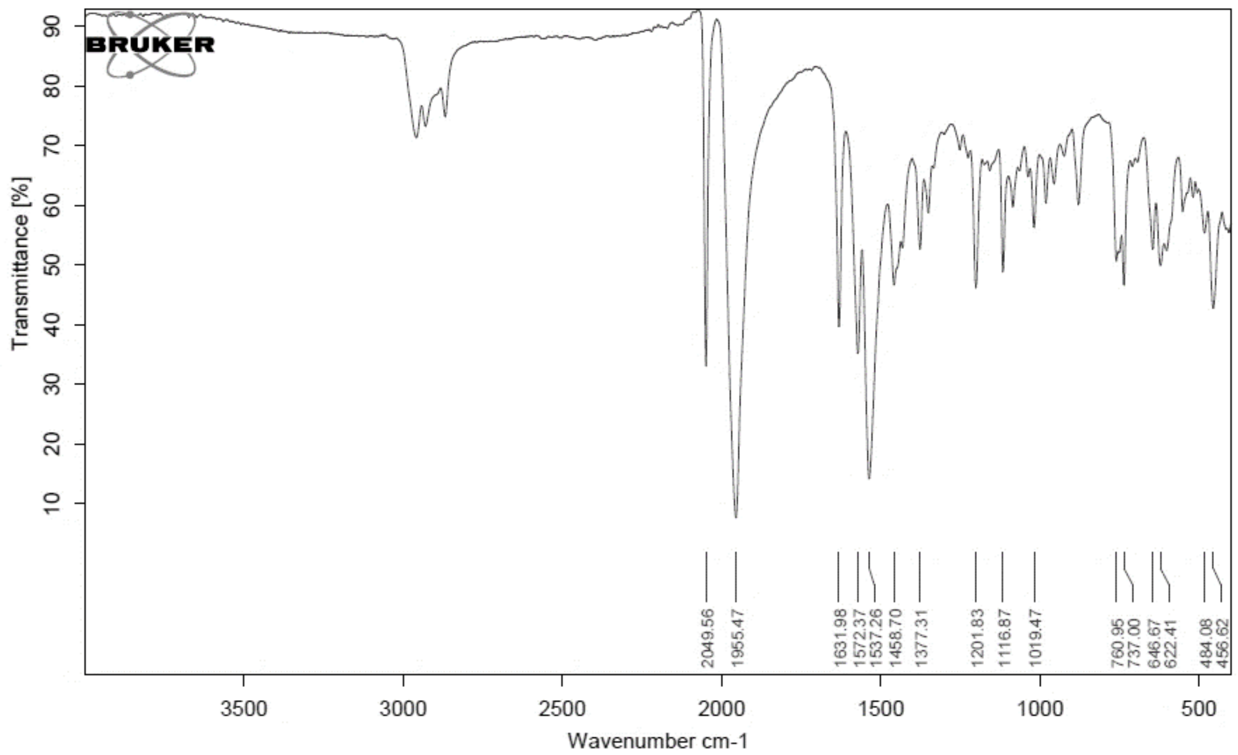

Figure S82: FT-IR spectrum of [Ni(CO)$_3$(**4d**)] (solid).

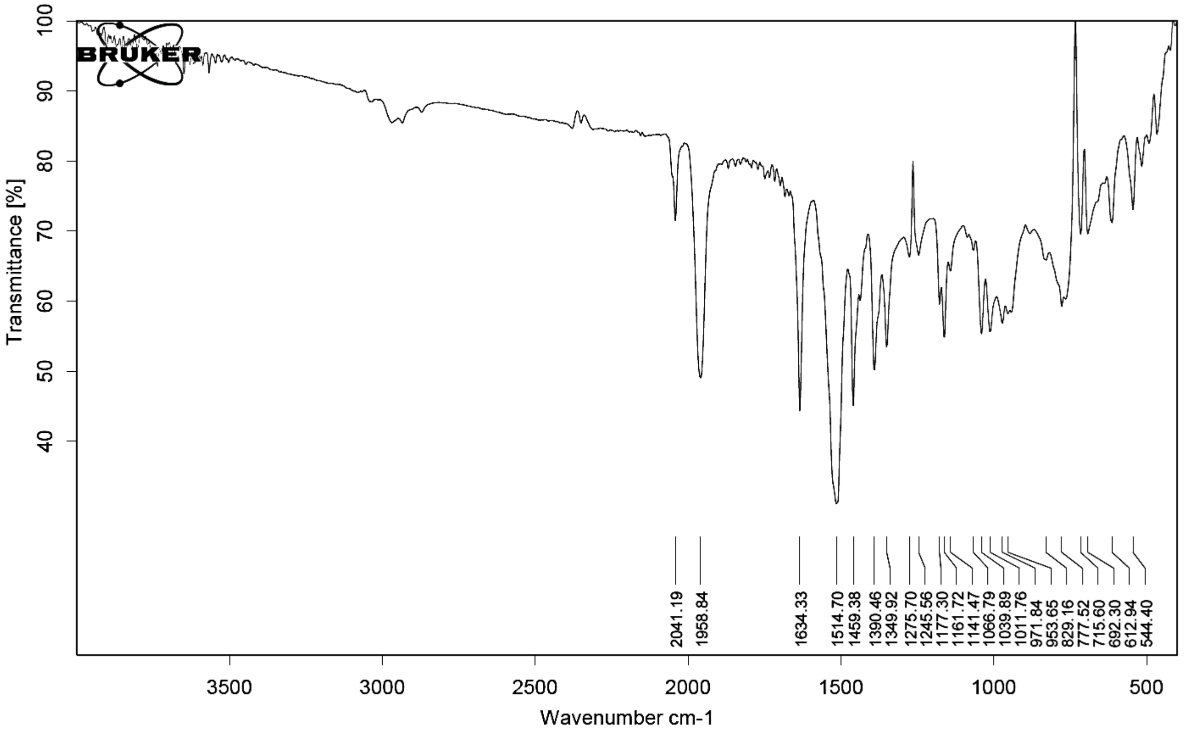

Figure S83: FT-IR spectrum of [Ni(CO)$_3$(**6a**)] (in CH_2Cl_2).

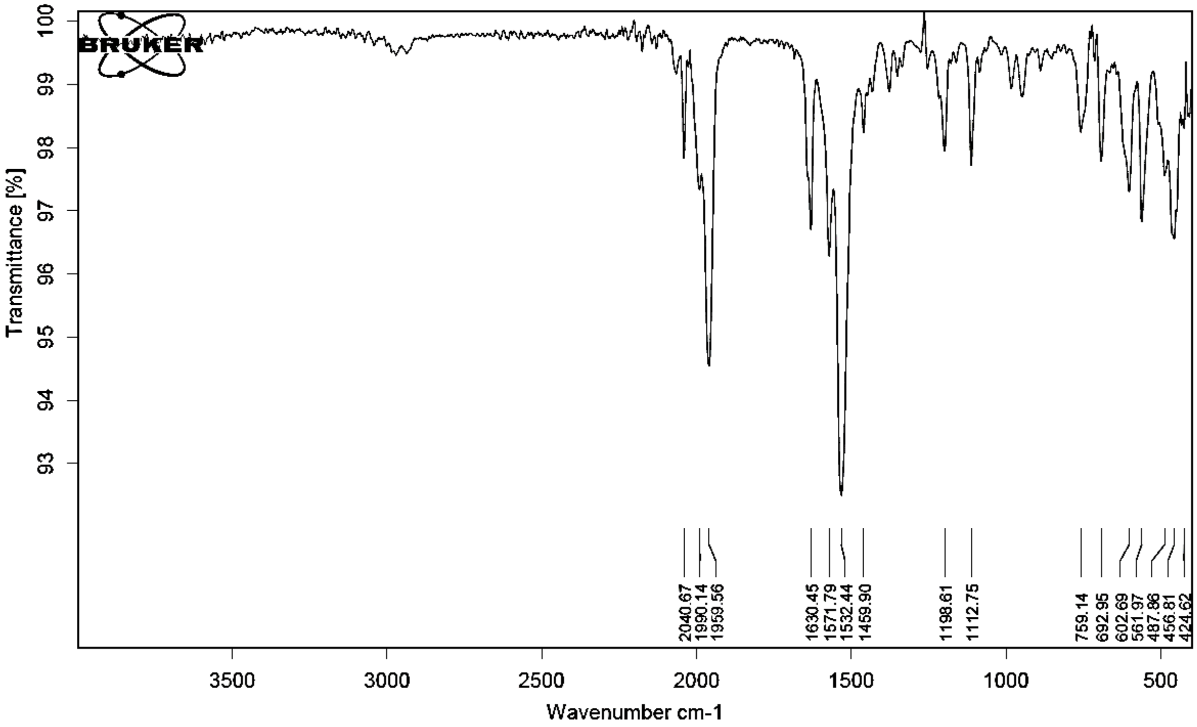

Figure S84: FT-IR spectrum of [Ni(CO)$_3$(**6b**)] (in CH_2Cl_2).

X-ray Diffraction Studies

General: Single-crystal X-ray diffraction data were collected on a Bruker AXS detector using Mo-K_α radiation (λ = 0.71073 Å). Crystals were selected under oil, mounted on nylon loops and then immediately placed in a cold stream of N_2 on a diffractometer. Using Olex2,[9] the structures were solved with the Superflip[10] structure solution program using Charge Flipping and refined with the ShelXL[11] refinement package using Least Squares minimisation.

Crystallographic data have been deposited with the Cambridge Crystallographic Data Centre as supplementary publication no. CCDC-1950838 (**2d**), CCDC-1950839 (**6b**), CCDC-1950840 (**7**), CCDC-1950841 (**8**), CCDC-1950842 (**9**) and CCDC-1950843 (**10**). These data can be obtained free of charge via www.ccdc.cam.uk/data_request/cif (or from the CCDC, 12 Union Road, Cambridge CB2 1EZ, UK; fax: (+44) 1223-336-033; or deposit@ccdc.cam.ac.uk).

Single-crystal X-ray structure analysis of 2d:

Single crystals were obtained by slow cooling of a saturated *n*-hexane solution of **2d**. A single-crystal X-ray structure analysis revealed that **2d** crystallizes in the monoclinic space group $P2_1/n$. The asymmetric unit contains one molecule of **2d**.

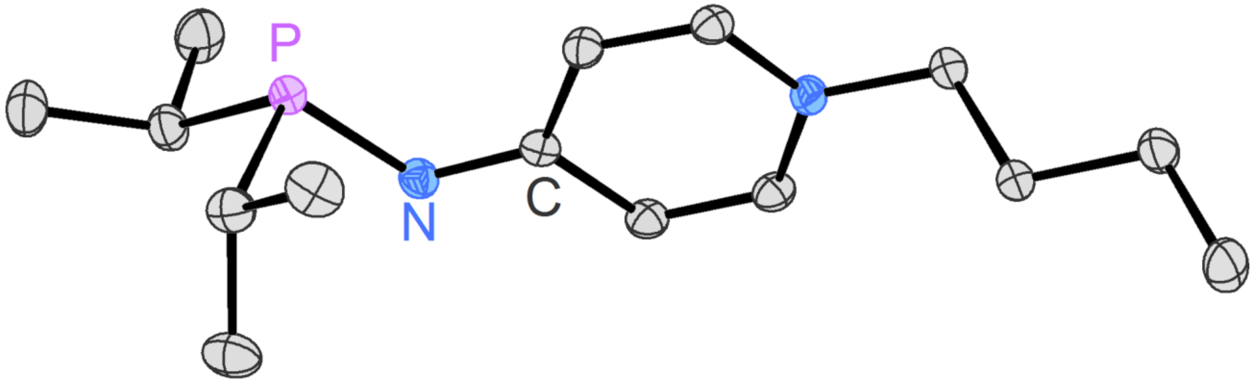

Figure S85: Molecular view of **2d** in the solid state with thermal ellipsoid plot at the 50% levels of probability. Hydrogen atoms are omitted for clarity.

Table S1. Crystal data and structure refinement for **2d**.

CCDC number	1950838	ρ_{calc}mg/mm^3	1.063
Empirical formula	$C_{15}H_{27}N_2P$	m/mm^{-1}	0.153
Formula weight	266.35	F(000)	584.0
Temperature/K	100(2)	Crystal size/mm^3	0.43 × 0.15 × 0.06
Crystal system	monoclinic	Radiation	MoKα (λ = 0.71073)
Space group	$P2_1/n$	2Θ range for data collection	6.984 to 56.564°
a/Å	7.8785(6)	Index ranges	$-10 \leq h \leq 10$, $-29 \leq k \leq 29$, $-12 \leq l \leq 12$
b/Å	22.0789(17)	Reflections collected	18248
c/Å	9.6088(8)	Independent reflections	4107 [R_{int} = 0.0285, R_{sigma} = 0.0241]
α/°	90	Data/restraints/parameters	4107/0/168
β/°	95.2112(13)	Goodness-of-fit on F^2	1.051
γ/°	90	Final R indexes [I>=2σ (I)]	R_1 = 0.0402, wR_2 = 0.1058
Volume/Å^3	1664.5(2)	Final R indexes [all data]	R_1 = 0.0484, wR_2 = 0.1120
Z	4	Largest diff. peak/hole / e Å^{-3}	0.47/-0.23

Single-crystal X-ray structure analysis of 6b:

Single crystals were obtained by slow cooling of a saturated *n*-hexane solution of **6b**. A single-crystal X-ray structure analysis revealed that **6b** crystallizes in the trigonal space group $P\overline{3}$. The asymmetric unit contains ⅓ molecule of **6b**. The phosphorus atom is dosordered over two positions with (95:5) relative occupancy.

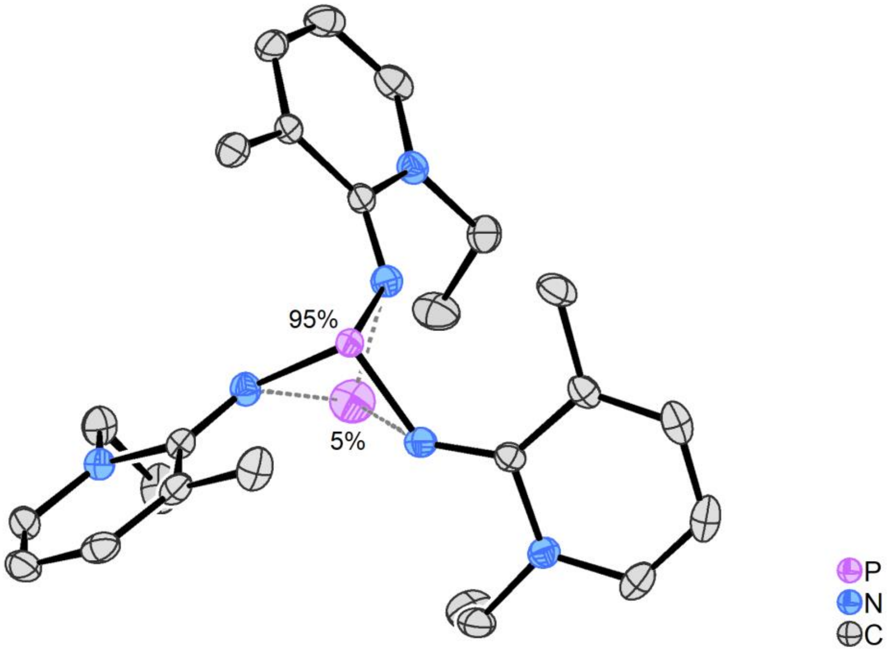

Figure S86: Molecular view of the asymetric unit of **6b** in the solid state with thermal ellipsoid plot at the 50% levels of probability. Hydrogen atoms are omitted for clarity.

Table S2. Crystal data and structure refinement for **6b**.

CCDC number	1950839	ρ_{calc}mg/mm^3	1.224
Empirical formula	$C_{24}H_{33}N_6P$	m/mm^{-1}	0.139
Formula weight	436.54	F(000)	468.0
Temperature/K	100(2)	Crystal size/mm^3	0.6 × 0.389 × 0.231
Crystal system	trigonal	Radiation	MoKα (λ = 0.71073)
Space group	*P*-3	2Θ range for data collection	3.498 to 56.598°
a/Å	13.4462(4)	Index ranges	$-17 \le h \le 17$, $-17 \le k \le 17$, $-10 \le l \le 10$
b/Å	13.4462(4)	Reflections collected	18395
c/Å	7.5640(2)	Independent reflections	1967 [R_{int} = 0.0429, R_{sigma} = 0.0184]
α/°	90	Data/restraints/parameters	1967/0/100
β/°	90	Goodness-of-fit on F^2	1.124
γ/°	120	Final R indexes [I>=2σ (I)]	R_1 = 0.0445, wR_2 = 0.1172
Volume/Å^3	1184.35(8)	Final R indexes [all data]	R_1 = 0.0460, wR_2 = 0.1185
Z	2	Largest diff. peak/hole / e Å^{-3}	0.57/-0.21

Single-crystal X-ray structure analysis of 7:

Single crystals were obtained by slow cooling of a saturated THF solution of **7**. A single-crystal X-ray structure analysis revealed that **7** crystallizes in the monoclinic space group $P2_1/c$. The asymmetric unit contains one molecule of **7**.

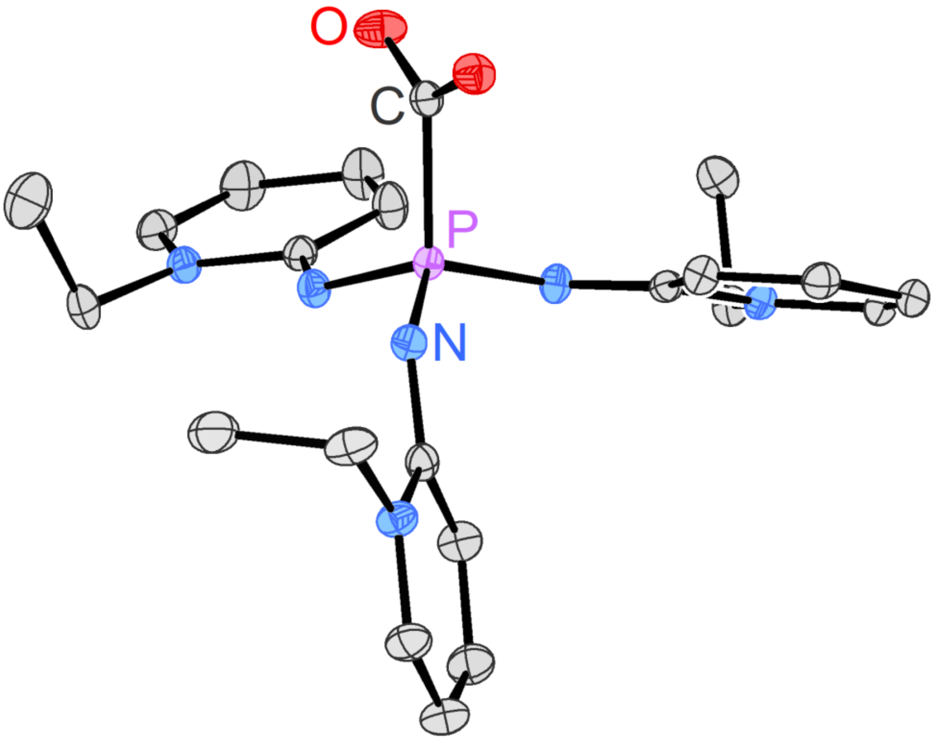

Figure S87: Molecular view of **7** in the solid state with thermal ellipsoid plot at the 50% levels of probability. Hydrogen atoms are omitted for clarity.

Table S3. Crystal data and structure refinement for **7**.

CCDC number	1950840	ρ_{calc}mg/mm^3	1.284
Empirical formula	$C_{22}H_{27}N_6O_2P$	m/mm^{-1}	0.152
Formula weight	438.46	F(000)	928.0
Temperature/K	100(2)	Crystal size/mm^3	0.6 × 0.38 × 0.258
Crystal system	monoclinic	Radiation	MoKα (λ = 0.71073)
Space group	$P2_1/c$	2Θ range for data collection	3.5 to 56.624
a/Å	8.2850(3)	Index ranges	$-11 \leq h \leq 11$, $-20 \leq k \leq 20$, $-24 \leq l \leq 24$
b/Å	15.3902(6)	Reflections collected	34884
c/Å	18.2294(7)	Independent reflections	5628 [R_{int} = 0.0262, R_{sigma} = 0.0163]
α/°	90	Data/restraints/parameters	5628/0/283
β/°	102.664(2)	Goodness-of-fit on F^2	1.030
γ/°	90	Final R indexes [I>=2σ (I)]	R_1 = 0.0345, wR_2 = 0.0902
Volume/Å^3	2267.84(15)	Final R indexes [all data]	R_1 = 0.0372, wR_2 = 0.0925
Z	4	Largest diff. peak/hole / e Å^{-3}	0.42/-0.34

Single-crystal X-ray structure analysis of 8:

Single crystals were obtained by slow diffusion of *n*-hexane into a solution of **8** in THF. A single-crystal X-ray structure analysis revealed that **2c** crystallizes in the monoclinic space group *C2/c*. The asymmetric unit contains one molecule of **8**. The n-butyl group is disordered over two position with relative occupancies of 69:31. The PLATON SQUEEZE program was used to treat two severely disordered THF solvate molecules.

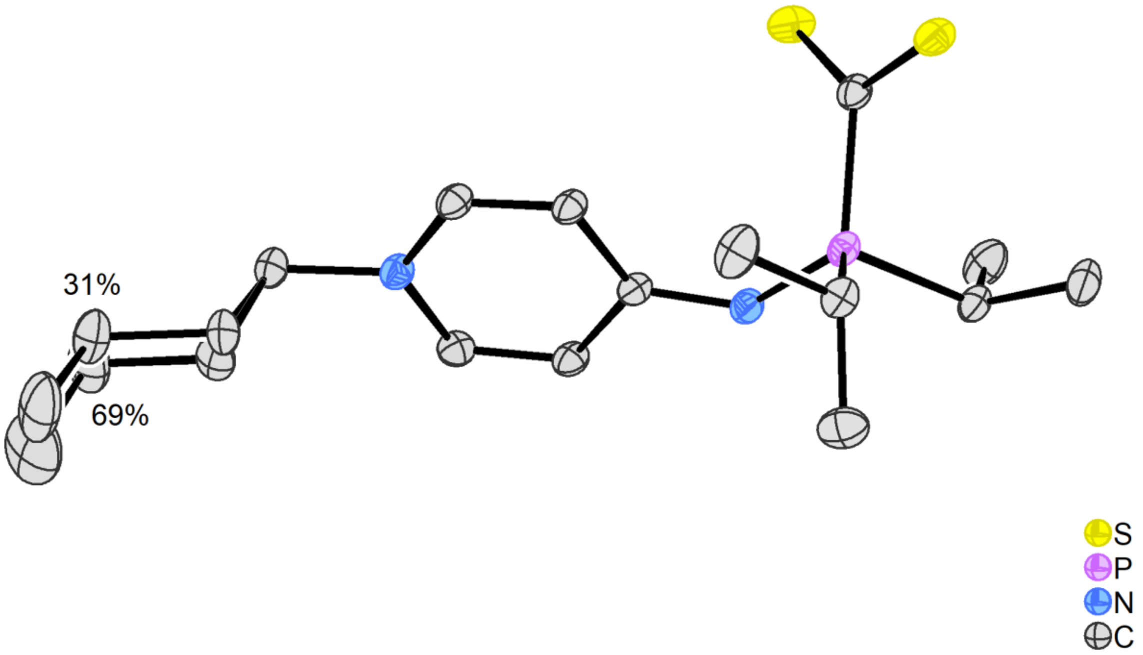

Figure S88: Molecular view of **8** in the solid state with thermal ellipsoid plot at the 50% levels of probability. Hydrogen atoms are omitted for clarity.

Table S4. Crystal data and structure refinement for **8**.

CCDC number	1950841	ρ_{calc}mg/mm^3	0.880
Empirical formula	$C_{16}H_{27}N_2PS_2$	m/mm^{-1}	0.265
Formula weight	342.48	F(000)	1472.0
Temperature/K	100(2)	Crystal size/mm^3	0.41 × 0.13 × 0.12
Crystal system	monoclinic	Radiation	MoKα (λ = 0.71073)
Space group	*C2/c*	2Θ range for data collection	6.716 to 59.208°
a/Å	25.0433(5)	Index ranges	-34 ≤ h ≤ 34, -12 ≤ k ≤ 12, -33 ≤ l ≤ 33
b/Å	8.7952(2)	Reflections collected	41377
c/Å	24.3543(5)	Independent reflections	7252 [R_{int} = 0.0380, R_{sigma} = 0.0266]
α/°	90	Data/restraints/parameters	7252/0/224
β/°	105.5629(10)	Goodness-of-fit on F^2	1.075
γ/°	90	Final R indexes [I>=2σ (I)]	R_1 = 0.0403, wR_2 = 0.1097
Volume/Å^3	5167.62(19)	Final R indexes [all data]	R_1 = 0.0478, wR_2 = 0.1136
Z	8	Largest diff. peak/hole / e Å^{-3}	0.36/-0.30

Single-crystal X-ray structure analysis of 9:

Single crystals were obtained by slow diffusion of Et_2O into a solution of **9** in DCM. A single-crystal X-ray structure analysis revealed that **9** crystallizes in the monoclinic space group $P2_1/c$. The asymmetric unit contains two molecules of **9** and one molecule of CH_2Cl_2. One pyridinyl-4-ylidenamino substituent is disordered over two positions (occupancies: 83% and 17%) and the CH_2Cl_2 molecules is distorted over three positions (occupanccies: 50%, 35% and 15%).

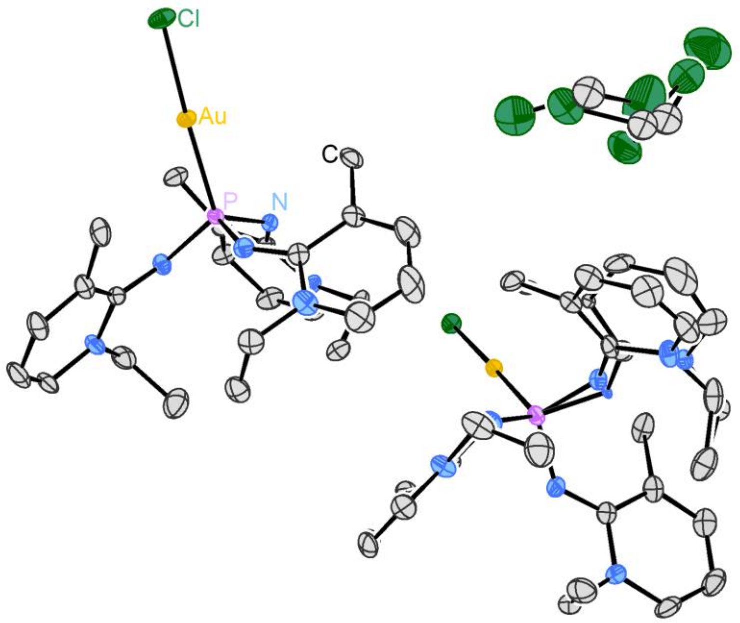

Figure S89: Molecular view of **9** in the solid state with thermal ellipsoid plot at the 50% levels of probability. Hydrogen atoms are omitted for clarity.

Table S5. Crystal data and structure refinement for **9**.

CCDC number	1950842	ρ_{calc}mg/mm^3	1.699
Empirical formula	$C_{24}H_{33}AuClN_6P$•½CH_2Cl_2	m/mm^{-1}	5.562
Formula weight	711.41	F(000)	2808.0
Temperature/K	100(2)	Crystal size/mm^3	0.33 × 0.31 × 0.19
Crystal system	monoclinic	Radiation	MoKα (λ = 0.71073)
Space group	$P2_1/c$	2Θ range for data collection	3.172 to 61.104
a/Å	19.2900(4)	Index ranges	$-27 \leq h \leq 27$, $-24 \leq k \leq 24$, $-23 \leq l \leq 24$
b/Å	17.2883(4)	Reflections collected	97893
c/Å	16.7918(4)	Independent reflections	17006 [R_{int} = 0.0685, R_{sigma} = 0.0476]
α/°	90	Data/restraints/parameters	17006/36/756
β/°	96.4900(10)	Goodness-of-fit on F^2	1.008
γ/°	90	Final R indexes [I>=2σ (I)]	R_1 = 0.0376, wR_2 = 0.0754
Volume/Å^3	5564.0(2)	Final R indexes [all data]	R_1 = 0.0557, wR_2 = 0.0816
Z	8	Largest diff. peak/hole / e Å^{-3}	2.99/-2.04

Single-crystal X-ray structure analysis of 10:

Single crystals were obtained by slow diffusion of Et_2O in a solution of **10** in DCM. A single-crystal X-ray structure analysis revealed that **10** crystallizes in the monoclinic space group $P2_1/n$. The asymmetric unit contains two molecules of **10**.

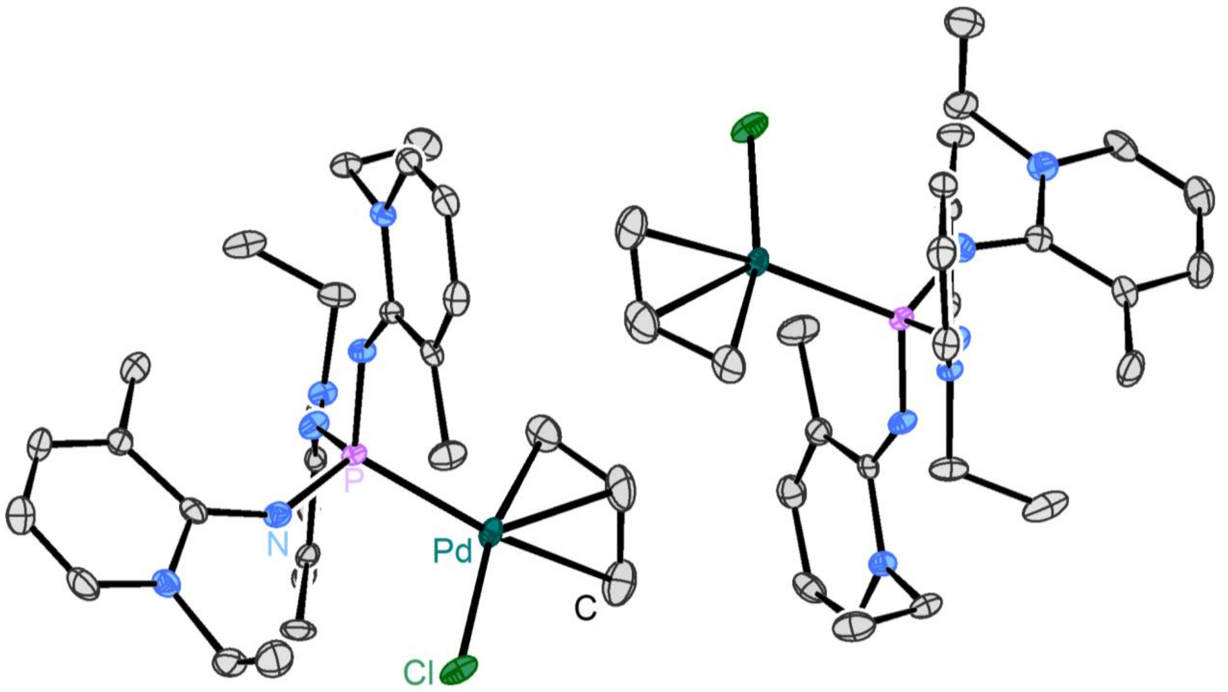

Figure S90: Molecular view of **10** in the solid state with thermal ellipsoid plot at the 50% levels of probability. Hydrogen atoms are omitted for clarity.

Table S6. Crystal data and structure refinement for **10**.

CCDC number	1950843	ρ_{calc}mg/mm^3	1.435
Empirical formula	$C_{27}H_{38}ClN_6PPd$	m/mm^{-1}	0.823
Formula weight	619.45	F(000)	2560.0
Temperature/K	100(2)	Crystal size/mm^3	0.173 × 0.082 × 0.064
Crystal system	monoclinic	Radiation	MoKα (λ = 0.71073)
Space group	$P2_1/n$	2Θ range for data collection	2.348 to 52.814°
a/Å	18.0270(7)	Index ranges	$-22 \leq h \leq 22$, $-12 \leq k \leq 12$, $-41 \leq l \leq 41$
b/Å	9.8355(4)	Reflections collected	74925
c/Å	33.5509(13)	Independent reflections	11777 [R_{int} = 0.0971, R_{sigma} = 0.0609]
α/°	90	Data/restraints/parameters	11777/0/661
β/°	105.354(2)	Goodness-of-fit on F^2	1.017
γ/°	90	Final R indexes [I>=2σ (I)]	R_1 = 0.0460, wR_2 = 0.0976
Volume/Å^3	5736.4(4)	Final R indexes [all data]	R_1 = 0.0660, wR_2 = 0.1055
Z	8	Largest diff. peak/hole / e Å^{-3}	2.29/-0.69
